# “Less words, more pictures”: creating and sharing data visualizations from a remote health monitoring system with clinicians to improve cancer pain management

**DOI:** 10.3389/fdgth.2025.1520990

**Published:** 2025-04-23

**Authors:** Virginia LeBaron, Natalie Crimp, Nutta Homdee, Kelly Reed, Victoria Petermann, William Ashe, Leslie Blackhall, Bryan Lewis

**Affiliations:** ^1^ Department of Research, University of Virginia School of Nursing, Charlottesville, VA, United States; ^2^ Center for Research Innovation and Biomedical Informatics, Faculty of Medical Technology, Mahidol University, Nakhon Pathom, Thailand; ^3^ Division of Cardiovascular Medicine, Center for Advanced Medical Analytics, University of Virginia School of Medicine, Charlottesville, VA, United States; ^4^ Division of General Medicine, Geriatrics, and Palliative Care, University of Virginia School of Medicine, Charlottesville, VA, United States; ^5^ University of Virginia Biocomplexity Institute, Charlottesville, VA, United States

**Keywords:** cancer, palliative care, pain management, data visualization, remote health monitoring and digital health, patient and caregiver dyads

## Abstract

**Background:**

The Behavioral and Environmental Sensing and Intervention for Cancer (BESI-C) is a remote health monitoring system (RHMS) developed by our interdisciplinary team that collects holistic physiological, behavioral, psychosocial, and contextual data related to pain from dyads of patients with cancer and their family caregivers via environmental and wearable (smartwatch) sensors.

**Methods:**

R, Python, and Canva software were used to create a series of static and interactive data visualizations (e.g., visual representations of data in the form of graphs, figures, or pictures) from de-identified BESI-C data to share with palliative care clinicians during virtual and in-person 1-hour feedback sessions. Participants were shown a sequence of 5–6 different data visualizations related to patient and caregiver self-reported pain events, environmental factors, and quality of life indicators, completed an electronic survey that assessed clarity, usefulness, and comprehension, and then engaged in a structured discussion. Quantitative survey results were descriptively analyzed and “think aloud” qualitative comments thematically summarized and used to iterate data visualizations between feedback sessions.

**Results:**

Six to 12 interdisciplinary palliative care clinicians from an academic medical center, a local hospice, and a community hospital within Central Virginia participated in five data visualization feedback sessions. Both survey results and group discussion feedback revealed a preference for more familiar, simpler data visualizations that focused on the physical aspects of pain assessment, such as number of high intensity pain events and response to pharmacological interventions. Preferences for degree of data granularity and content varied by discipline and care delivery model, and there was mixed interest in seeing caregiver reported data. Overall, non-physician participants expressed greater interest in visualizations that included environmental variables impacting pain and non-pharmacological interventions.

**Conclusion:**

Clinicians desired higher-level (i.e., less granular/detailed) views of complex sensing data with a “take home” message that can be quickly processed. Orienting clinicians to unfamiliar, contextual data sources from remote health monitoring systems (such as environmental data and quality of life data from caregivers) and integrating these data into clinical workflows is critical to ensure these types of data can optimally inform the patient's plan of care. Future work should focus on customizing data visualization formats and viewing options, as well as explore ethical issues related to sharing data visualizations with key stakeholders.

## Introduction

Despite decades of policy and practice efforts, an estimated 40%–90% of patients with cancer continue to experience moderate to severe pain (1–10). Even terminally ill patients with cancer enrolled in home hospice programs, which are uniquely designed to provide comprehensive support at the end-of-life, can experience poorly managed symptoms (11–14). The impact of inadequately managed cancer pain is well documented, negatively affecting sleep, adherence to treatment, mood and overall quality of life—for both patients and family caregivers (2, 15–20). Most cancer pain is chronic (lasting longer than 3 months), punctuated by acute pain episodes, commonly referred to as “breakthrough pain”. Breakthrough cancer pain is defined as a transient exacerbation of pain that “breaks through” a background of generally well-controlled pain (21); it can be especially distressing for patients and caregivers (22, 23) and contribute to unplanned healthcare utilization/emergency department visits, which may not be compatible with patient goals at the end of life (4, 24–27). Additionally, most cancer pain management occurs at home (15, 28, 29) with family caregivers often playing a crucial role in this task, especially as patients experience disease progression (16, 17, 29, 30). We also know there is a dyadic (reciprocal) and dynamic dimension to patient and caregiver distress (31–37); however, a better understanding of these relationships are essential to inform effective interventions (15), especially regarding pain management (38).

A related key gap is communicating the complex experience of cancer pain in the home context to busy clinicians in ways that are most helpful to inform the plan of care and improve health outcomes. Too often, patients and caregivers seen in the outpatient setting are asked, “how has the pain been over the past few weeks?” These well-intended assessment questions unfortunately present patients and caregivers with the daunting task of not only remembering—but efficiently and effectively summarizing—the most salient details of a highly dynamic physical and psychosocial symptom experience.

Remote health monitoring systems (RHMS) have tremendous potential to extend the reach of healthcare, enhance symptom and pain management outside traditional healthcare settings, and support clinicians in developing an effective plan of care (39). RHMS take many forms, but broadly involve the use of mobile, wearable, and wireless devices to monitor and share health-related data, most commonly with clinicians, but also with patients and caregivers (39, 40). RHMS are increasingly being deployed for multiple health conditions (41), including cancer (42–51). Visually representing RHMS generated data in an understandable and meaningful way can help inform care decisions, tailor and personalize care, and improve care outcomes (52); however, how to best create effective data visualizations from large amounts of complex, heterogeneous RHMS data is unclear (52, 53) and a critical research need (53–56). For the purposes of this paper, we define “data visualization” as the visual representation of scientific data in the form of graphs, plots, and pictures (57). Representing RHMS data with effective visualizations can facilitate self-efficacy in pain management, not only for patients and caregivers, but also for clinicians who may feel uncertain about how to guide patients and caregivers in the management of difficult pain (58, 59). Significant work related to data visual analytics has been done in chronic care disease management, such as diabetes (60–62); to our knowledge this would be the first exploration of data visualizations specifically related to advanced cancer pain from the dyadic perspective of patients and family caregivers. As RHMS rapidly become more ubiquitous (63), along with the concurrent use of clinician dashboards/platforms to view collected data (64–66), it is critical to understand how to best share output from such systems with clinicians to improve health outcomes and strengthen communication between clinicians, patients, and caregivers. In this paper, we discuss an approach to creating and sharing data visualizations generated from a novel remote health monitoring system, Behavioral and Environmental Sensing and Intervention for Cancer (BESI-C), with palliative care clinicians related to pain experienced at home by patients with advanced cancer.

### Overview of the BESI-C remote health monitoring system

**Figure 1:**
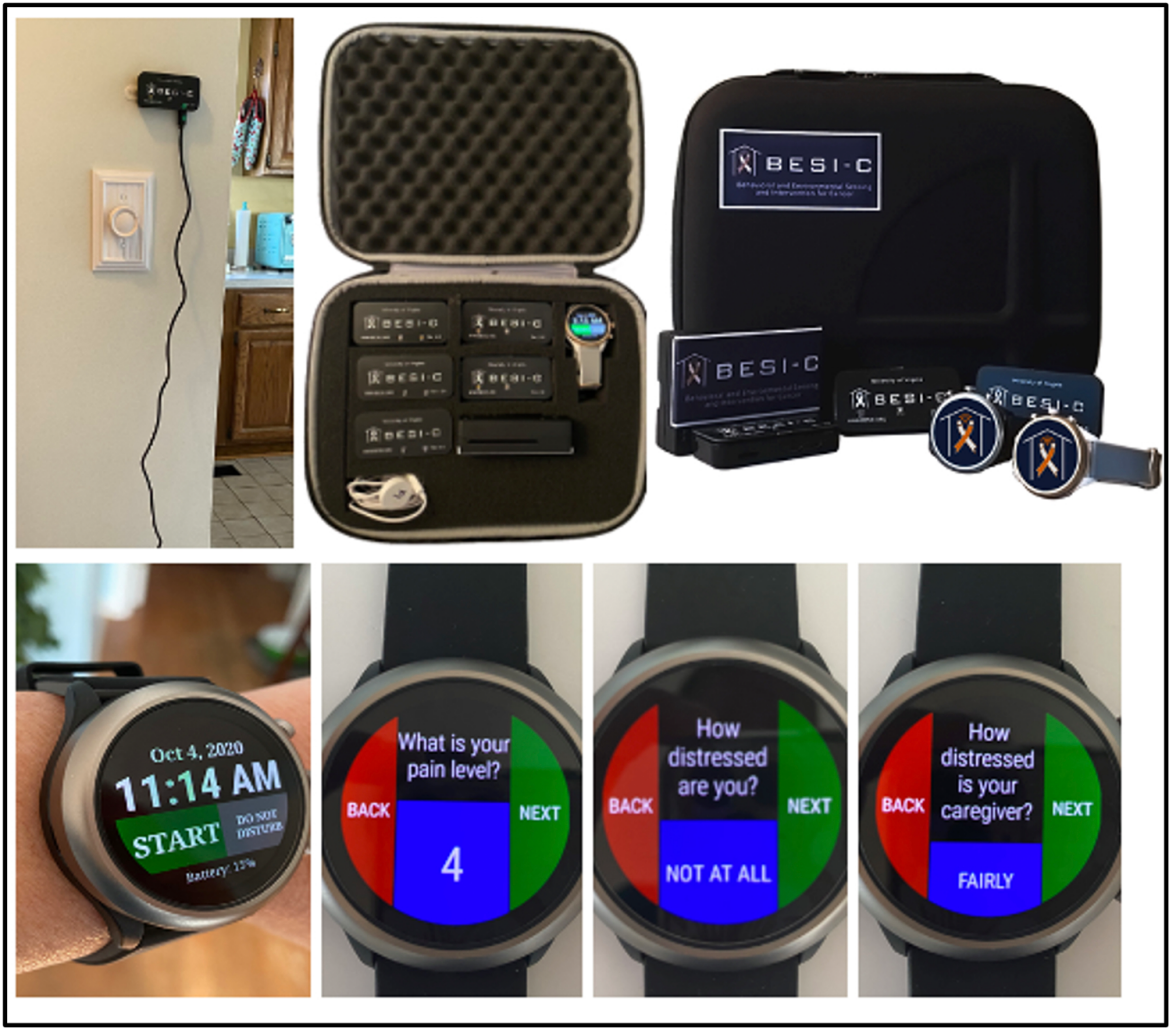
BESI-C system components: Top, environmental sensor and “BESI Box”; bottom, BESI-C custom wearable application and examples of ecological momentary assessments (EMAs).

**Figure 2:**
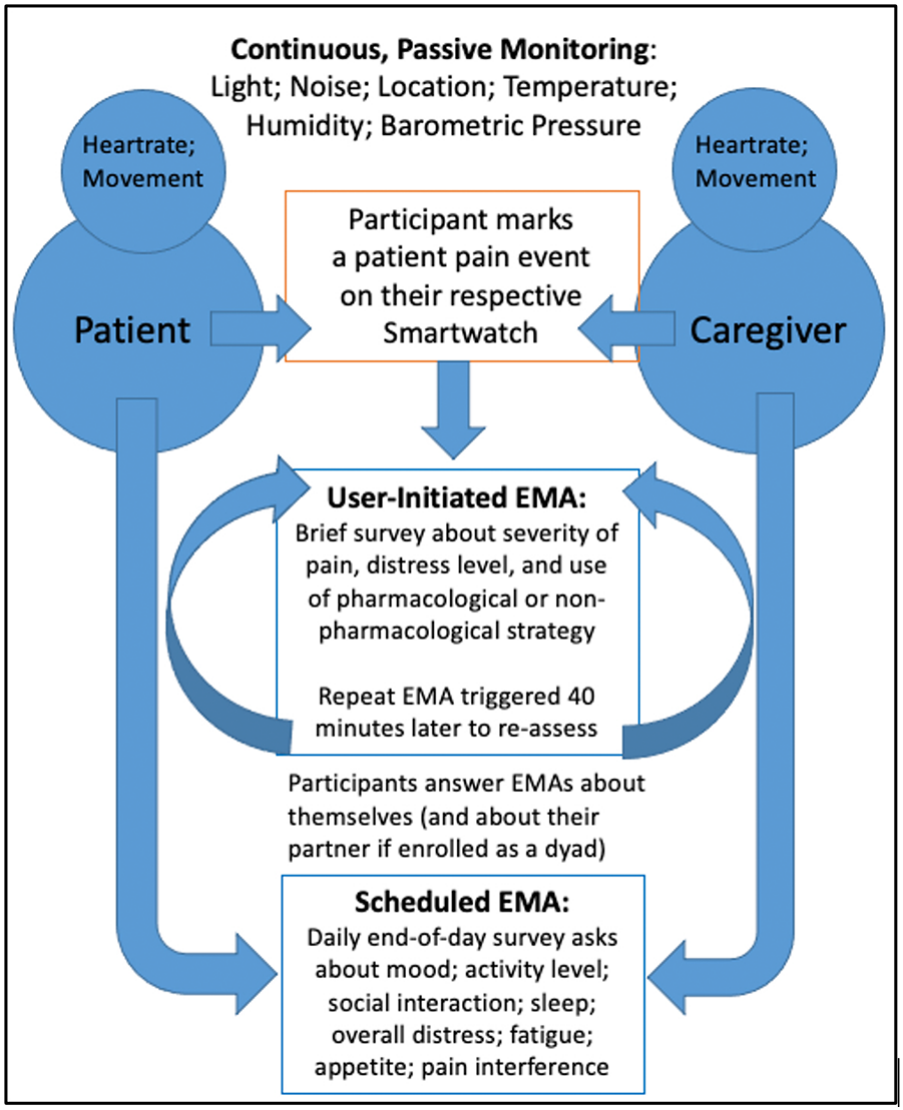
The BESI-C assessment model. Reprinted with permission and in accordance with the terms of the Creative Commons Attribution License (https://creativecommons.org/licenses/by/4.0/), which permits unrestricted use, distribution, and reproduction in any medium, provided the original work, first published in JMIR Research Protocols (67), is properly cited: JMIR Res Protoc. 2019 Dec 9;8(12):e16178. doi: 10.2196/16178.

Behavioral and Environmental Sensing and Intervention for Cancer (BESI-C) is an innovative RHMS developed by our interdisciplinary team to monitor, and ultimately manage, cancer pain in the home setting by delivering personalized, “just in time” interventions. We have reported previously on our user-centered design process (67, 68), initial feasibility and acceptability testing (69), and pilot study results (70–72). Briefly, BESI-C collects heterogenous sensing data from patients, caregivers, and the ambient home environment using a combination of wearable (smartwatch) and environmental sensors. The system is deployed as a “BESI-Box” (Figure 1) which is shipped to participants, self-installed and used for approximately 14 days. Both patients and caregivers are asked to wear a smartwatch programmed with the custom BESI-C application which allows them to record and characterize patient pain events and other quality of life information via user-initiated (i.e., on-demand) and scheduled Ecological Momentary Assessments [EMAs, brief surveys delivered on mobile devices in real-world settings (73); Figure 2]. When a patient or caregiver records a pain event on their respective smart watch, BESI-C provides a comprehensive “snapshot” of what is occurring at, and around, the time of the event. A unique feature of BESI-C is the breadth of data collected from both patients and caregivers, including physiological, psychosocial, behavioral, and contextual data that can be used to inform and train personalized models to deliver real-time notifications for early intervention.

## Materials and methods

### Overview

Findings presented in this paper represent one aim of our study that deploys BESI-C to characterize a “digital phenotype” of advanced cancer pain in the home context (74). This paper describes our multi-method approach to collect both qualitative and quantitative feedback from clinicians regarding data visualizations generated by the BESI-C remote health monitoring system (RHMS). Approval was obtained from the University of Virginia Health Sciences Institutional Review Board and all participants provided informed consent prior to data collection. Participants were offered a $25 gift card for each feedback session they attended to compensate them for their time.

### Data curation and collection procedures

#### Participant sample

Eligible participants included clinicians (any discipline) over the age of 18 involved in the care of patients with cancer who may experience pain. Participants were recruited from three diverse study sites in Central Virginia (academic medical center; community hospital; hospice) that served as patient-caregiver referral sites for the BESI-C study. Invitations to participate in a focus group, along with approved informed consent documents, were emailed by the PI to the study contact coordinator at each site, who then shared the information with their respective staff. If multiple data visualization feedback sessions were offered at a study site, clinicians were invited and encouraged to attend as many as they could.

#### Preparing the data visualizations

**Figure 3:**
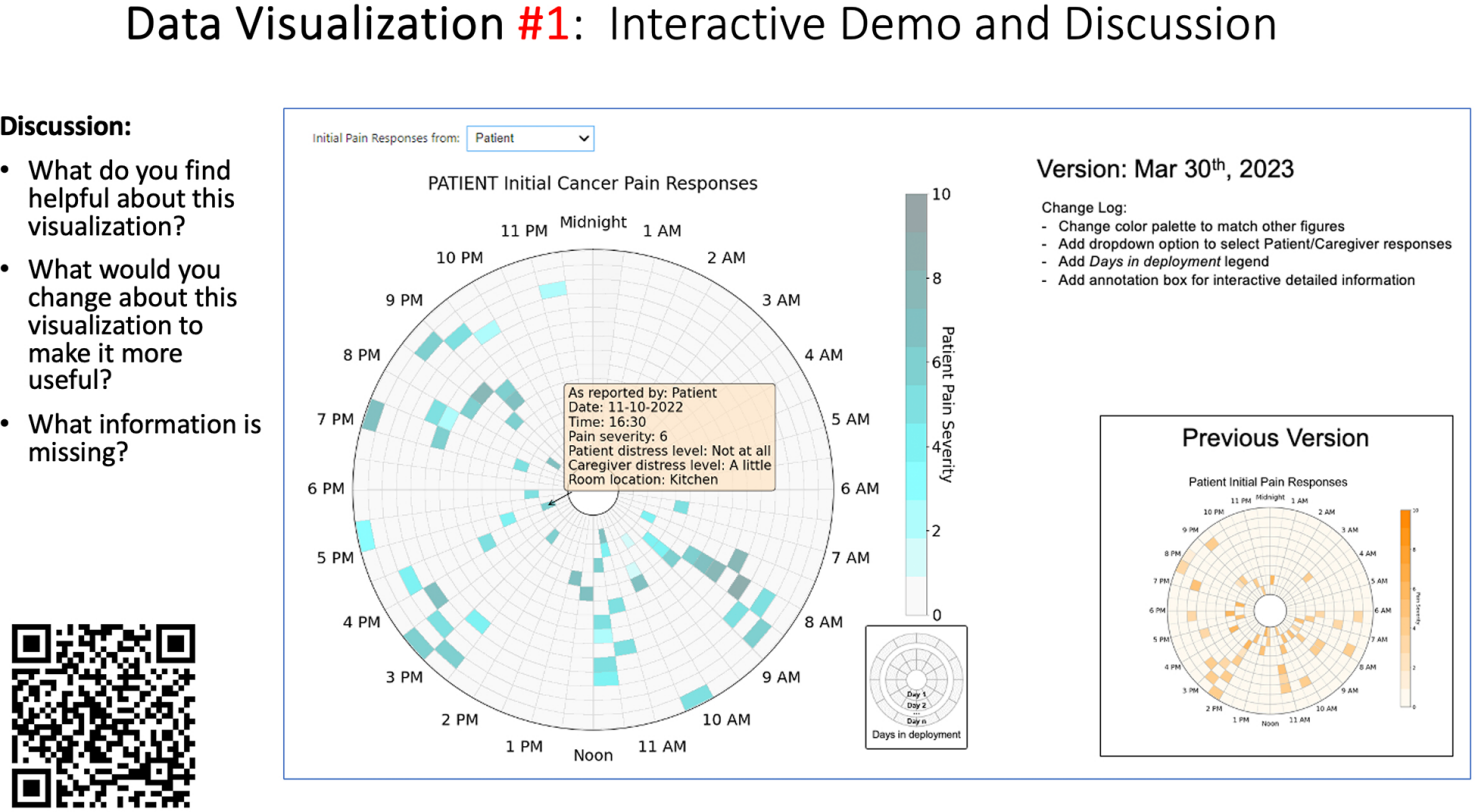
Example of data visualization slide shown at feedback session 2.

Data visualizations were created from data provided by patient and caregiver dyads who used the BESI-C system in their home for approximately 14 days. All patients had a diagnosis of locally advanced or metastatic cancer (any type of cancer); difficult cancer-related pain [documented as ≥6/10 on the pain Numeric Rating Scale (75) or as per the referring clinician]; prescribed as-needed (PRN) opioids for cancer-related pain; and could identify a primary family caregiver (“family” defined broadly as an informal care partner) who lived with them and was also willing to participate. In consultation with study site leaders, and through discussions with our interdisciplinary research team, we identified preliminary questions of interest we could answer with the BESI-C data and which data were likely to be most useful to clinicians (e.g., “how many pain events were reported over the 2-week period?”), and ideas about ways to present the data (e.g., bar chart, line graphs). Selected data features (all de-identified) were then curated from completed BESI-C deployments to generate both static and interactive data visualizations using R, Python, and Canva software. For example, with Python we used data features such as timestamps and pain severity ratings from pain reports to create circular plots to visualize time-based events, as shown in Figure 3. If users desired more details about a specific pain report, they could interact with the circular plot by hovering over an individual event to see pop-up information, such as whether an opioid was taken, or the distress level associated with that particular pain event. In later feedback sessions, in response to feedback that clinicians would like simpler visualizations, graphic design software (Canva) was used to create more infographic-style layouts. To optimize data visualization creation, we utilized our internal data quality reports to select BESI-C deployments with the highest quality data (e.g., minimal missingness). As our goal was to gather feedback on RHMS data visualizations with clinicians—vs. representing a specific dyad experience with complete fidelity—some data were imputed, amalgamated, or adjusted slightly to ensure visualization clarity and completeness. Preliminary visualizations were discussed, iterated, and refined within our research group during weekly team meetings over approximately 5 months to create a final set of initial visualizations to share with clinicians that represented unique aspects of the patient and caregiver experience. The final set of visualizations included a combination of bar charts, line graphs, circular plots, donut charts, bubble plots, and Sankey diagrams (see Supplementary Data 1).

#### Creating the feedback survey

To help gather participant demographic information and quantitative data visualization feedback during each focus group, an electronic survey (Qualtrics v.2023; Provo, Utah) was designed. The survey was designed with collaborative input from the University of Virginia Center for Survey Research and internally pilot tested and iteratively revised within our interdisciplinary research team (e.g., nurses, data scientists, biostatisticians, engineers) to ensure flow and ease of completion on a mobile device/smartphone. Given the challenges of demanding clinician schedules, it was especially important that participants could complete survey items quickly on their smartphones during our synchronous feedback sessions. Survey items were informed by the data visualization literature (54, 76, 77) and included demographic items; items assessing general preferences related to viewing symptom data; items specific to each visualization that assessed clarity, perceived usefulness, and comprehension; and a free-text write-in option for any additional comments. The survey used during each data visualization session is included as a Data Supplement (see Supplementary Data 2).

#### Conducting the feedback sessions

Based on preferences and availability of participant groups, we used two different approaches to collect feedback—either in-person or virtually over Zoom. For in-person sessions, our research team traveled to the participant study site to conduct the feedback session. All participants were asked to bring their mobile phone with them to the feedback session to facilitate participation in the electronic survey (this was not an issue for any participant; all participants had access to a smartphone). During the feedback session, participants were provided with a QR code to complete the anonymous electronic Qualtrics survey on their smartphone. Participants were shown a sequence of 5–6 different data visualizations over the 1-hour session (allotting approximately 10–12 min of discussion per visualization) related to patient and caregiver self-reported pain events and quality of life indicators. Data visualizations were presented as a screen-share slide show during virtual feedback sessions, or as individual participant paper packets (printed in color) for in-person sessions. Before reviewing any data visualizations, a brief overview of BESI-C was provided to ensure all participants had the same baseline understanding of the system and to help participants better contextualize the data sources used to create the visualizations.

Each visualization was first presented without discussion and participants independently answered survey items that assessed: (1) clarity; (2) usefulness, and (3) comprehension related to that visualization (Table 1). Then, participants were invited to engage in a structured group “think aloud” discussion that focused on three discussion prompts: (1) what did you like or find most helpful about this visualization?; (2) what would you change about this visualization?; and (3) what information is missing? At least one research assistant (distinct from the session group facilitator) attended all feedback sessions and took notes during the open discussion portion of the feedback sessions to capture the “think aloud” comments. For multiple sessions held within the same institution, we showed the previous version of the data visualization and how it was iterated/changed based on the group's prior feedback (Figure 3). Survey results and “think aloud” comments were analyzed between each session and data visualizations iterated based on participant feedback. Some iterations were significant (e.g., deciding that a visualization “missed the mark” and needed to be represented in a very different way or omitted altogether), other changes were more minor, such as changing the color palette or clarifying wording on a graph. After session 2, we created a style guide to standardize font and color palette across all visualizations.

**Table 1:** Survey items and discussion prompts used to assess each data visualization in a feedback session.

Survey items
1. This data visualization is easy to understand. 2. This data visualization would help me make clinical decisions. 3. This data visualization would save me time providing clinical care. 4. Based on my clinical experience, this data visualization would be helpful for patients and family caregivers^a^. 5. Which of the following statements is true?^b^
Discussion prompts
1. What did you like or find most helpful about this visualization? 2. What would you change about this visualization to make it more useful? 3. What information is missing?

Survey items 1–4, response options included: strongly agree; agree; disagree; strongly disagree; unsure.

^a^ Question added after feedback session 3.

^b^ Tailored comprehension question to assess understanding of a specific visualization.

#### Data analysis

Quantitative survey items were descriptively analyzed (IBM SPSS Statistics v.29; R v.4.4.0; R Studio v. 2024.04.1+748) and summary statistics generated (e.g., frequencies, counts) along with box plots and bar charts to display results. Write-in free text survey responses were few; they were exported from Qualtrics and summarized in a Word document by data visualization session and considered along with discussion data from the feedback sessions. Qualitative comments generated during the discussion portion of each session were summarized by data visualization for each session (e.g., all comments related to data visualization #1 during feedback session 1 were combined) to look for overarching themes and patterns across, and within, feedback sessions. We followed principles of qualitative descriptive analysis (78) in reviewing our free text and “think aloud” discussion comments, as our goal was not to reach a high level of abstraction with our data, but to stay well-grounded in the specific and concrete questions asked of participants related to each data visualization.

## Results

Between January 2023 and December 2023 we conducted a total of five (*n* = 5) separate data visualizations feedback sessions at our three different study (i.e., patient-caregiver dyad referral) sites. Between 6 and 12 interdisciplinary palliative care clinicians attended each feedback session and submitted an electronic survey; with a total of 47 (*n* = 47) participants across all 5 sessions. Sessions 1, 2, and 3 were all held virtually (over Zoom) with clinicians at the academic medical center referral site; session 4 was held in-person at the community hospital referral site; and session 5 was held in-person at our hospice referral site. For the virtual sessions at the academic medical center, 50% of participants (*n* = 16) reported viewing the visualizations by themselves on their individual desktop/laptop, while 50% of participants (*n* = 16) viewed the visualizations with others on a shared screen. Table 2 presents demographic sample data for each session. Across all data visualization feedback sessions, we presented a total of 28 data visualizations (22 unique visualizations that were iterated between sessions and 6 visualizations that remained essentially unchanged between sessions).

**Table 2:** Participant group self-reported demographic characteristics, per feedback session, per site.

Categories	Academic Medical Center^a^			Community Hospital	Hospice
	Session 1 *n* (%) *n* = 12	Session 2 *n* (%) *n* = 9	Session 3 *n* (%) *n* = 11	Session 4 *n* (%) *n* = 9	Session 5 *n* (%) *n* = 6
Age (years)					
25–34	2 (16.7)	2 (22.2)	2 (18.2)	1 (11.1)	2 (33.3)
35–44	4 (33.3)	3 (33.3)	4 (36.4)	2 (22.2)	1 (16.7)
45–54	2 (16.7)	2 (22.2)	1 (9.1)	2 (22.2)	3 (50)
55–64	3 (25)	1 (11.1)	3 (27.3)	3 (33.3)	0 (0)
65–74	1 (8.3)	1 (11.1)	1 (9.1)	1 (11.1)	0 (0)
Gender Identity					
Man	5 (41.7)	4 (44.4)	5 (45.5)	1 (11.1)	0 (0)
Woman	7 (58.3)	5 (55.6)	5 (45.5)	7 (77.8)	6 (100)
Non-binary	0 (0)	0 (0)	1 (9.1)	0 (0)	0 (0)
Prefer not to answer	0 (0)	0 (0)	0 (0)	1 (11.1)	0 (0)
Race and Ethnicity^b^					
White	9 (75)	7 (77.8)	6 (54.5)	8 (88.9)	6 (100)
Black or African American	1 (8.3)	0 (0)	0 (0)	0 (0)	0 (0)
Asian	2 (16.7)	2 (22.2)	5 (45.5)	0 (0)	0 (0)
Prefer not to answer	0 (0)	0 (0)	0 (0)	1 (11.1)	0 (0)
Clinical Role					
Registered nurse (RN)	2 (16.7)	2 (22.2)	2 (18.2)	4 (44.4)	3 (50)
Physician (MD, DO)	6 (50)	5 (55.6)	9 (81.8)	0 (0)	1 (16.7)
Advanced practice registered nurse (NP, CNS)	2 (16.7)	2 (22.2)	0 (0)	3 (33.3)	1 (16.7)
Social worker	2 (16.7)	0 (0)	0 (0)	1 (11.1)	0 (0)
Other (not specified)	0 (0)	0 (0)	0 (0)	1 (11.1)	1 (16.7)
Years of Clinical Oncology Experience					
Less than 1 year	2 (16.7)	1 (11.1)	1 (9.1)	0 (0)	0 (0)
1–5 years	2 (16.7)	2 (22.2)	2 (18.2)	4 (44.4)	0 (0)
6–11 years	3 (25)	4 (44.4)	4 (36.4)	0 (0)	3 (50)
More than 11 years	5 (41.7)	2 (22.2)	4 (36.4)	4 (44.4)	3 (50)
Missing	0 (0)	0 (0)	0 (0)	1 (0.11)	0 (0)

RN, registered nurse; MD, doctor of medicine; DO, doctor of osteopathic medicine; NP, nurse practitioner; CNS, clinical nurse specialist.

^a^ Total sample demographic data are not presented as the same individual could have attended multiple sessions for the Academic Medical Center sessions 1–3.

^b^ Please see Supplementary Data 2 for specific survey items regarding participant self-reported race and ethnicity.

### Quantitative results

Surveys results related to: (1) perceived clarity, usefulness, and comprehension of each visualization; (2) general data viewing preferences; and (3) priorities related to types of data included in potential data visualizations are presented below.

#### Perceived clarity, usefulness, and comprehension of shared data visualizations

Tables 3–7 (columns A and C) show the specific data visualizations shared at each feedback session and the proportion of participants who agreed or disagreed with statements related to clarity and usefulness. Overall, reception to the data visualizations improved from session 1 to session 5, with more participants agreeing that the visualizations were clear and useful. Bar chart and line graphs were generally more favorably received compared to less familiar data visualizations, such as the Sankey diagram (Table 3, data visualization #3; Table 4, data visualization #6) and bubble plot (Table 3, data visualization #4; Table 4, data visualization #5)—but interestingly, did not always fare as well with the corresponding comprehension question (Tables 3 and 4, column B). For example, in session 2, data visualization #2 (Table 4)—the stacked bar graph—89% and 78% agreed that the visualization was “easy to understand” and “would help make clinical decisions”, respectively,—but only 67% of participants answered the comprehension question about this visualization correctly. Relatedly, in session 4 (Table 6), data visualization #6—the circular pain wheel plot—50% of participants disagreed that it was clear or useful, but 100% of participants answered the comprehension question correctly. Data visualizations that received 75%, or less, accuracy with their corresponding comprehension question included: session 1, data visualization #5; session 2, data visualizations #1, #2, and #4; session 3, data visualization #5; session 4, data visualization #3; and session 5, data visualization #4 (Tables 3–7, column B).

**Table 3:** Feedback session 1—Academic Medical Center.

A: Data Visualization	B: Comprehension Question	C: Quantitative Feedback	D: Qualitative Feedback
	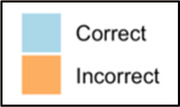	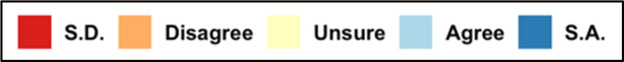 S.D. = Strongly Disagree S.A. = Strongly Agree	What do you find helpful?/ What do you like?
			What would you change?/What is missing?/What questions do you have?
Data Visualization—#1			
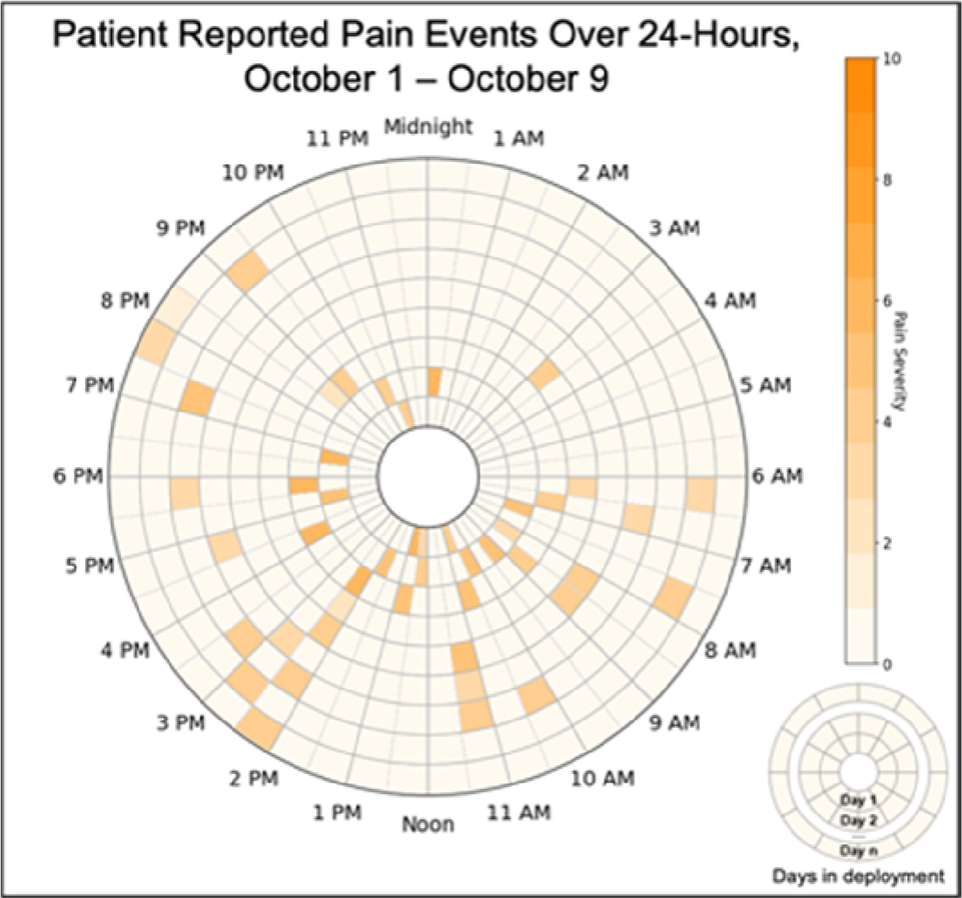	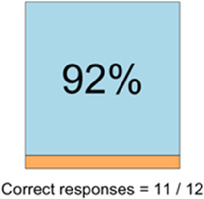	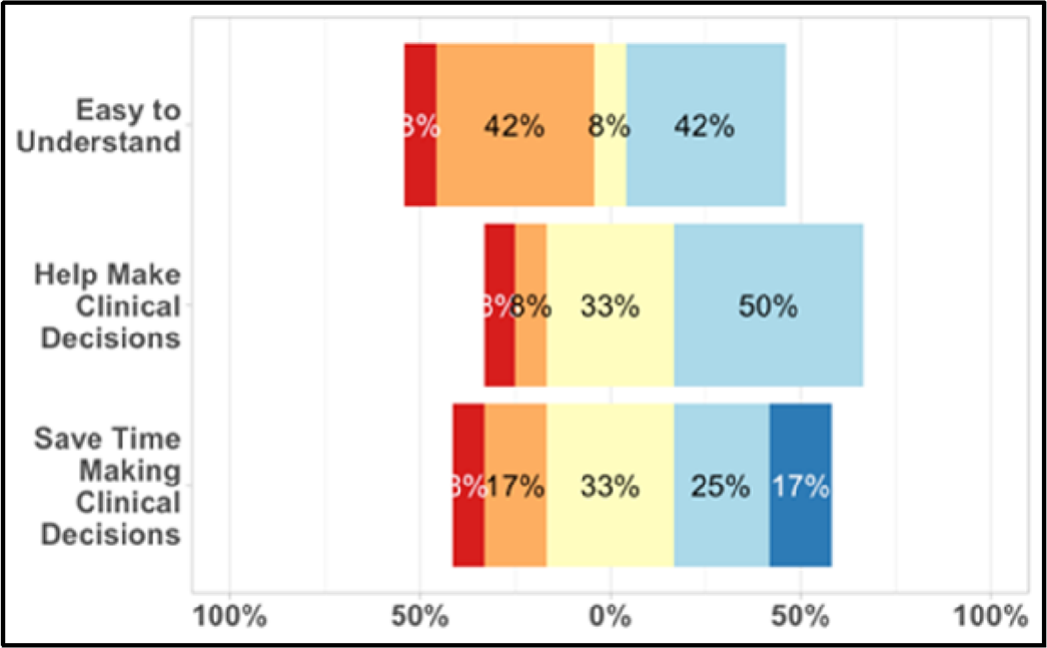	• Provides a helpful “bird's eye view” • Once it was explained, I liked it • Like that you can see the time of day where there were the most pain events
			• Too busy • Difficult to understand at first, not intuitive • Helpful if we could see what times people always need pain medication
Data Visualization—#2			
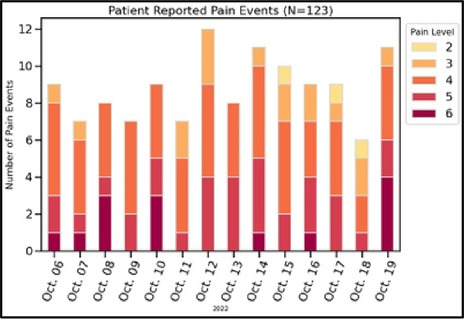	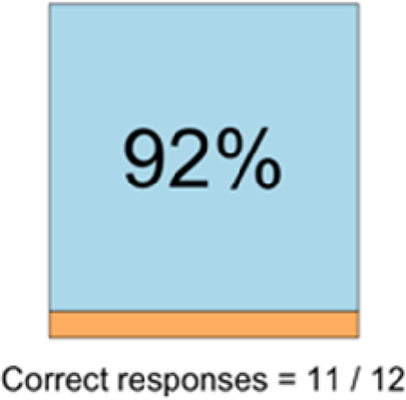	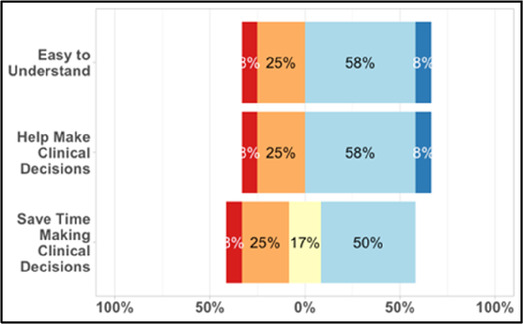	• Easy to see how many uncontrolled pain events • Easy to understand
			• Focus on uncontrolled or severe pain (i.e., events ≥5/10) • Would like to see more contextual data, e.g., what was happening on a high pain day?
Data Visualization—#3			
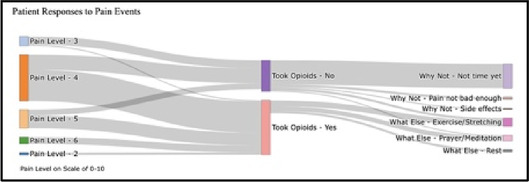	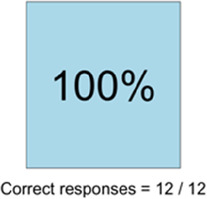	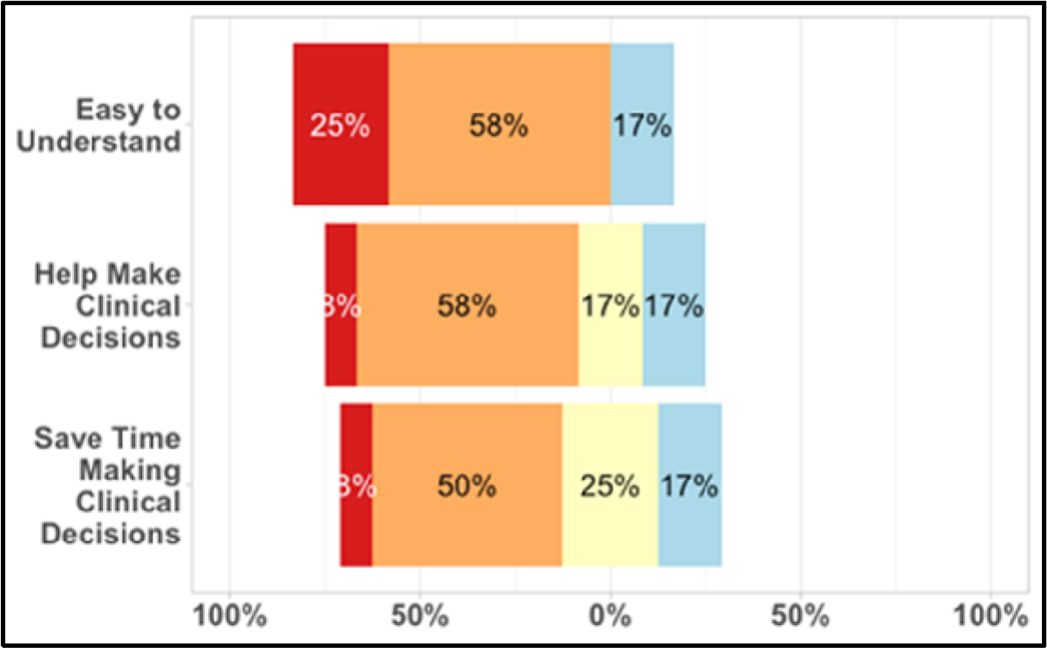	• No responses.
			• Took too long to understand for the value provided • List the pain levels in numerical order • Too many colors; have the colors follow through vs. all of the gray; change “yes opioids” to green and “no opioids” to red
Data Visualization—#4			
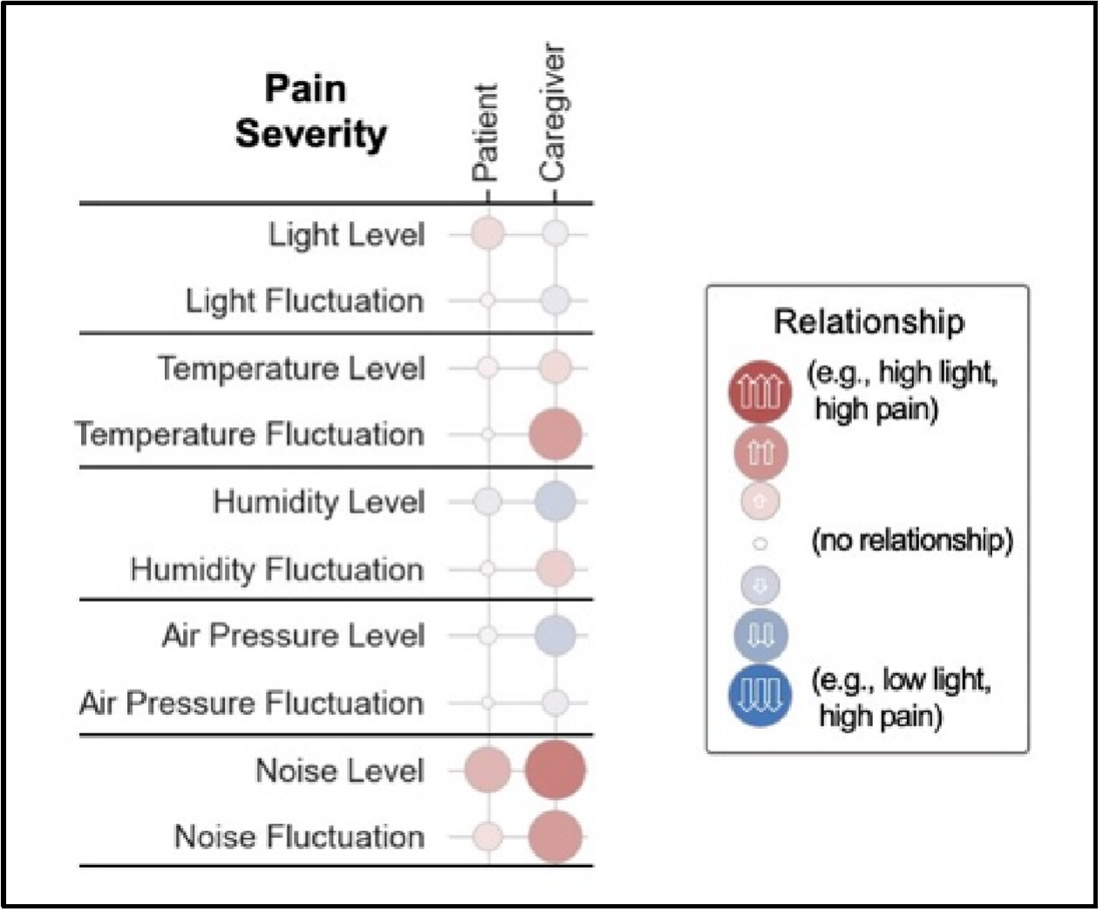	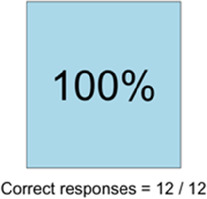	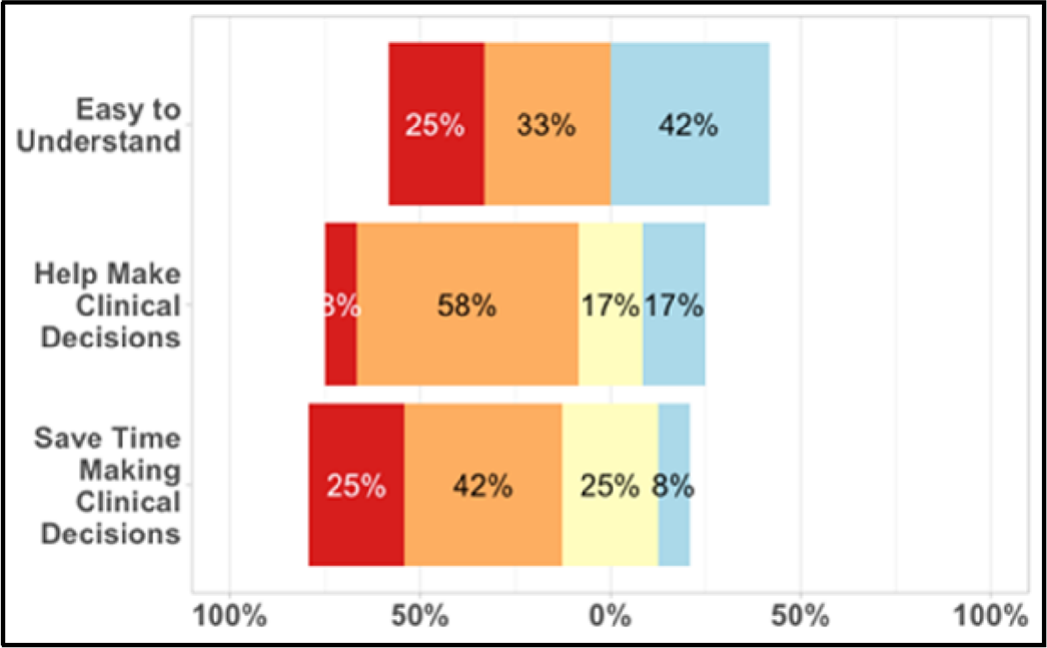	• Teaching opportunity for nurses/social workers; provides information on modifiable factors they can communicate to patients and/or caregivers • More helpful for patients/caregivers vs. clinicians as these factors are outside clinician control
			• Too much data, didn’t understand it • Didn’t understand the legend; is the circle bigger as pain severity gets worse? • Could the color intensity correlate with higher pain?
Data Visualization—#5			
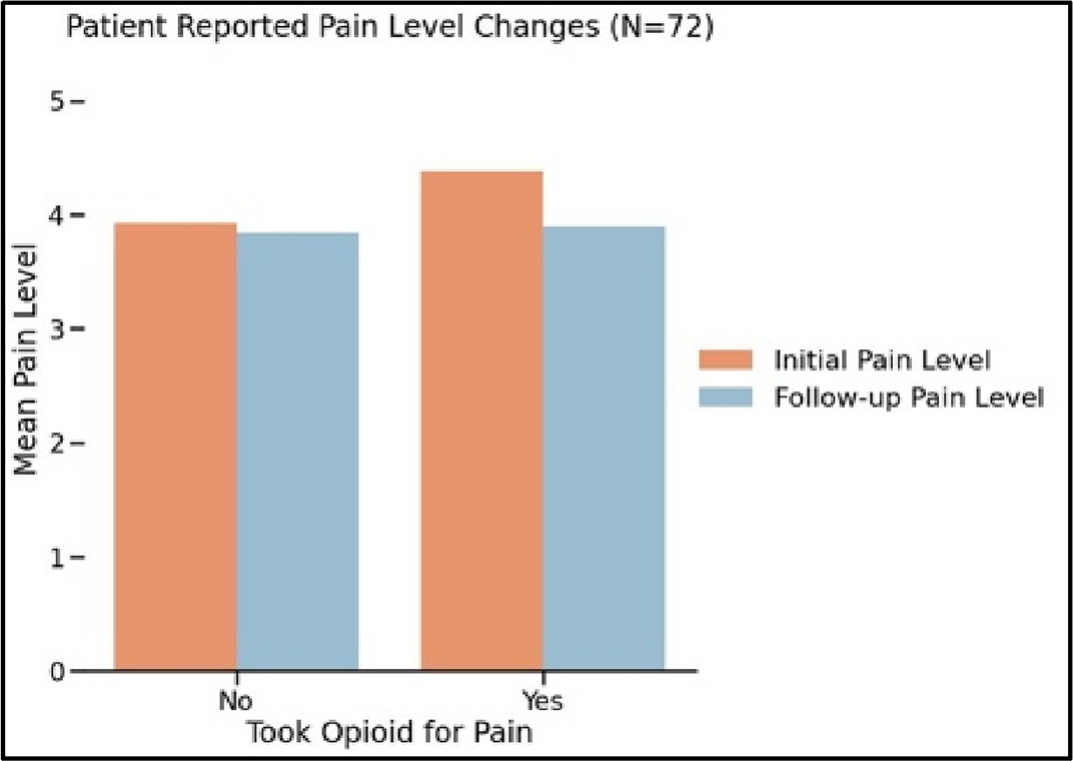	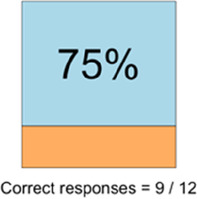	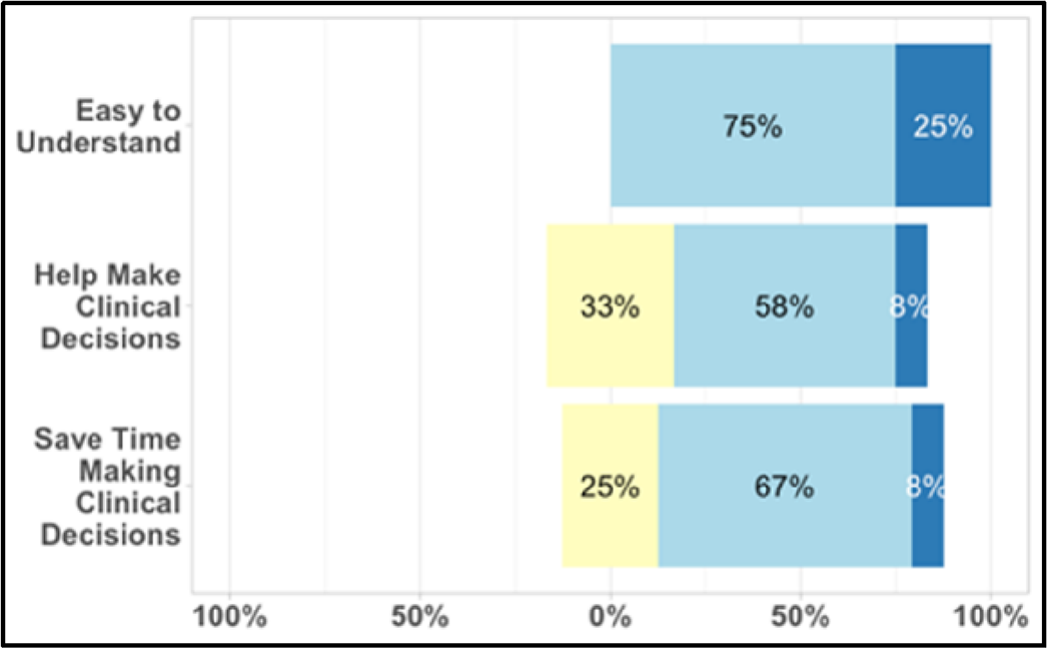	• Found it helpful, but would be more helpful if this person had higher pain
			• Would prefer to only see the highest pain events vs. averaging pain events, and then look at what they do for that pain and if it helps

**Table 4:** Feedback session 2—Academic Medical Center.

A: Data Visualization	B: Comprehension Question	C: Quantitative Feedback	D: Qualitative Feedback
	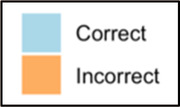	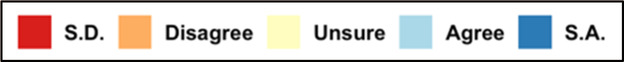 S.D. = Strongly Disagree S.A. = Strongly Agree	What do you find helpful?/ What do you like?
			What would you change?/What is missing?/What questions do you have?
Data Visualization—#1			
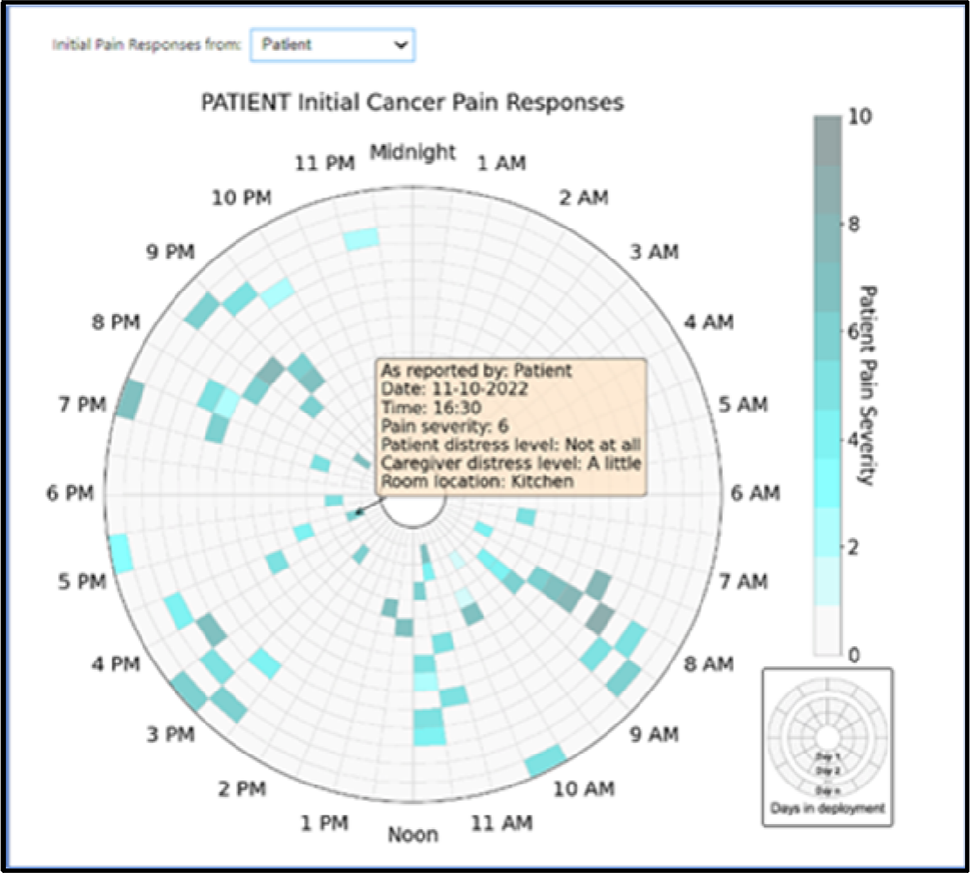	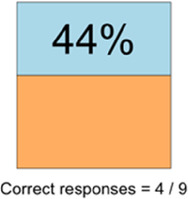	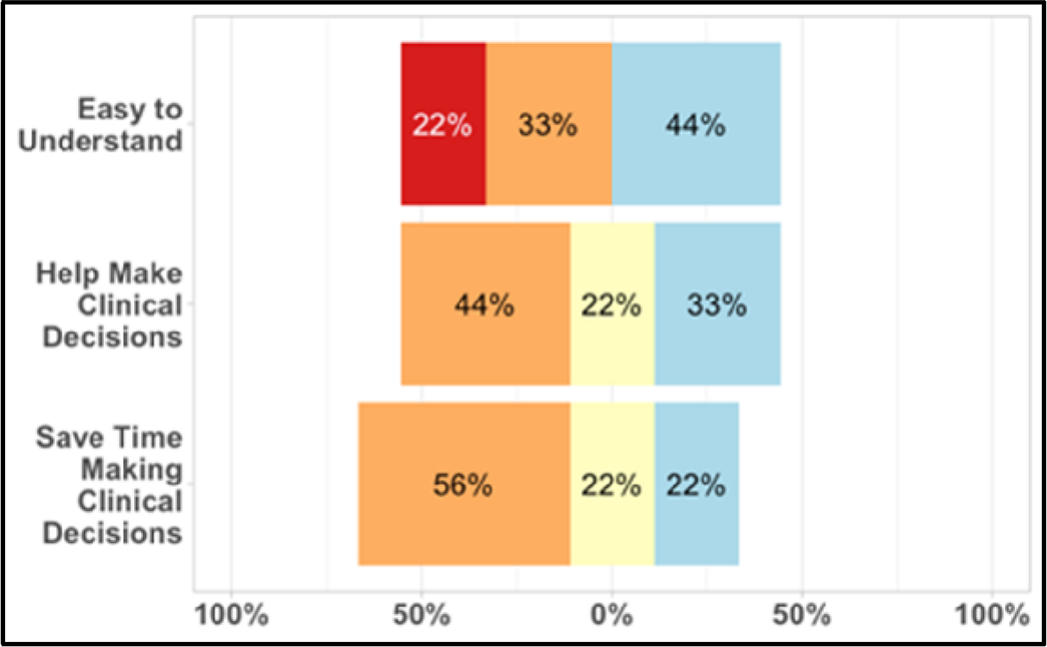	• Like the fact that it is similar to the rings on an Apple Watch • Can effectively see the most painful times of each day; and that the person was consistently able to sleep well without interruptions from pain
			• This is not an efficient way to get information • Wish it wasn’t so busy or complicated; wish colors were more intuitive • Who is in distress? Is the caregiver in distress? always need pain medication
Data Visualization—#2			
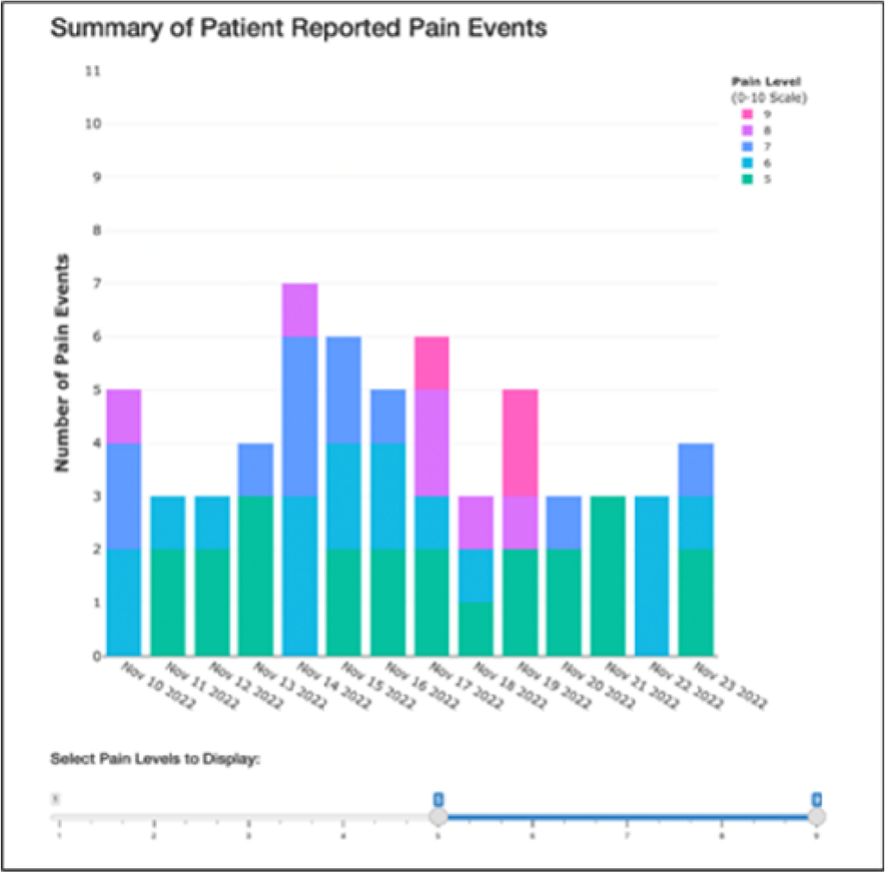	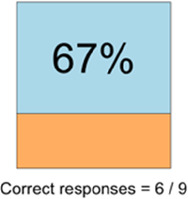	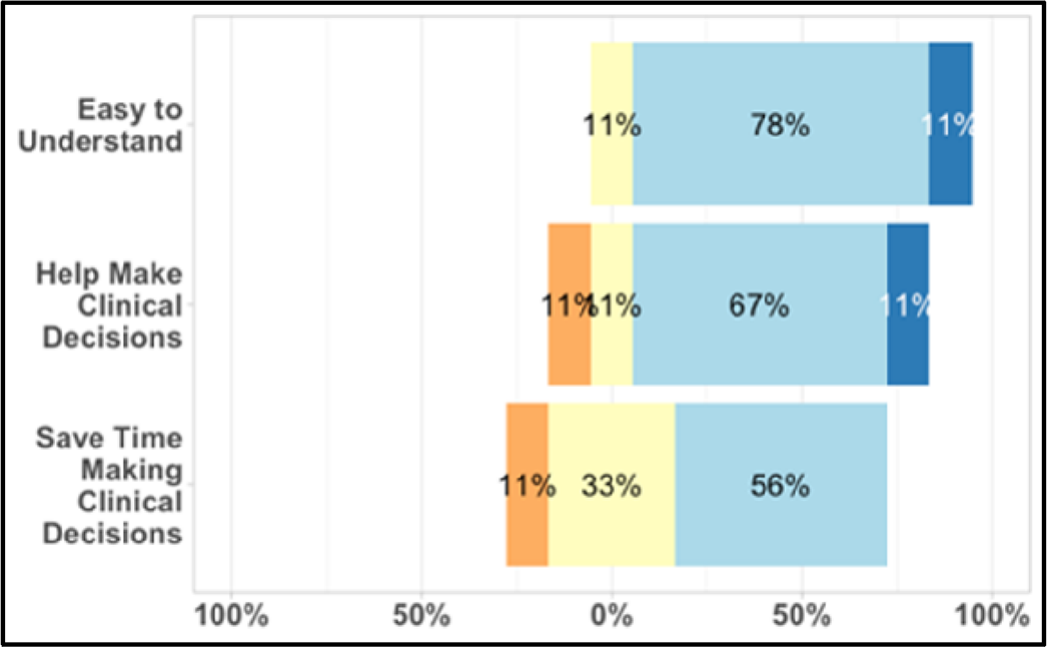	• Like this graph a lot; more quickly able to get info; did not need to decipher; like the interactive component • Being able to see particular pain severity events is helpful; it was clear when the patient had pain
			• Still busy (though not terribly busy)
Data Visualization—#3			
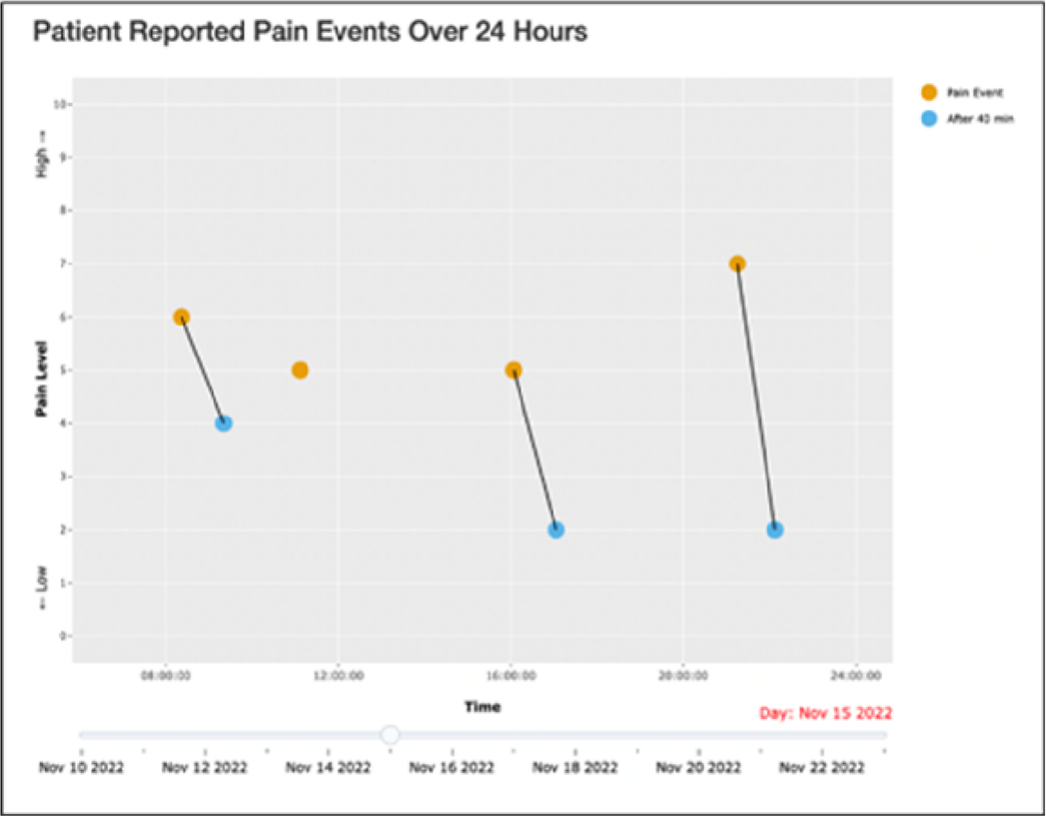	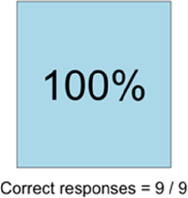	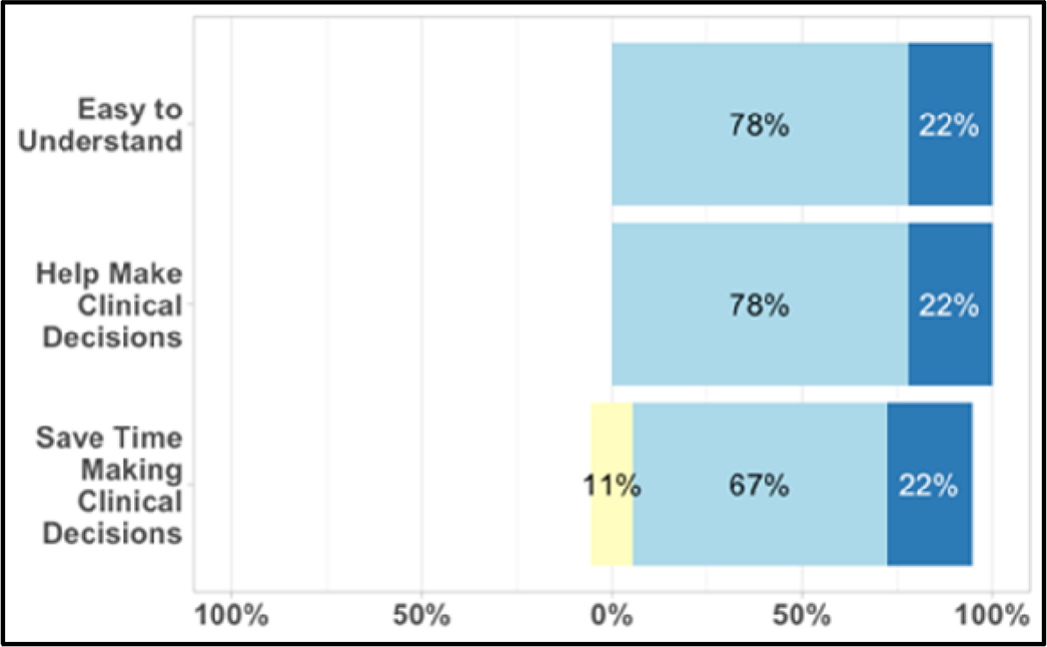	• Able to see what works for the patient and what doesn’t.
			• Would be helpful to see the timing of the opioid, e.g., how long after the initial event was medication taken?
Data Visualization—#4			
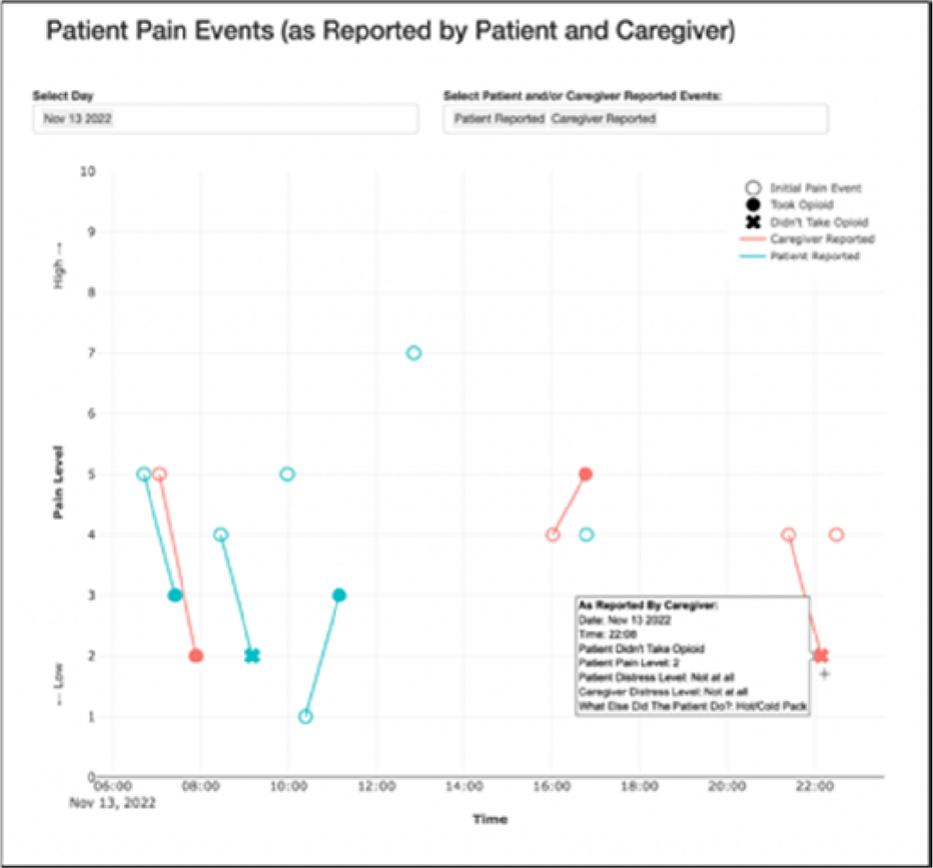	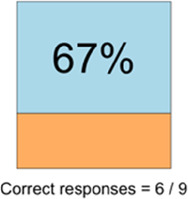	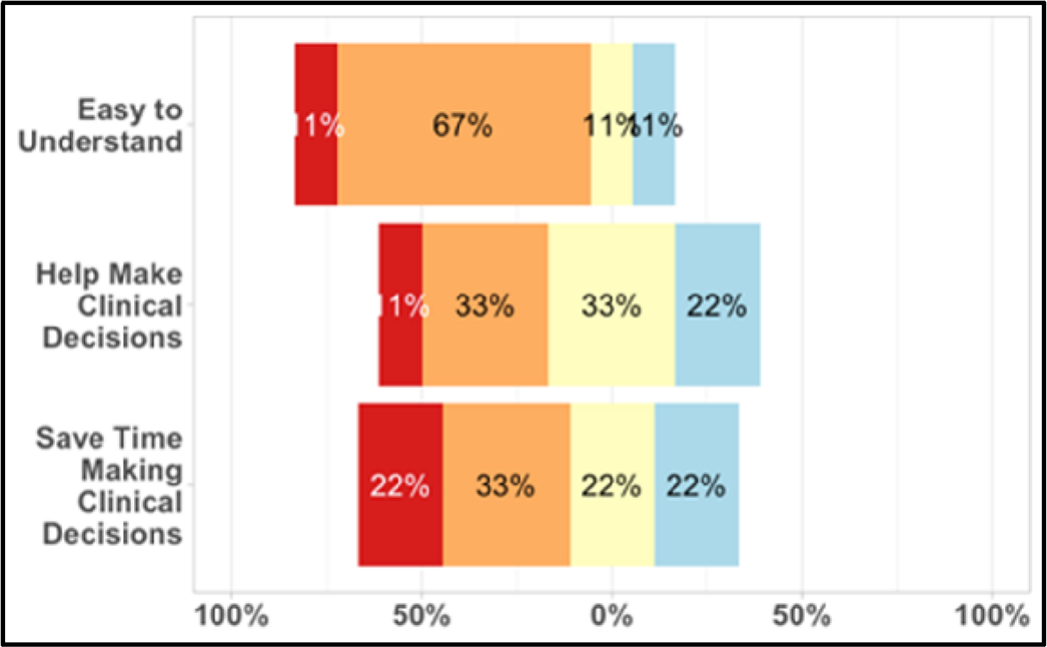	• The caregiver information can be helpful, particularly depending on the patient
			• Difficult to see a trend; too much information to follow • Wish there wasn’t the need to reference a legend
Data Visualization—#5			
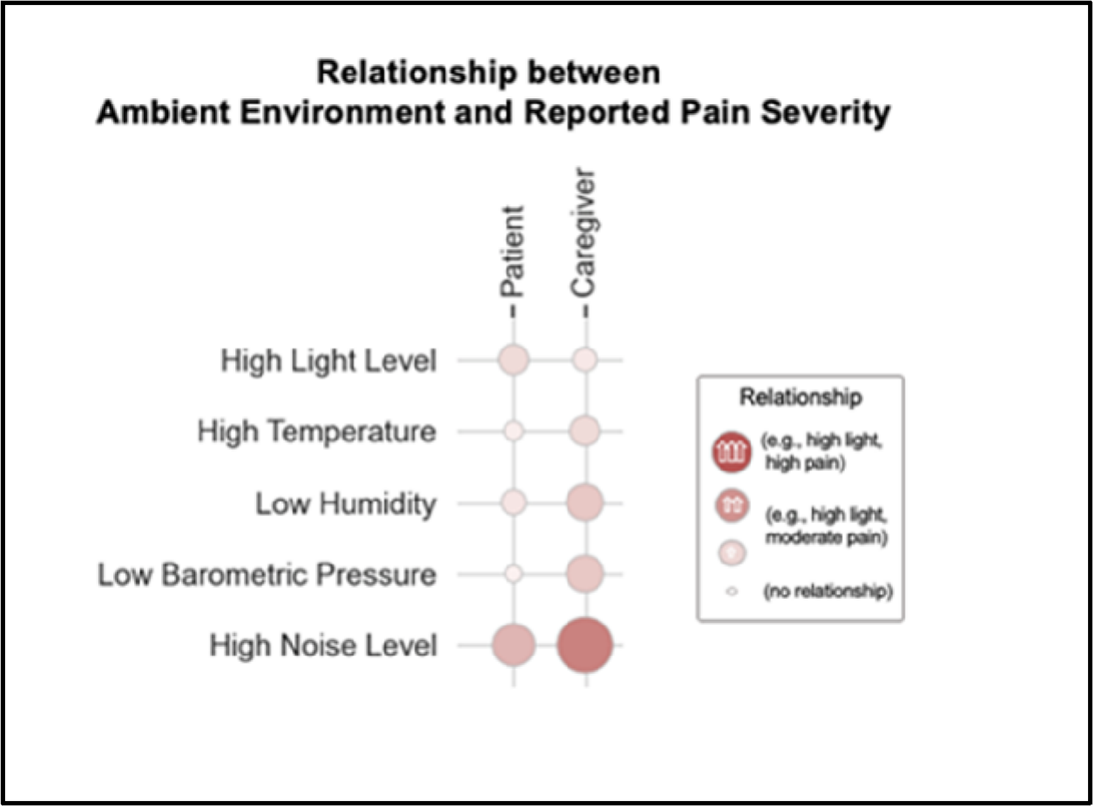	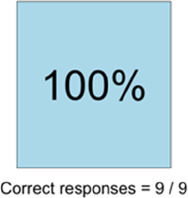	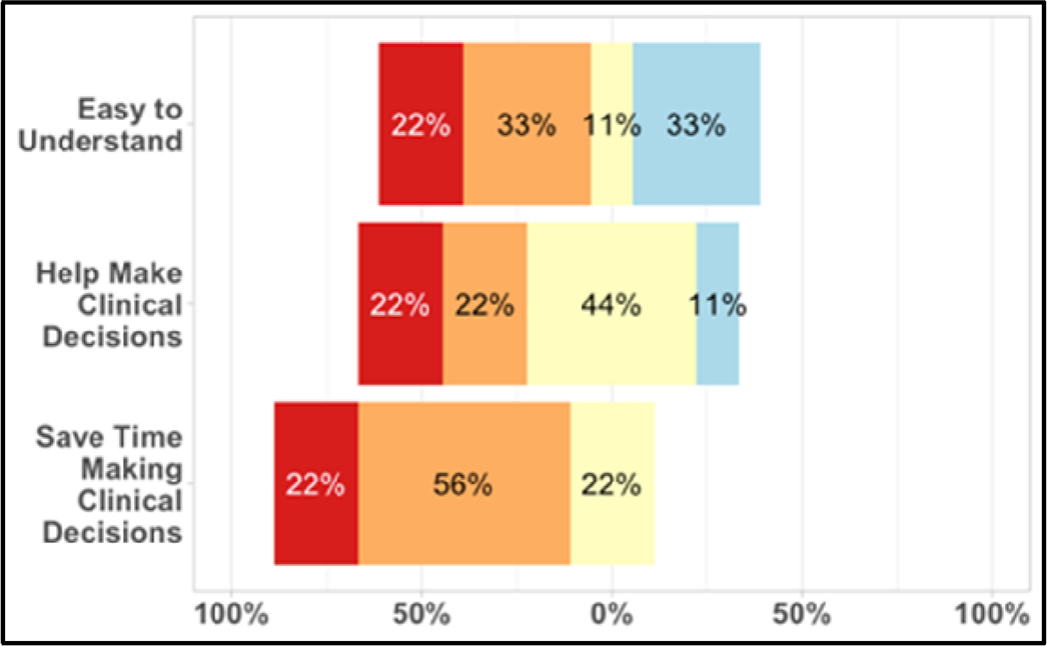	• Like the concept; helpful information
			• Wish it was easier to interpret; prefer a line graph
Data Visualization—#6			
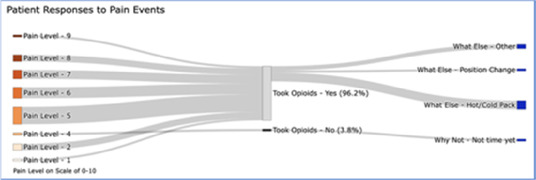	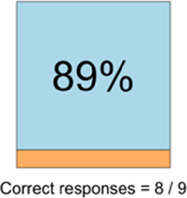	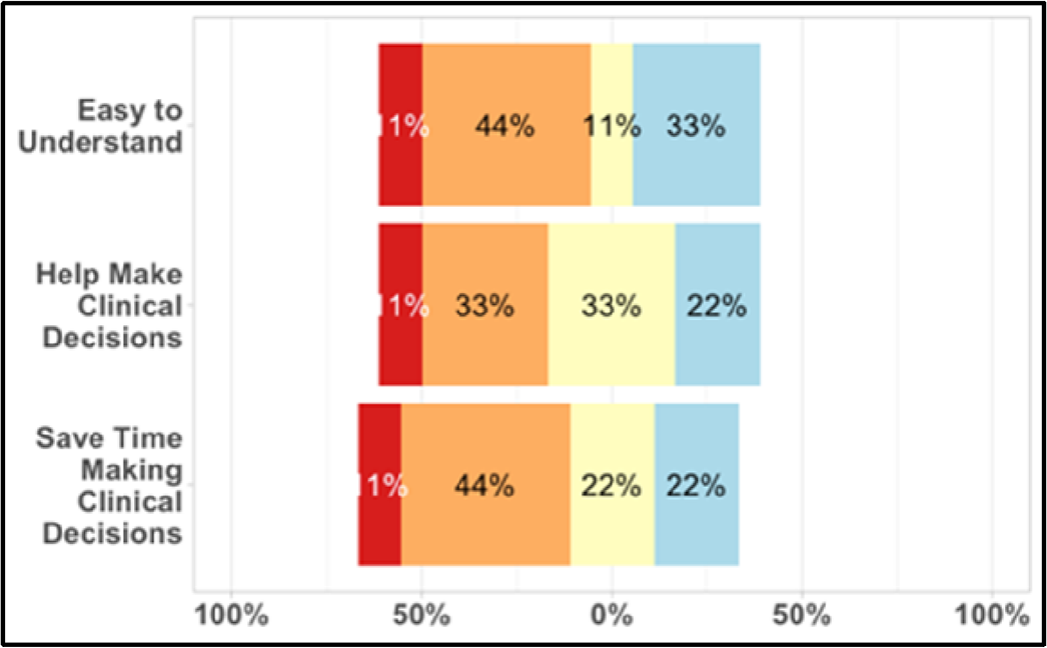	• Valuable information
			• Wish it was same as the bar graph, a day-by-day report

**Table 5:** Feedback session 3—Academic Medical Center.

A: Data Visualization	B: Comprehension Question	C: Quantitative Feedback	D: Qualitative Feedback
	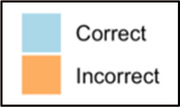	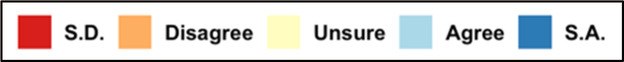 S.D. = Strongly Disagree S.A. = Strongly Agree	What do you find helpful?/ What do you like?
			What would you change?/What is missing?/What questions do you have?
Data Visualization—#1			
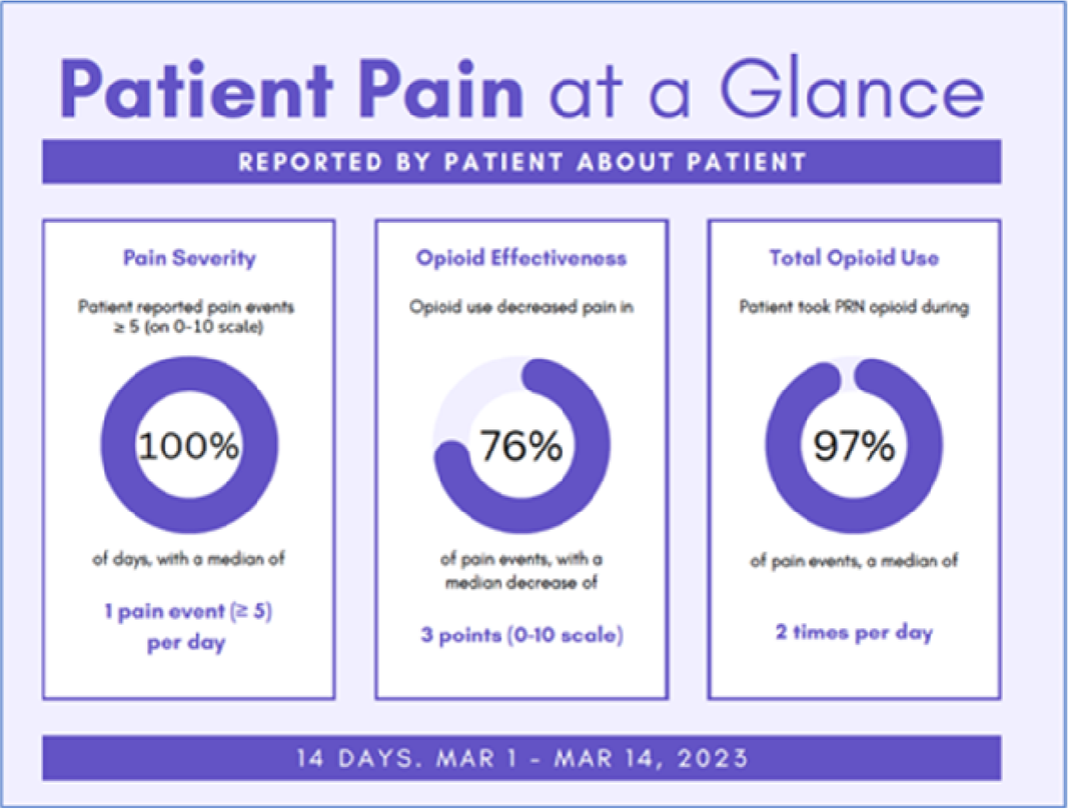	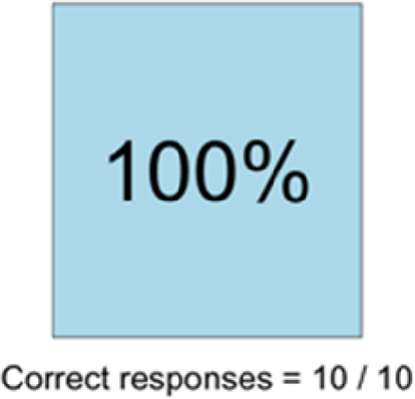	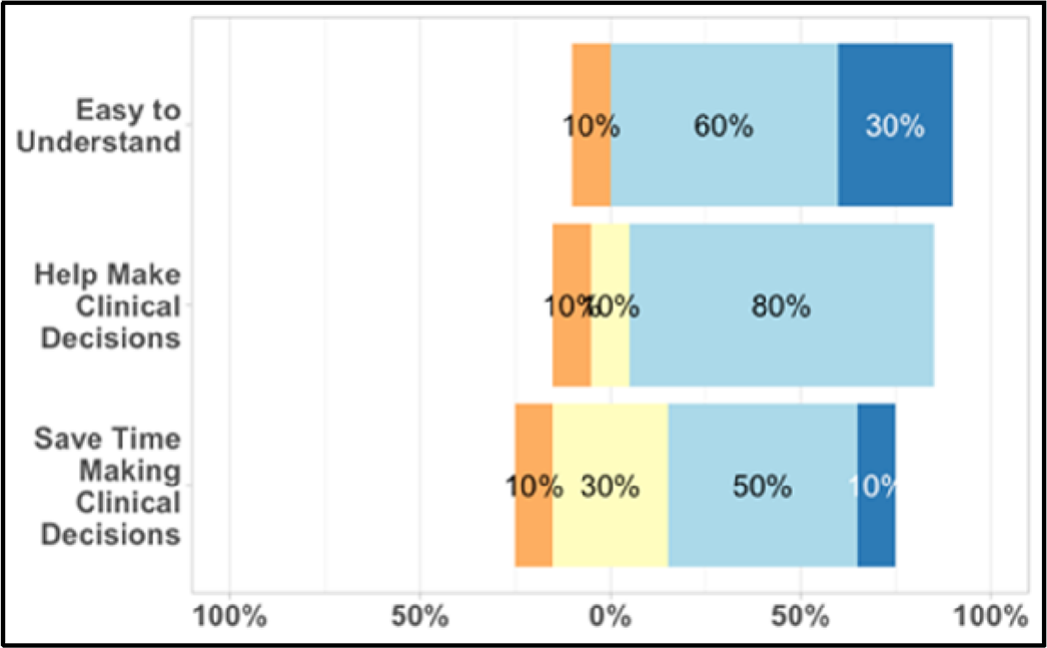	• Like the information at the bottom (in purple) • Easier to read than previous iterations • Like that it's simple
			• Would like to see the donut and purple text flipped vertically • Titles not quite right; wording unclear
Data Visualization—#2			
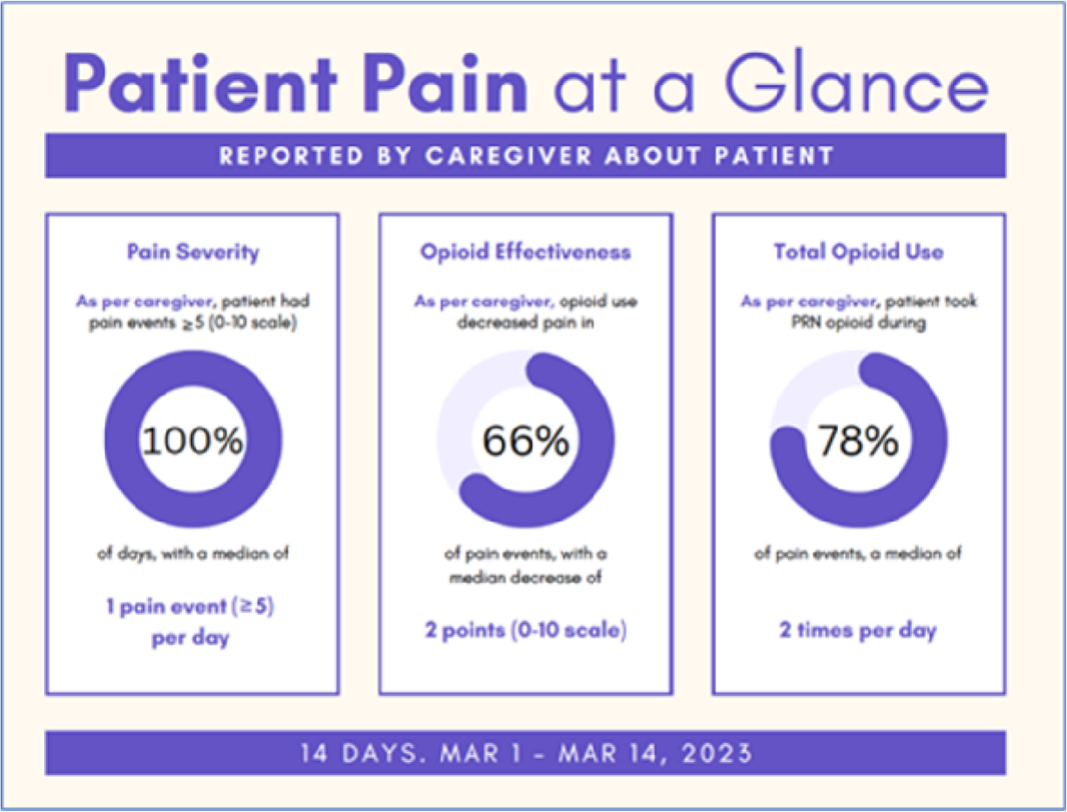	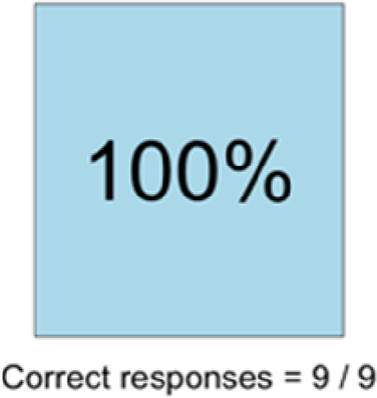	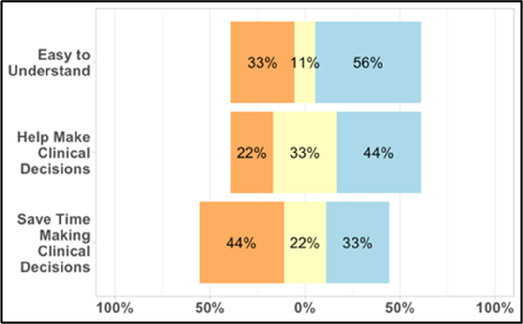	• Great for hospice patients who can’t communicate • Having patient and caregiver data all in one panel would be overwhelming; like that it's split into two visualizations.
			• Needs to be clearer that this is information reported by caregiver about the patient • Would like caregiver data side by side with patient data • Would like context for how much time patient and caregiver are spending together
Data Visualization—#3			
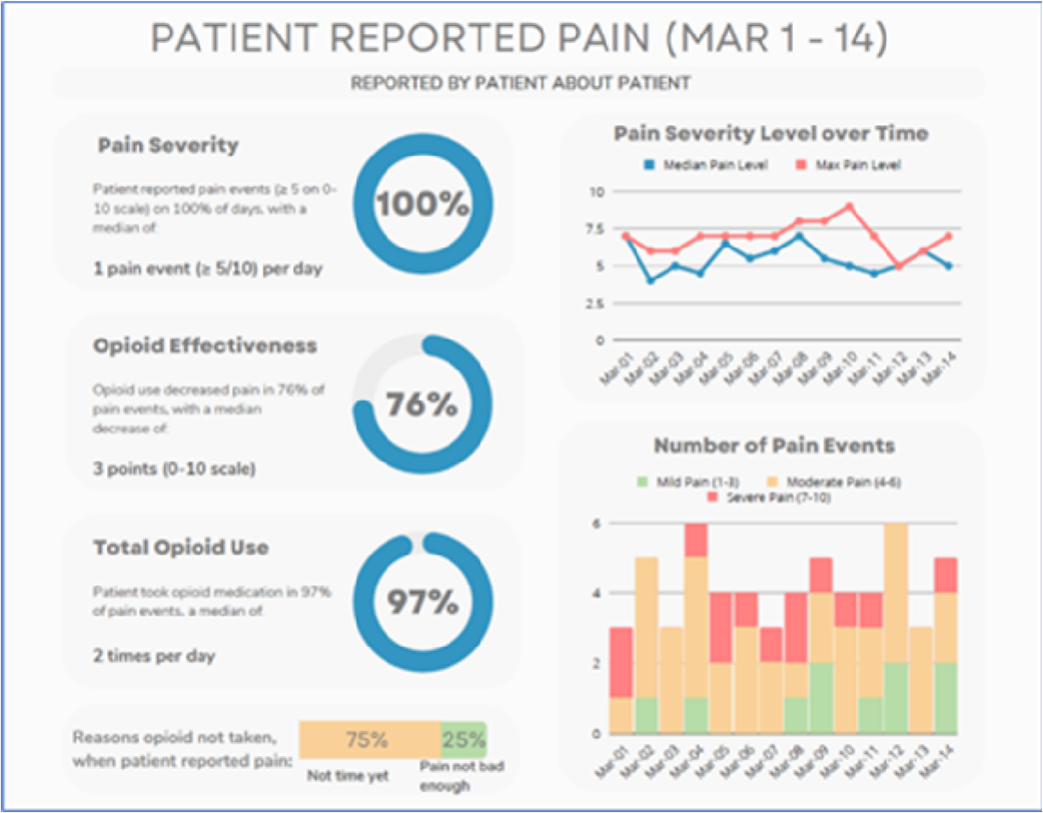	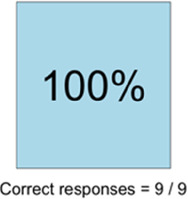	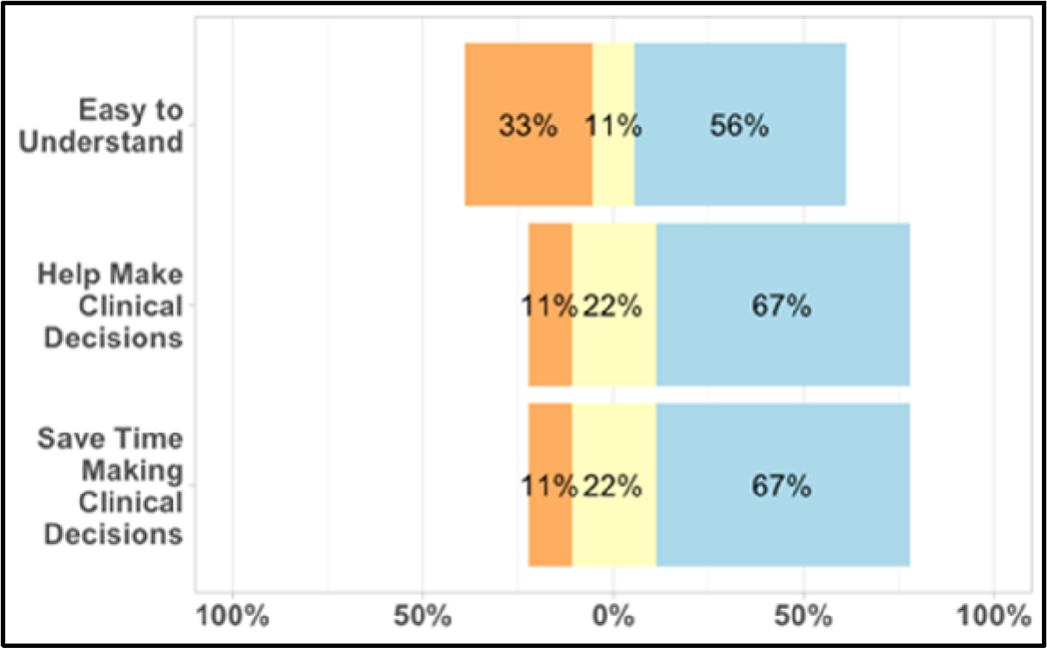	• Like the max and median pain level plot • Like seeing reasons pain medication not taken
			• Not interested in mild pain events • I like the graphs much better than the percent circles; they provide me with more data points to extrapolate from and aid in better clinical decision making
Data Visualization—#4			
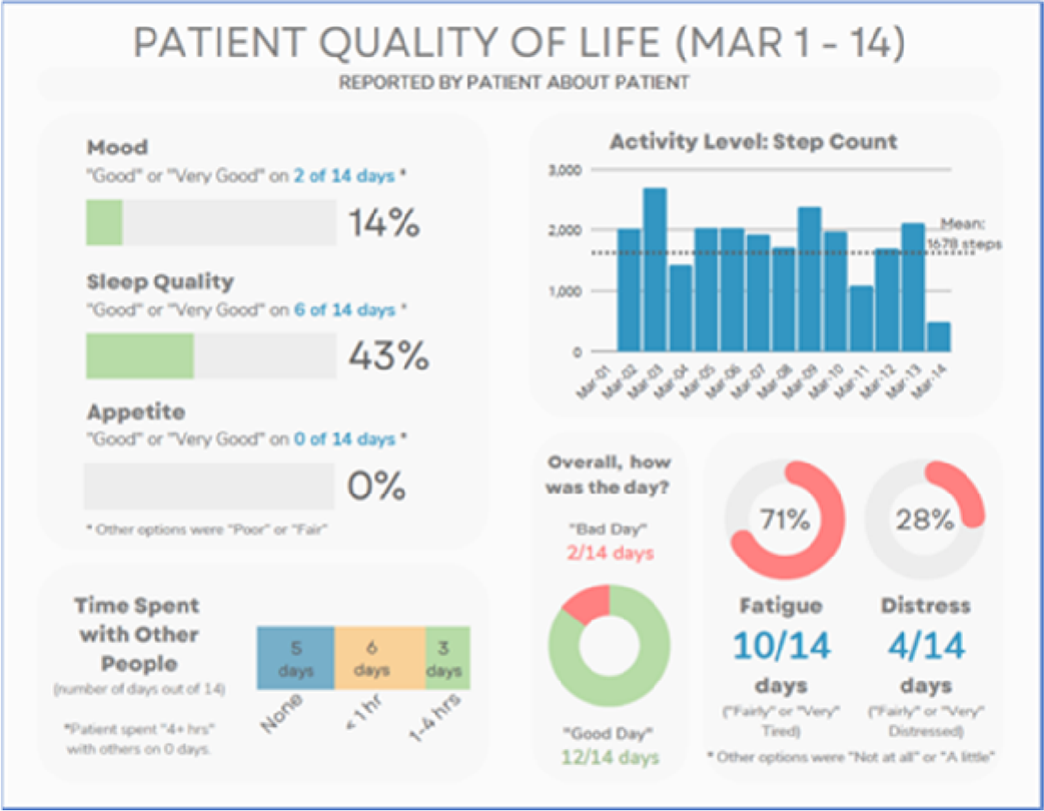	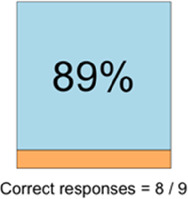	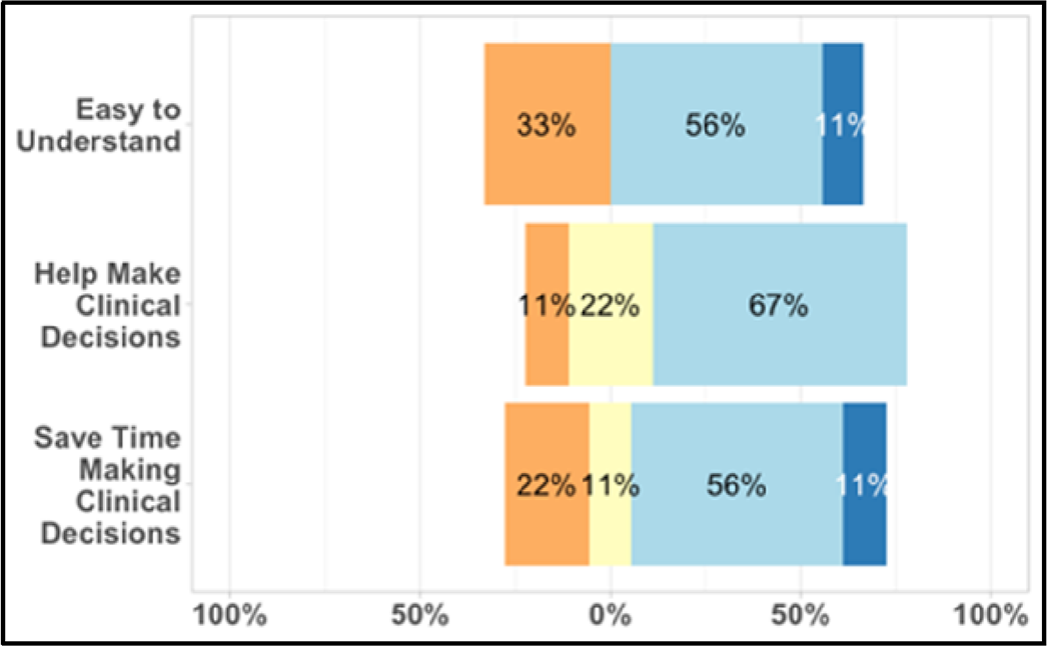	• Really like the quality-of-life information • Number of days is preferred over percentage
			• Having both percentage and number of days is redundant • It's a lot to process and seems to present conflicting data that would require more information gathering (e.g., how is mood so bad but they aren’t having more bad days?) • I like the step count, but would there be an activity metric for chair bound people
Data Visualization—#5			
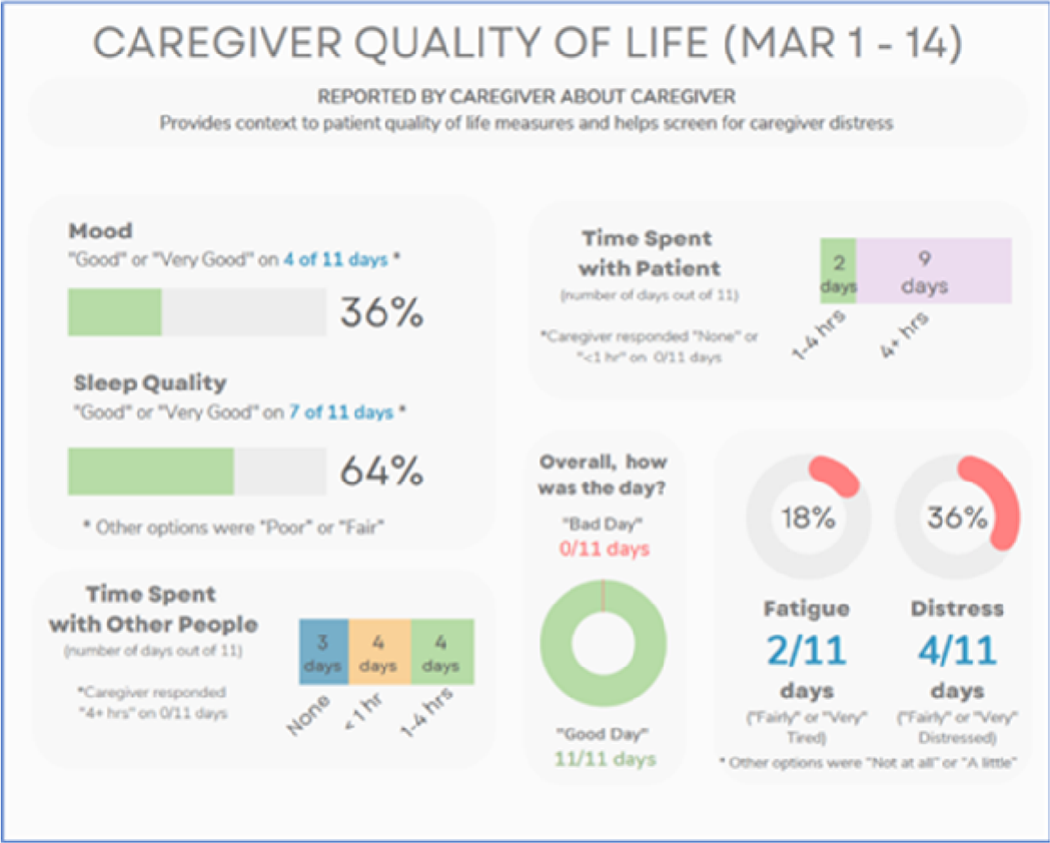	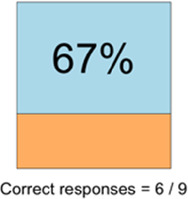	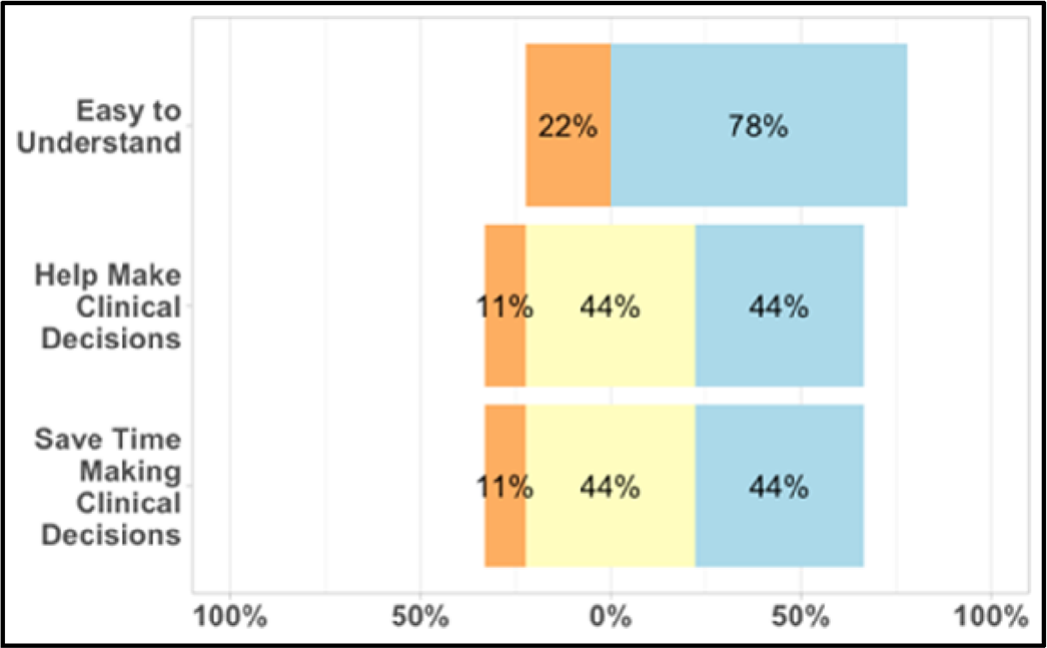	• Like seeing the “time spent with other people” data
			• Helpful to only see negative for green bars, because could be seeing “fair” as an acceptable response • Need to highlight that this is “caregiver about caregiver” data

**Table 6:** Feedback session 4—Community Hospital.

A: Data Visualization	B: Comprehension Question	C: Quantitative Feedback	D: Qualitative Feedback
	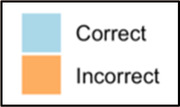	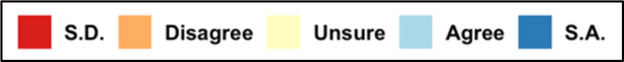 S.D. = Strongly Disagree S.A. = Strongly Agree	What do you find helpful?/ What do you like?
			What would you change?/What is missing?/What questions do you have?
Data Visualization—#1			
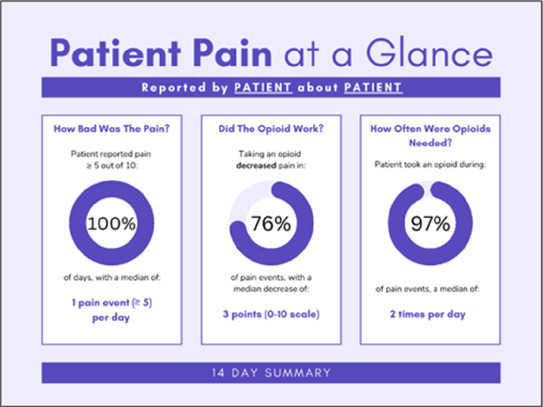	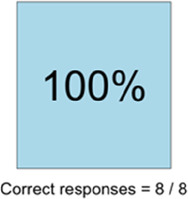	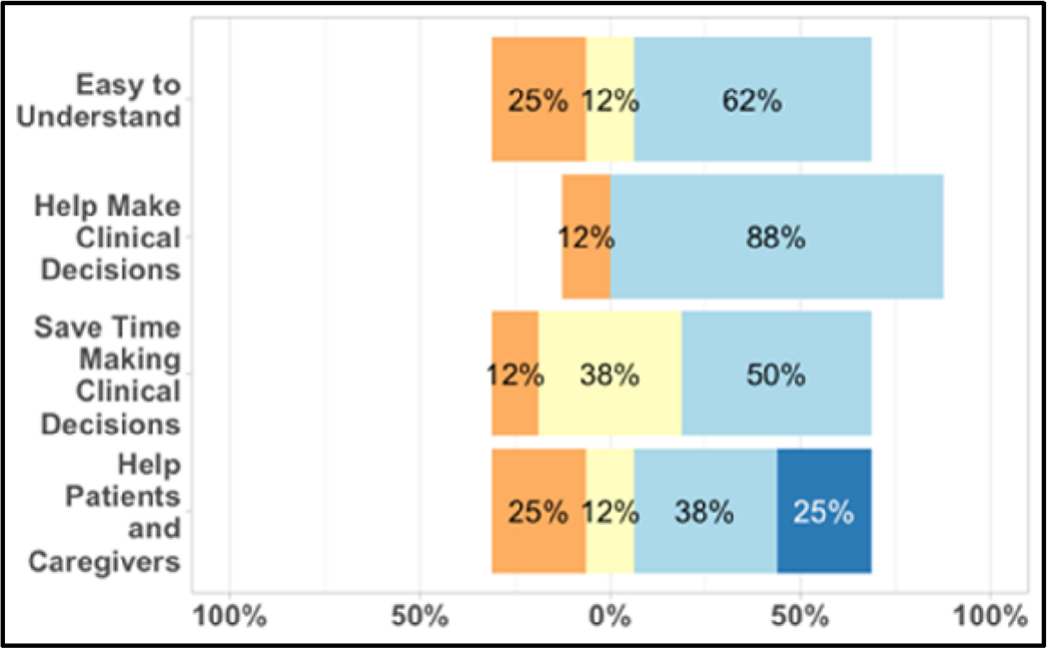	• Like it better, understand it better after seeing it a couple of times
			• Greater than symbol should be written out (patients wouldn’t understand) • Put “Patient Reported” in larger font so it's clearer where data is coming from • Want to know about severe pain (≥8/10) • First rectangle took a minute to figure out • Too many words • Break up the first box into two separate boxes
Data Visualization—#2			
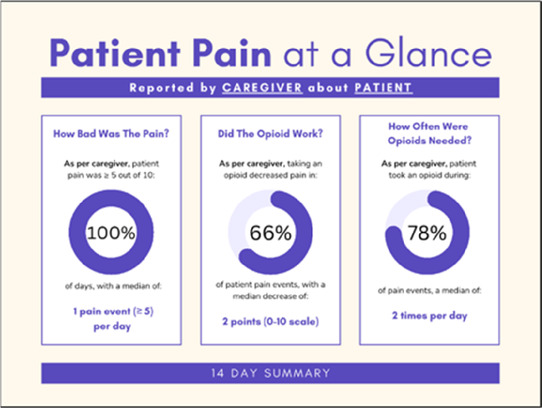	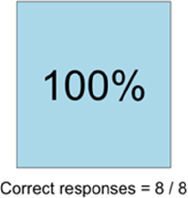	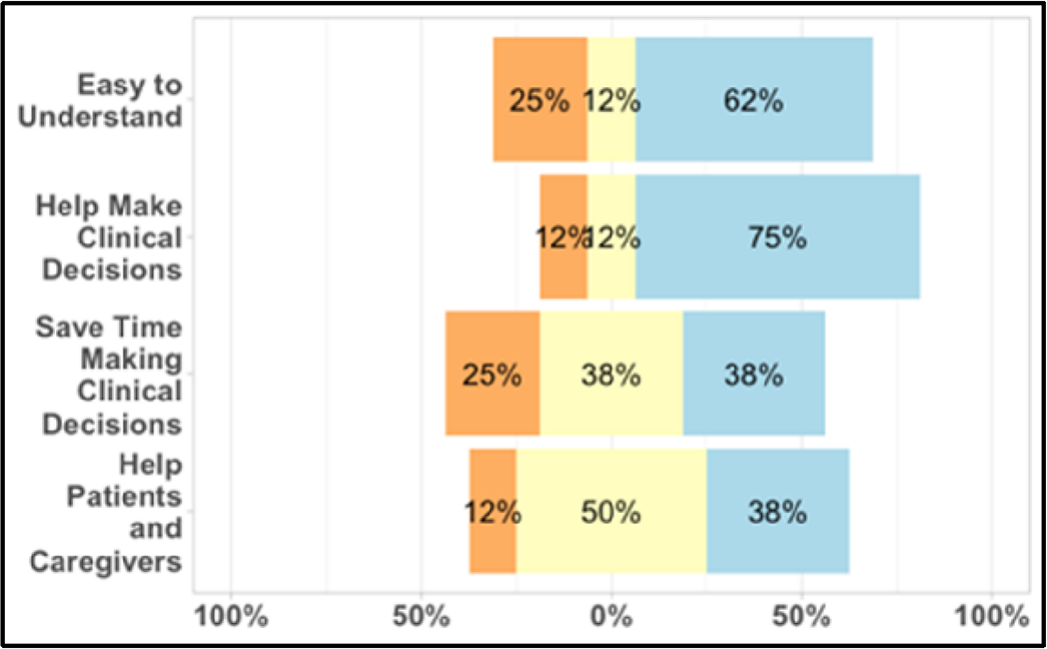	• Interested in the difference between patient and caregiver, maybe put data side by side or overlapping
			• Bottom numbers are more relevant vs. the percentage values
Data Visualization—#3			
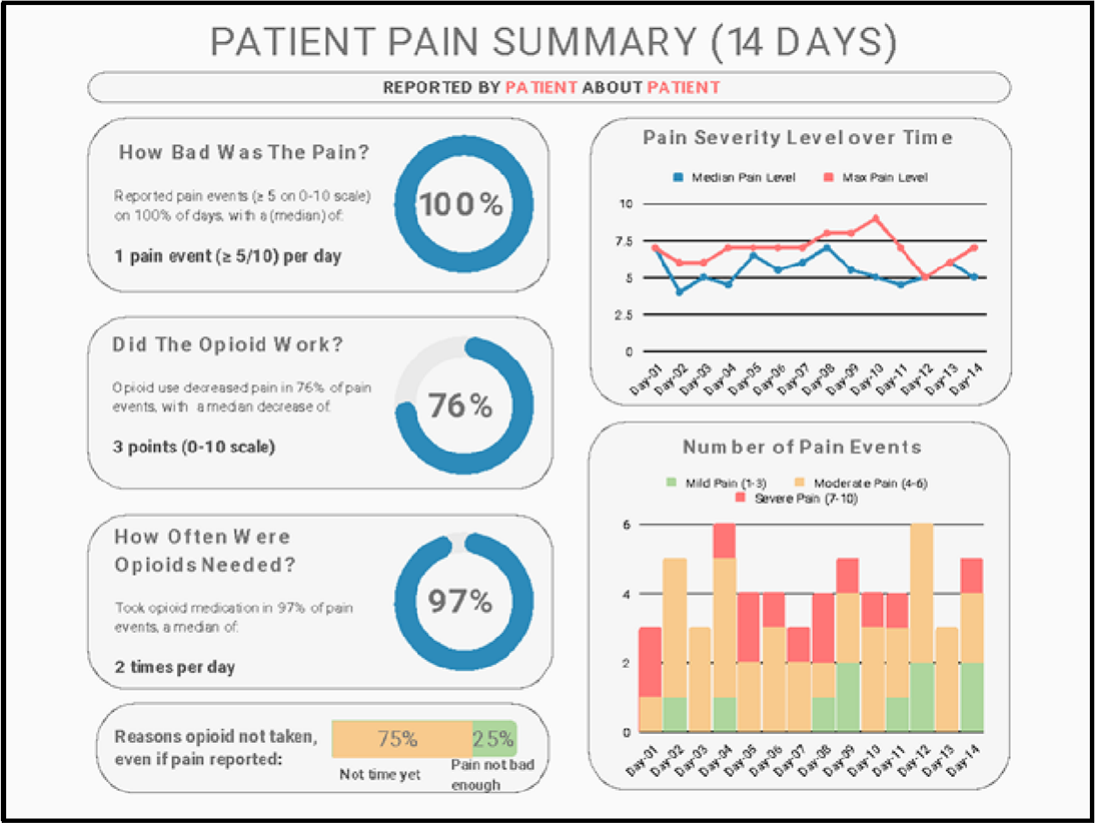	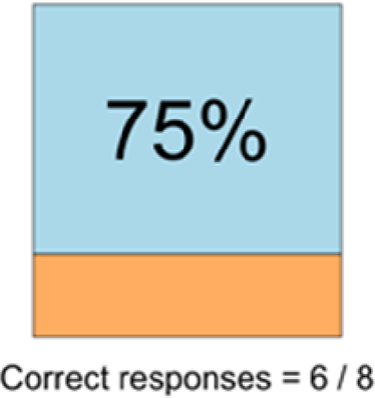	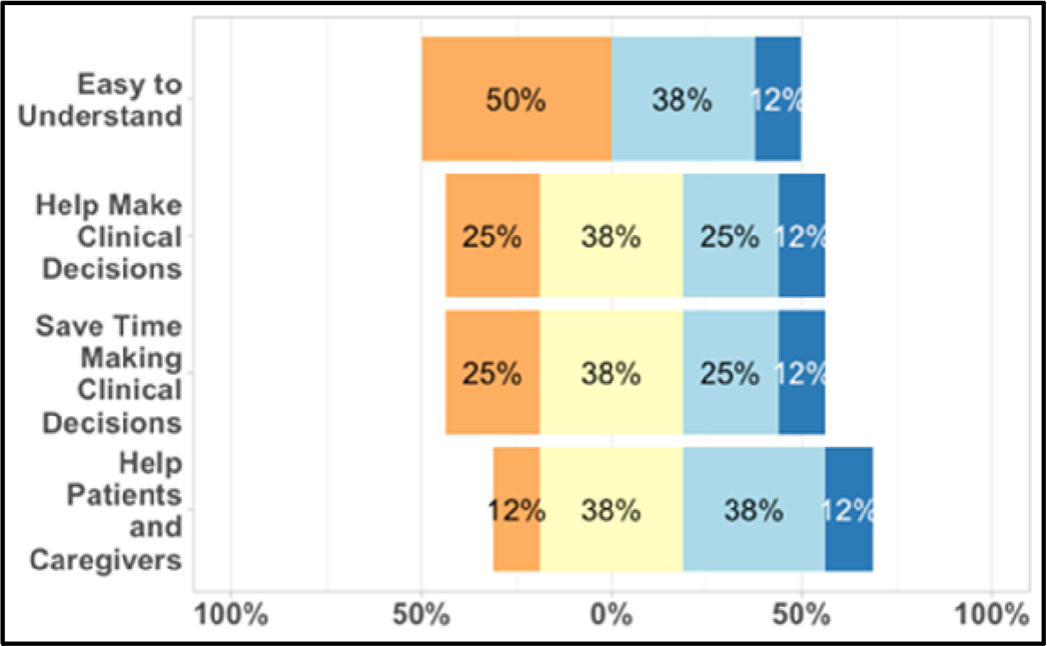	• Like this better than the other visualizations • Per day breakdown is better than the summary totals • Pain event bar chart would be helpful in talking with patients • Liked seeing reasons opioid not taken
			• Vertical axis needs label • Interactive would be nice so you can hover over to get additional information • Flip bar chart to the side to help with understanding that it's a count
Data Visualization—#4			
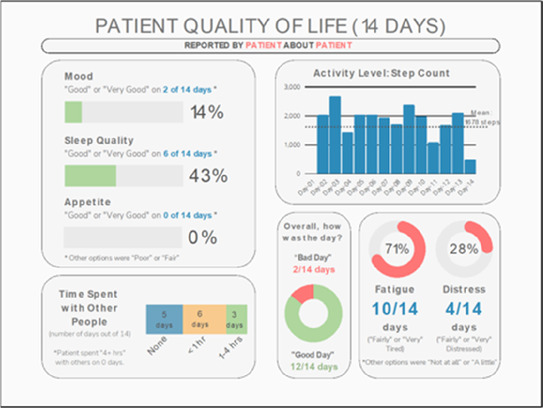	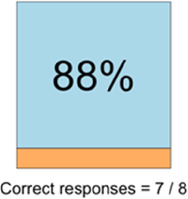	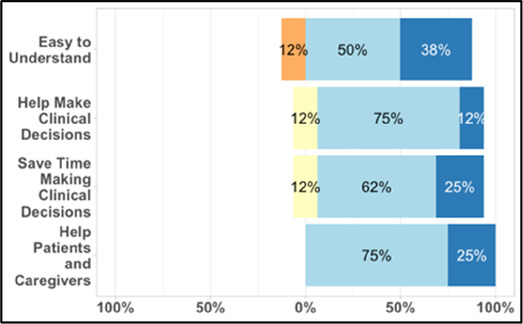	• Like that it gives number of days and also percent • Really like the quality-of-life data—would like to share with patients and families
			• Like to know trend in step count, to see if the person is in decline • Would like problematic data to be flagged in some way, for example when “zero appetite” recorded
Data Visualization—#5			
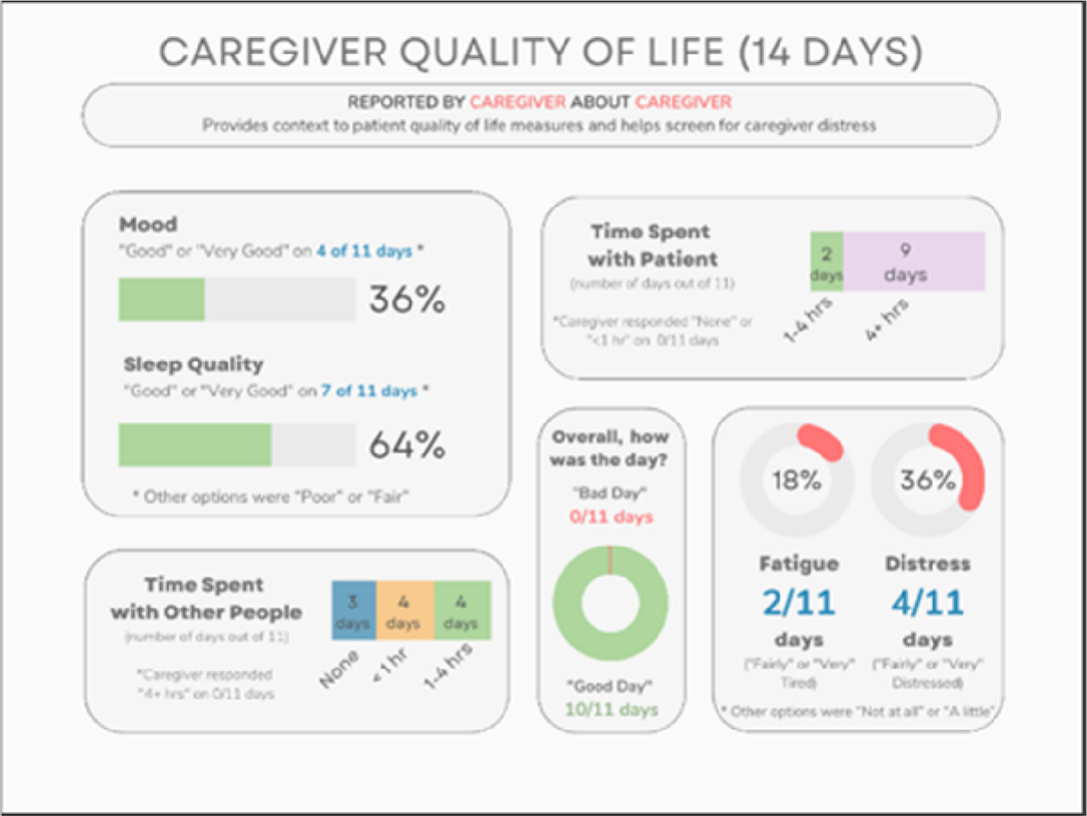	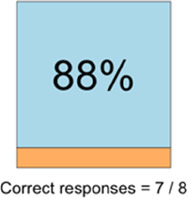	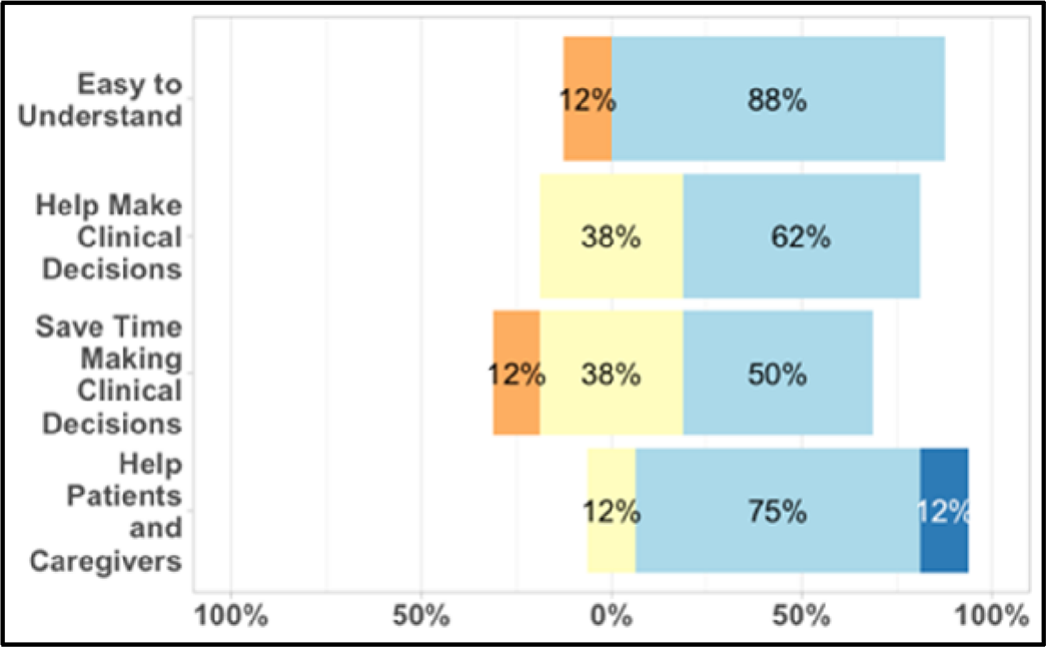	• Not currently getting information on caregivers, really helpful context (noted especially from social worker)Like the information about time spent with others • Interested in difference between caregiver's perception of themselves vs. patient's perception (e.g., am I a burden?)
			• Are you feeling overwhelmed?’ is a better question for caregiver as a flag for burnout and/or potential elder abuse; more likely to admit to being overwhelmed vs. “distressed”
Data Visualization—#6			
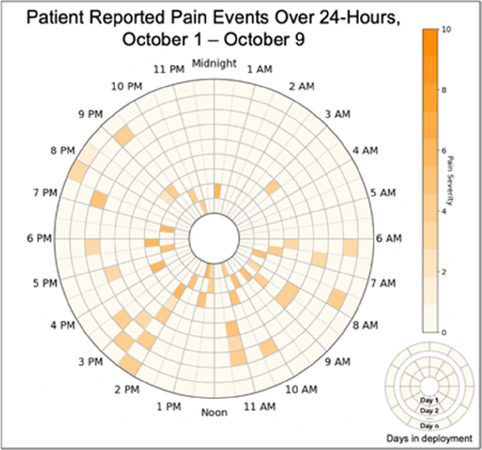	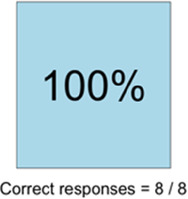	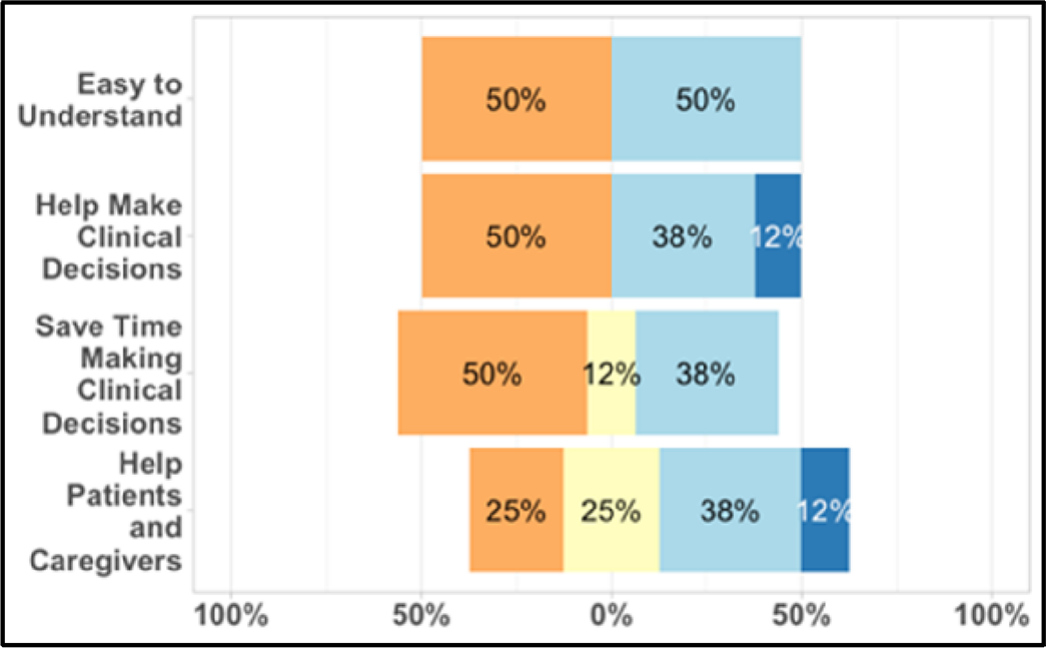	• Once I figured it out, really liked it • Helpful to know the times that they were in the most pain • Love it! Time saver! • With explanation, helpful for patients and caregivers, for example could let families know what times to hire help
			• Hard to see the gradation with color palette

**Table 7:** Feedback session 5—Hospice.

A: Data Visualization	B: Comprehension Question	C: Quantitative Feedback	D: Qualitative Feedback
	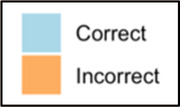	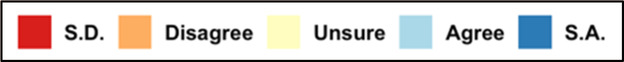 S.D. = Strongly Disagree S.A. = Strongly Agree	What do you find helpful?/ What do you like?
			What would you change?/What is missing?/What questions do you have?
Data Visualization—#1			
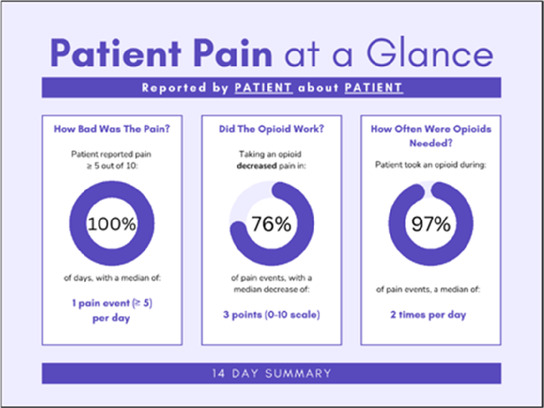	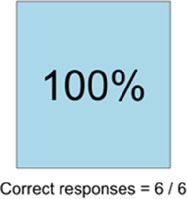	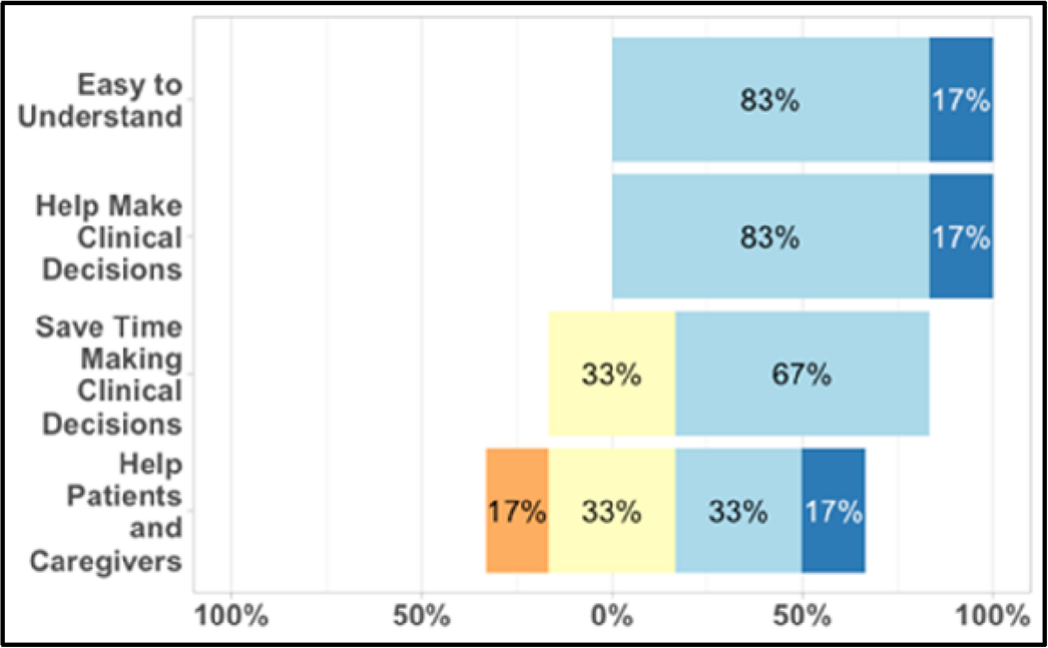	• No responses
			• Took a couple of minutes to read and understand;, especially what percentages represented • Generated more questions than answers: ○ Are they taking their long acting or short acting opioid? ○ When is the opioid working and when is it not working? If pain is decreasing, by how much is it decreasing? What was the before and after pain level? • Would be helpful to be able to see what medications were taken and not have to cross interface with the EMR or something else
Data Visualization—#2			
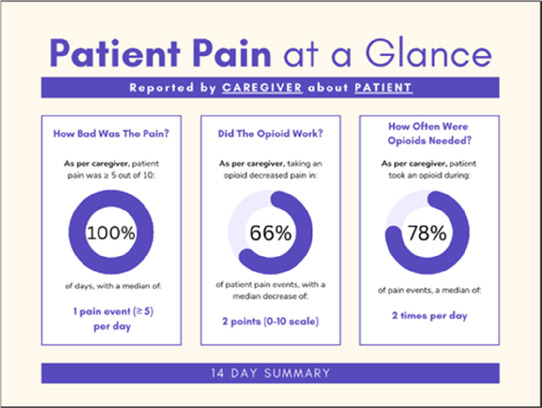	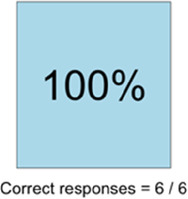	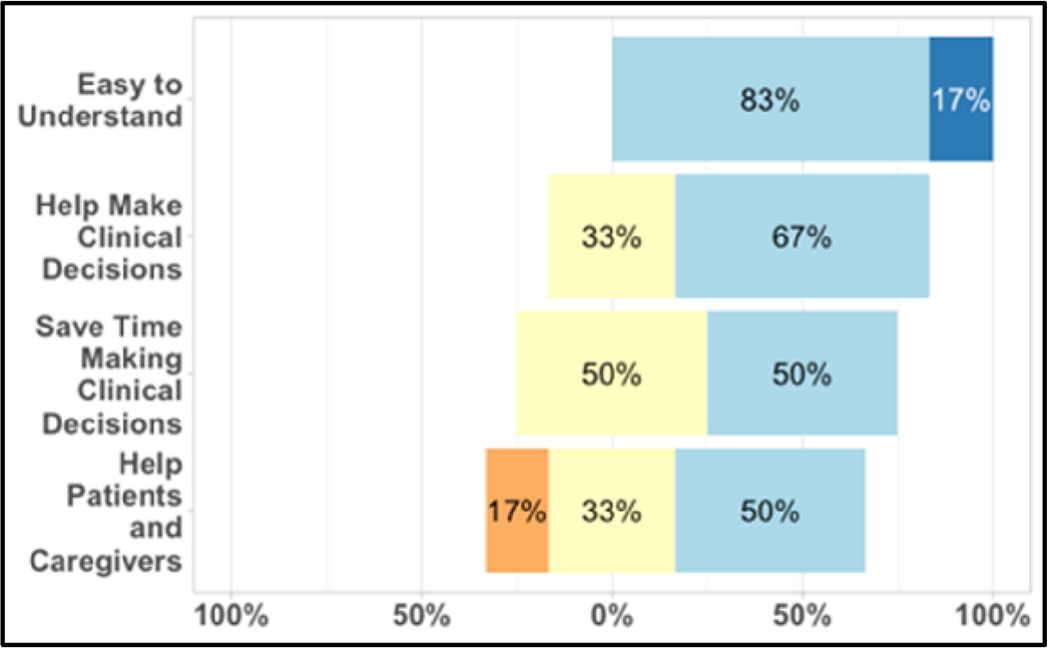	• Strong interest in understanding the caregiver's perspective; their advocacy for the patient is crucial, especially as patient becomes more ill and cannot speak for themselves
			• Including the patient's personal pain goal is important as range for acceptable pain is different for patients, for some, being at a 5 or 6/10 is tolerable and acceptable
Data Visualization—#3			
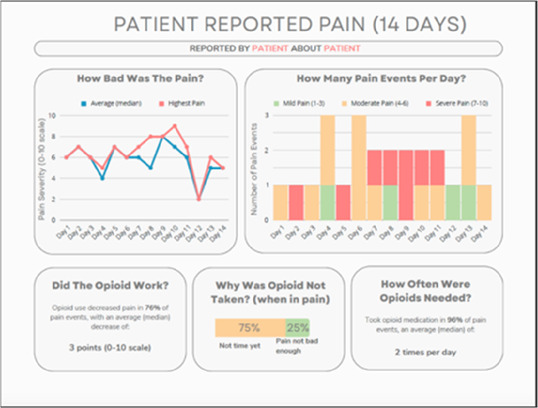	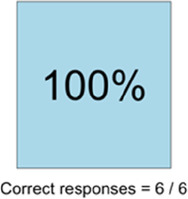	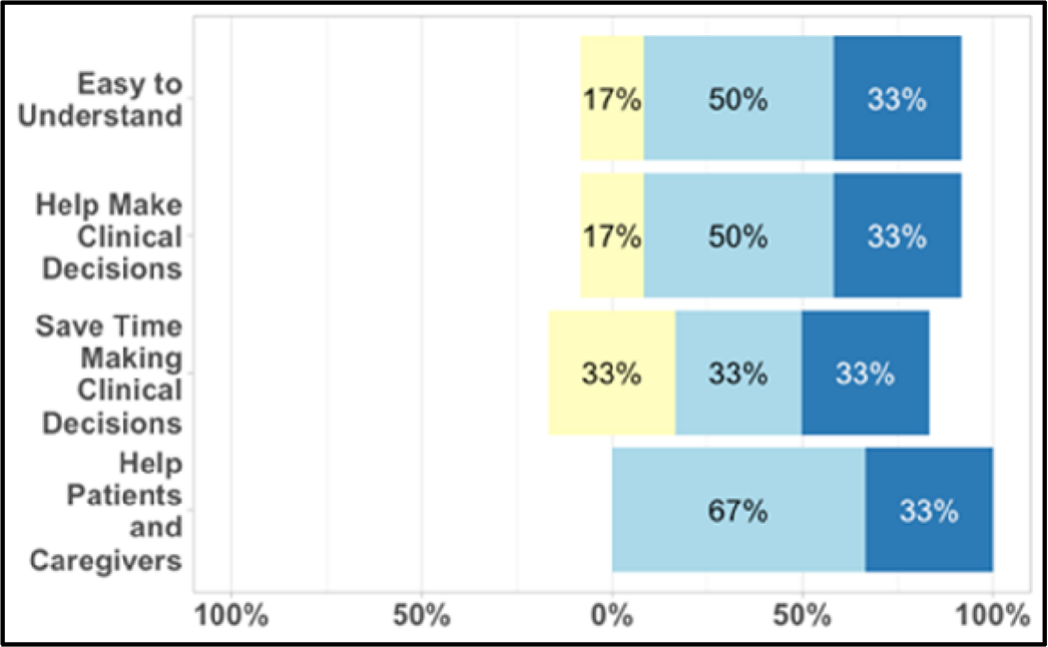	• The average in highs and the differences each day is very helpful; provides a better picture of the patient's pain experience • Useful to know the context of the days and timeline for pain events; it is helpful to know why patient took an opioid • Like this better than visualization #1; easier to understand • Would be really helpful to share with patients, to see trends over time; data also helpful for nurses communicating with doctors before writing orders • We ask for pain/medication logs which can be challenging for patients to record, this would help make it easier
			• Would like to know “n” for why opioid not taken and understand the context for the percentages of why an opioid was not taken • Took a minute to figure out the bar chart of number of pain events; maybe segment colors in bar chart with black border or write “low” “medium” “high” in text on the bars • Recommend a colorblind friendly palette; if on a dashboard option to toggle to colorblind friendly version
Data Visualization—#4			
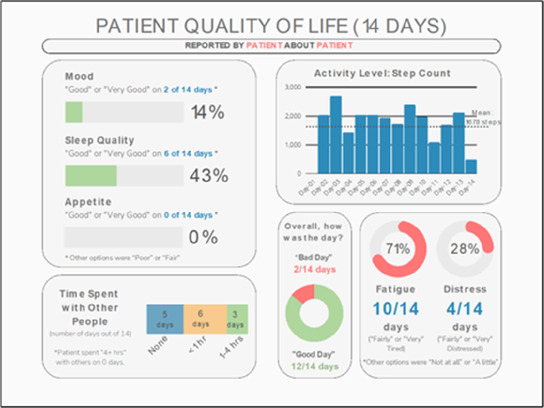	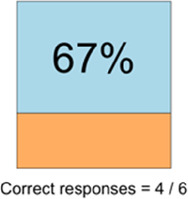	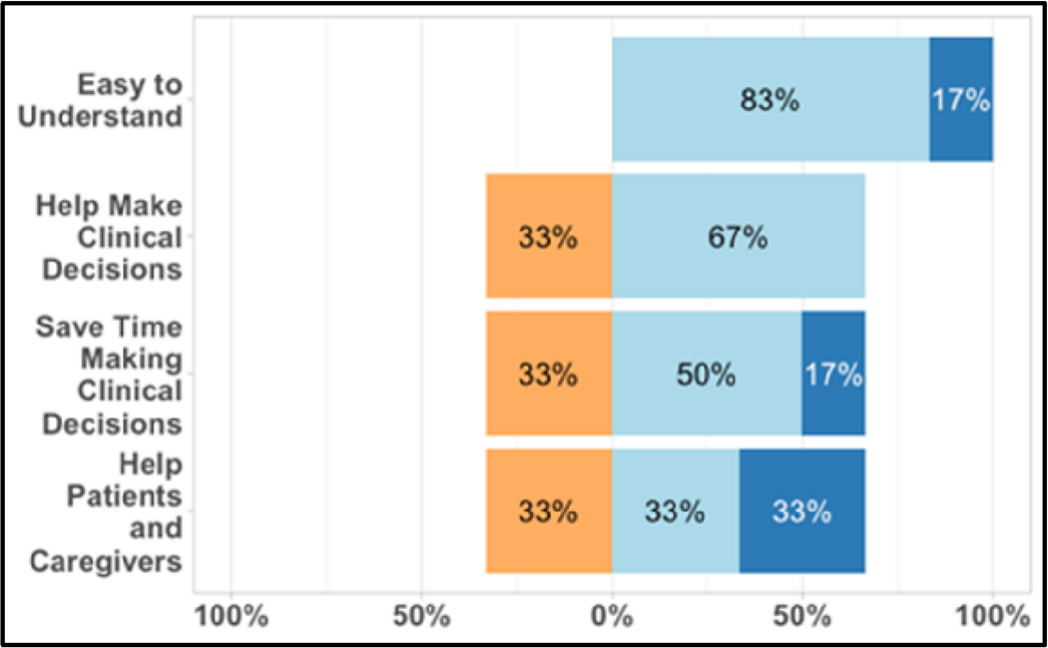	• Love the “good days” and “bad days”
			• Some of the quality-of-life measures should not be shared with patients and/or caregivers; we may not want to draw attention to things like decreasing appetite or activity because we expect those things to decline in the end of life. Instead, we want to shift caregiver attention onto other things • Have a dashboard of all of the patients so a clinician can compare across patients, e.g., “who is the most anxious?” and prioritize accordingly • Correlate the quality-of-life measures with the pain events; identify contributing factors to worse days
Data Visualization—#5			
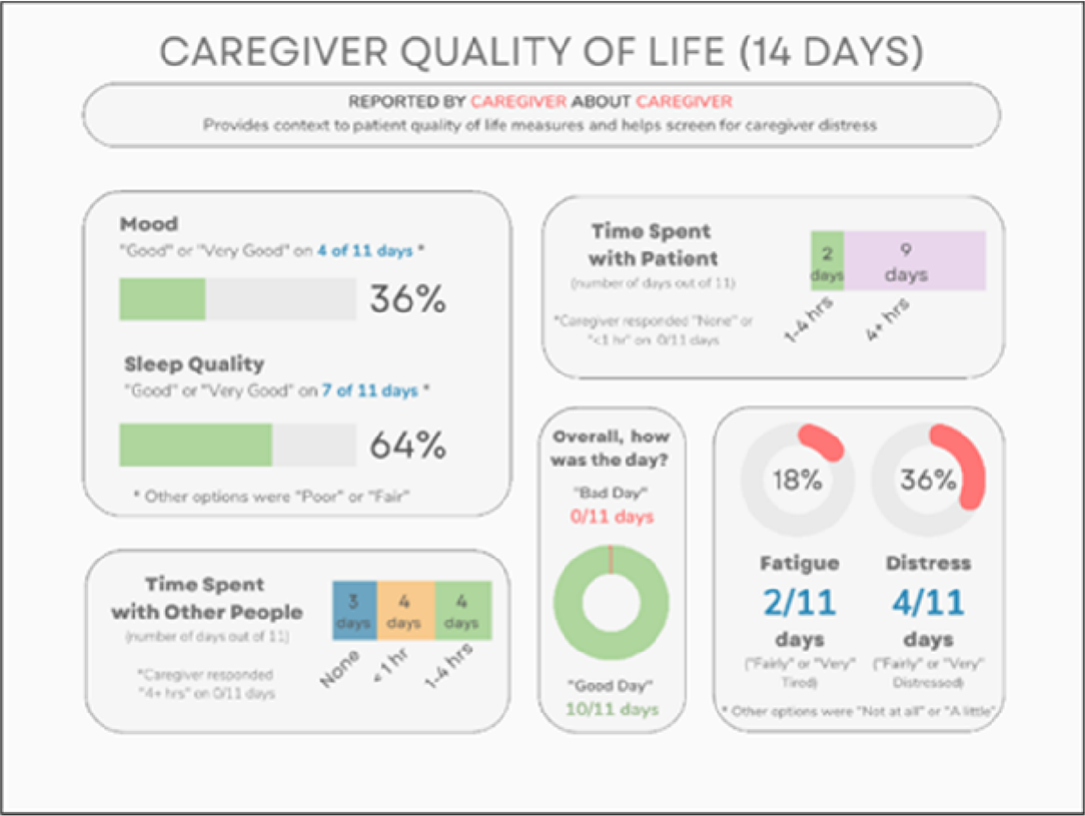	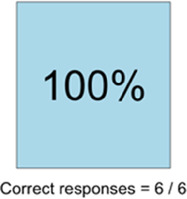	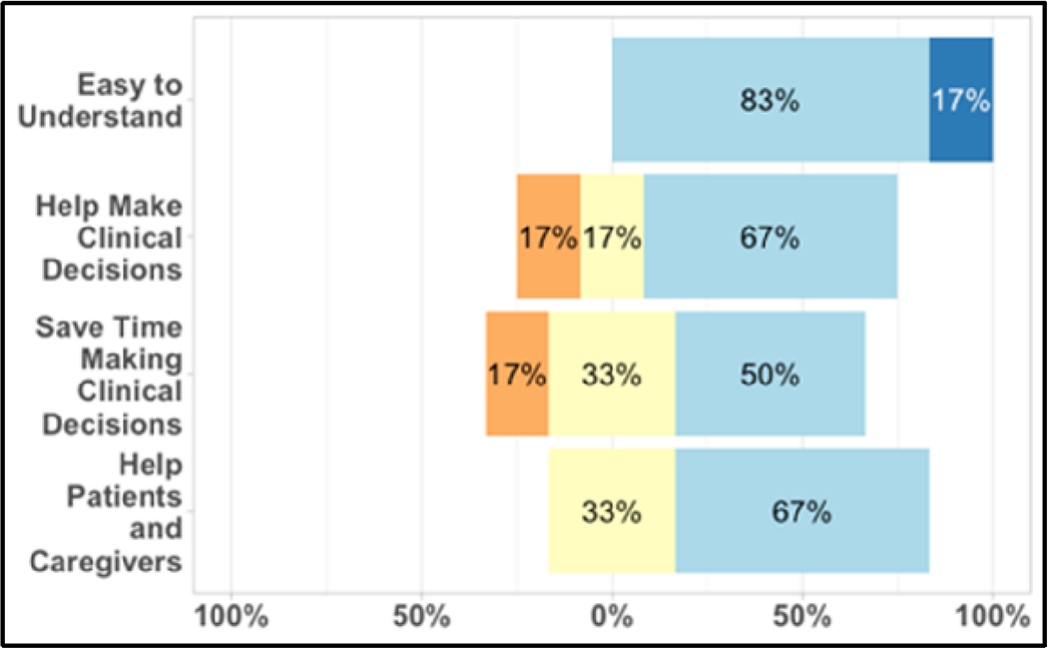	• The caregiver may be more likely to express this information through the BESI-C system rather than vocalizing it in clinic • Could be a good conversation starter; families may not volunteer this information when struggling • Great for people who may be able to give more focus to the caregiver as part of their role—like a chaplain or social worker
			• More story and context would be important to understand how this could be applicable: which caregiver is this? How involved are they with the patient? Is anyone else involved? • For a physician, this may be too much information, but there may be team members that could better utilize this context (e.g., doulas, chaplains, social work) • Difficult to know what might be actionable from this information about the caregiver • Felt this was crossing into an overwhelming amount of information • Would like to compare day by day with patient information (e.g., is the caregiver having bad days on patient's bad pain days)
Data Visualization—#6			
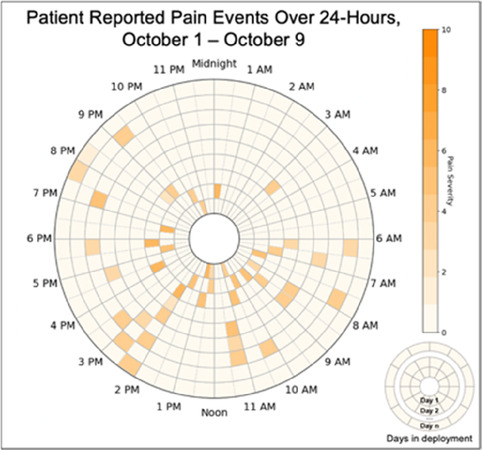	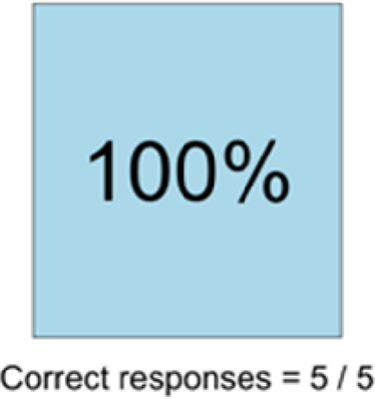	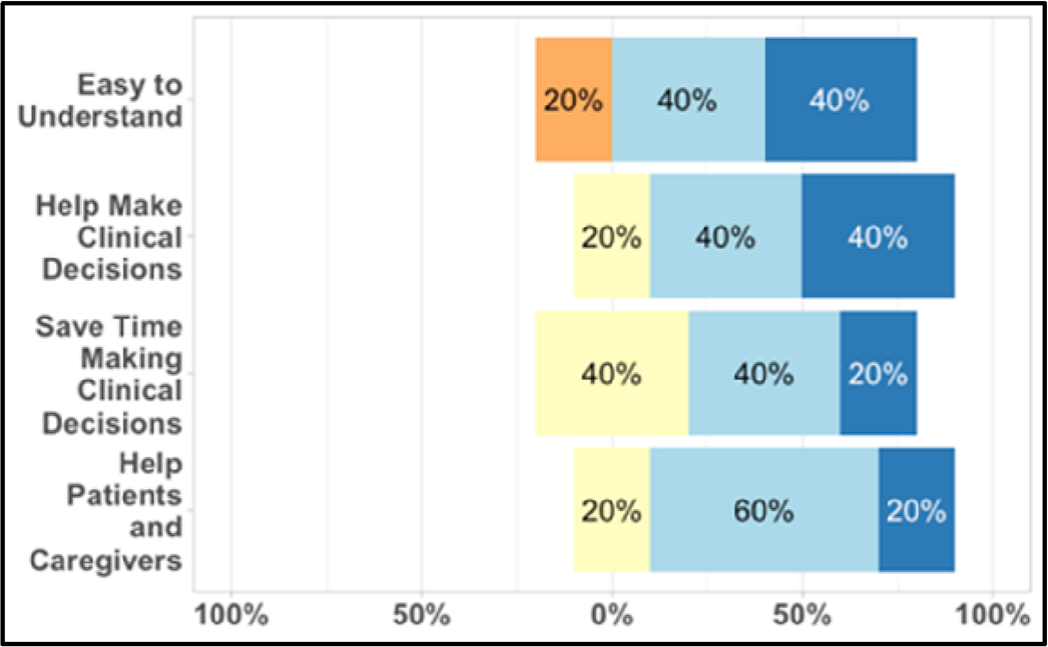	• This is really cool, like this a lot! • Quickly and clearly paints a picture of the patient's pain situation • Took a second to figure out, but liked it after it was clear • Gives a nice perspective of pain events over time
			• Would need an orientation to explain to everyone how this works, but very helpful once understood • Missed the legend entirely; should make the color contrast more noticeable; • Missed the severity piece—colors are too similar to distinguish between different levels of pain events • By having all the times where pain events aren’t being recorded as set to 0, creates assumption that the patient wasn’t having any pain at that time, which is likely not true—suggestion to grey out blocks where no pain was being read; need to distinguish between not reporting pain vs. reporting no pain • Would be great to overlay with other information, where you hover over a tile and can see more context about pain events, when they took pain medication, etc. • Would pair well with activity information; could help educate patient about strategies for pacing, pre-medicating before activities, etc.

#### Data visualization viewing preferences

Regarding overall data viewing and sharing preferences, (survey items answered by *n* = 24 participants in sessions 3, 4, and 5; these items were not asked in sessions 1 and 2, see Supplementary Data 2), the majority (62%; *n* = 15) said they would like to have both options—an interactive dashboard and a static document (such as a PDF attached to the patient record)—to view patient and caregiver reported symptom data; 38% (*n* = 9) said they would prefer only an interactive dashboard where they can change parameters and customize the data they see; no (0%) of participants expressed a preference for viewing data visualizations only as a static document. When asked, “how important is it to you that patient/caregiver reported symptom data are integrated within the electronic health record?,” 92% (*n* = 22) of participants reported it was “important” (*n* = 10; 42%) or “extremely important” (*n* = 12; 50%); 1 participant (*n* = 1; 4%) reported it was “a little important” and 1 participant (*n* = 1; 4%) reported it was “not at all important.”

#### Data visualization content priorities

**Figure 4:**
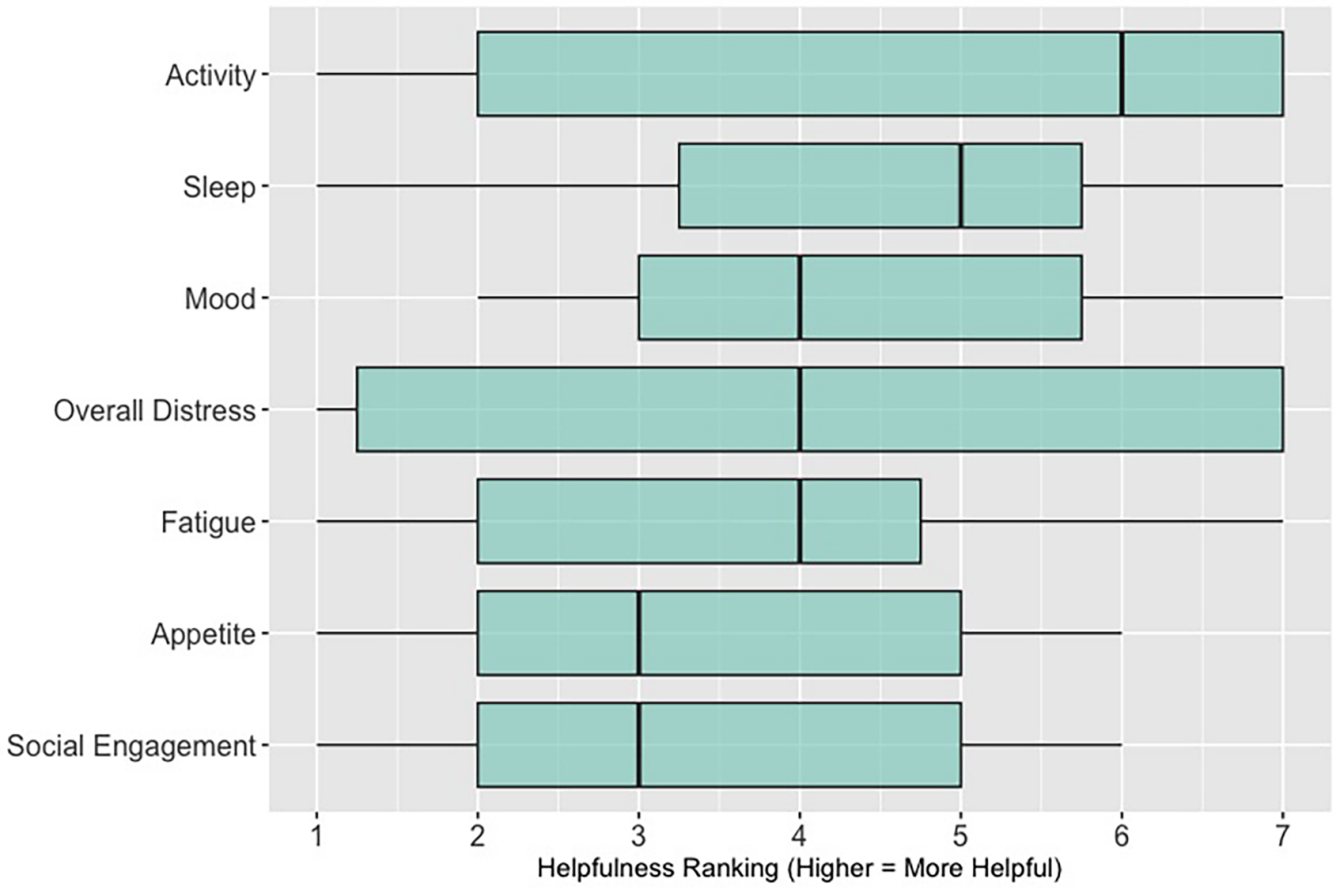
Clinician rankings related to perceived helpfulness of patient quality-of-life data.

**Figure 5:**
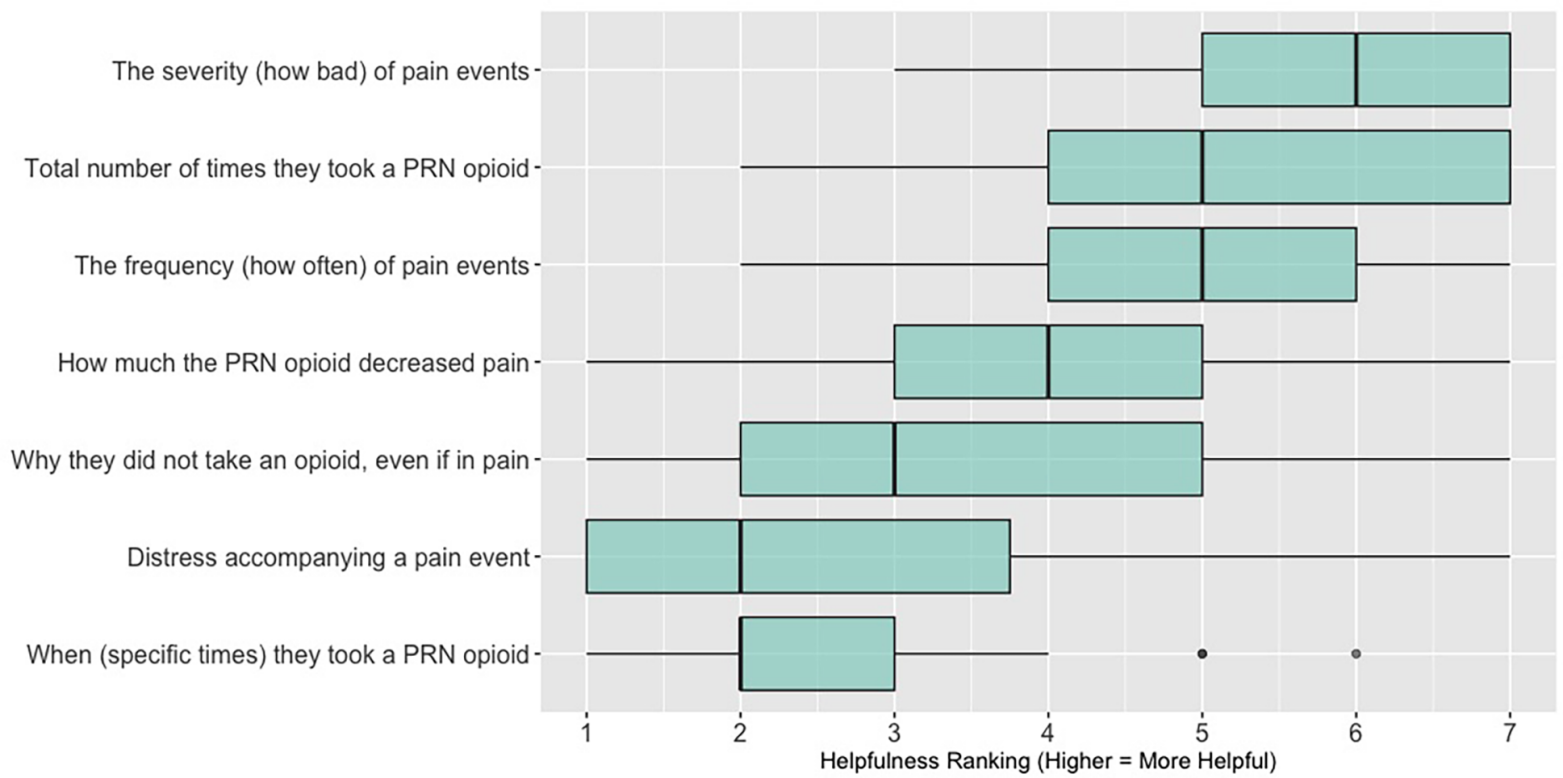
Clinician rankings related to perceived helpfulness of patient pain assessment data. PRN, as needed.

When asked to rank which quality of life data (out of 7 options: activity; sleep; mood; overall distress; fatigue; appetite; social engagement) are most helpful when assessing a patient (survey items answered by *n* = 26 participants in sessions 3, 4, and 5; these items were not asked in sessions 1 and 2, see Supplementary Data 2), 62% (*n* = 16) of participants ranked “sleep”; 58% (*n* = 15) ranked “activity” and 46% (*n* = 12) ranked “mood” in the top three spots. Figure 4 shows these results as a box plot, with “activity” receiving the highest average helpfulness ranking. When asked to rank which pain-related information is most helpful when assessing a patient, 85% (*n* = 22) of participants ranked “severity of pain events”; 62% (*n* = 16) ranked “frequency of pain events” and 58% (*n* = 15) ranked “number of times they took a PRN opioid” in the top three spots. Figure 5 shows these results as a box plot, with “severity of pain events” receiving the highest average helpfulness ranking.

### Qualitative results

Qualitative feedback from the discussion portions of each feedback session (along with the write-in free text survey responses) are summarized for each feedback session, per visualization, in Tables 3–7, column D. While each feedback session provided granular comments specific to particular visualizations, there were also overarching themes that emerged across all discussions and applied to multiple visualizations; these themes are summarized below.

### Key themes related to discussions of data visualizations

#### Desire for simplicity

There was a strong preference for simplicity and ability to quickly grasp the core take-home message for each data visualization. As one participant in session 4 succinctly stated, “less words, and more pictures!” Participants often volunteered their self-perceived low data literacy related to being able to interpret data visualizations. However, appreciation for the value of less familiar visualizations, such as the circular plot pain wheel, usually improved after a brief explanation (e.g., “after it was explained, I really like it!”).

#### Differences by care delivery model and discipline

We observed differences in data visualization preferences across sites, likely reflective of their unique care delivery processes, mission, type of patient served, and mix of clinician disciplines. For example, clinicians at the community hospital and hospice (sessions 4 and 5) were, overall, more enthusiastic about the circular plot pain wheel, while this visualization, overall, was not well received in all 3 sessions with academic medical center clinicians. Interestingly, the hospice team was the only group of participants to raise questions about the ethical aspects of data sharing. For example, hospice clinicians raised legitimate and important concerns about what data should be shared with whom, and when. If, for example, a caregiver has rated their quality of life very low and their overall distress levels very high, should this be shared with the patient? Or vice versa? Should some data only be shared with clinicians—and if so, is that overly paternalistic given recent trends toward more open-access personal health data? Hospice staff verbalized the importance of not just “pushing out data” to patients/caregivers but being with patients/caregivers when they view the data so it can be explained and contextualized by the clinician. Likewise, while the hospice team, overall, liked the “good day, bad day” data (Table 5, column A, data visualizations #4 and #5), they had concerns about how sharing these data may influence care or the illness trajectory in unintended ways. Specifically, they expressed that some quality-of-life data may not be appropriate or helpful to share with the caregiver as hospice care is designed to shift attention away from a focus on these metrics and more to the comfort of the patient. As one hospice clinician participant explained, “I’m not sure we’d want to collect this information or have patients and caregivers focus on it, as we try to reduce the fixation on things like appetite as we know they will decline during the course of the illness.” Additionally, the hospice team discussed the importance of benchmarking responses against the patient's goals and wanted this information included within the data visualization to help contextualize the information. For example, if a patient experienced baseline high levels of severe chronic pain, then a 5/10 pain score may be considered “great” for them, and they may not take medication. In contrast, a patient with pain severity typically at a 2/10 would likely be struggling with a pain severity level of 5/10.

Overall, non-physician participants (nurses, social workers) expressed greater interest in visualizations that included environmental variables impacting pain and non-pharmacological interventions, along with more interest in caregiver data. Some clinicians viewed the visualizations not just through the lens of their own disciplinary perspective, but also considered how the visualizations would be received by patients/caregivers and how they could be helpful. For example, in session 4 a participant recognized that the circular plot (data visualization #6) could be very helpful for family members planning resources and knowing when the patient may need the most assistance.

#### Mixed interest in viewing caregiver data

There was mixed interest in viewing caregiver data. Overall, participants expressed a greater receptivity to seeing caregiver reported data about the patient (e.g., caregiver reporting about their perception of patient's pain) vs. caregiver reported data about themselves (e.g., caregiver reporting about their own quality of life, such as how much they, as caregivers, are sleeping). During the hospice feedback session, one participant upon viewing the caregiver's self-reported quality of life data stated, “I’m unclear what here would be actionable.” One participant from session 1, emphasized the importance of including caregiver-reported data about patients, stating, “it is going to be very useful in certain circumstances, particularly when the patient is confused or unreliable for other reasons.” During session 3, some participants expressed the caregiver self-reported quality-of-life visualizations were helpful, noting that caregivers may feel guilty or be reluctant to divulge how much they are actually struggling or how bad things have really been in front of the patient.

## Discussion

Our results contribute to the broader literature regarding sharing patient reported outcome (PRO) data with clinicians. Specifically, we provide interdisciplinary perspectives across diverse clinical sites to visualizations generated from complex, remote health sensing data related to advanced cancer pain. Importantly, our findings can help inform future electronic health record (EHR) integration efforts, particularly in optimizing data visualizations to support clinical decision making. We also offer an approach to effectively collect both quantitative and qualitative data from busy clinicians, and document how clinicians respond to seeing holistic data about the pain experience from both the perspective of the patient and their family caregiver. In general, survey results and group discussion feedback were congruent (e.g., a visualization that scored low on the survey was also not reviewed favorably during the discussion) and strongly favored simpler data visualizations that summarize clinical information perceived to be most relevant, such as number of severe pain events and response to medications. Clinicians wanted to understand the “take home message” of a data visualization quickly without having to spend a lot of time deciphering the visualization; our most “successful” visualizations were ones where participants felt like they could most quickly grasp the information. Additionally, we found that offering options is important, which we attempted to do by presenting visualizations that could be viewed as stand-alone static figures as well as those that allow users to probe for more information using interactive features (e.g., hovering over a data point to learn more about it). Just as the BESI-C system is designed to deliver tailored pain management interventions, we found that data output from BESI-C must be tailored, too; for example, some individuals desire visualizations with high levels of granularity, others may not. An important lesson learned is the importance of tailoring visualizations based on the goals or roles of the intended clinical audience and presenting them accordingly during feedback sessions. Other recommendations based on our experiences generating and sharing data visualizations from BESI-C data with clinicians are summarized in Table 6.

Our findings reinforce much of what has been previously reported in the literature regarding clinician preference for simple, familiar visualizations (e.g., bar and line graphs), as summarized in Hancock et al's excellent scoping review (79). Similar to previous studies, we also found that less familiar visualizations were generally not as well received (79)—although after a brief explanation, some participants found these extremely helpful. Data presented in familiar ways was more likely to be better received and understood by clinicians. For example, overall, participants preferred more familiar bar charts and line graphs to the less-familiar circular plot (“pain wheel”, session 1, visualization #1) or the bubble plot (used to visualize environmental factors related to pain management, session 1, visualization #4) where we learned not everyone is comfortable interpreting correlational data. Interestingly, however, we found that an expressed preference for bar and line graphs didn't necessarily correlate with their increased comprehension, as measured by our objective comprehensive question for each visualization. This may be due to other factors that made interpreting the bar/line graphs we presented confusing (such as poorly worded axes) or a default/unconscious preference for more familiar types of data visualizations, even if they are actually unclear. In other words, what clinicians say they prefer may not always be what is best understood, or vice versa, and this is important to keep in mind when creating visualizations and seeking feedback. We also found that using a traffic-light color palette was helpful to orient clinicians quickly to thresholds related to symptom severity (80, 81). However, in contrast to previous studies (82, 83), our participant sample was less interested in seeing written summaries or explanations to help contextualize the visualizations; in fact, we generally found confusion regarding legends accompanying visualizations and a strong desire to be able to grasp the take-home message without needing to digest or read additional information.

Participants also drew connections and comparisons between visualizations they had seen before or in similar ways. For example, the “pain wheel” was especially well received by a participant who used an Apple Watch and had seen data represented in a similar way previously. Participants had varying degrees of comfort viewing and interpreting data visualizations and different levels of data literacy. Some participants were interested in “digging deeper” and wanted to understand the analysis behind the visualizations (e.g., how was the average calculated?). Others asked very astute questions that prompted us to rethink how data were presented, such as in session 5, visualization #6, where one participant pointed out that having all the times where pain events aren't recorded as set to zero (on the 0/10 pain severity Numeric Rating Scale) creates the assumption that the patient isn't having any pain at that time; their suggestion to grey-out time blocks where no pain event was being recorded was a helpful suggestion to distinguish between *not* reporting pain vs. reporting *no* pain. At times, participants asked questions that we had the data to answer but that we hadn't included for brevity or simplicity and these questions were helpful in validating our instincts of what features to include in future visualizations. Some participant suggestions were excellent, but not possible given time and scope of the current project (e.g., full integration with the electronic health record system); keeping a list of these suggestions for future work is recommended.

In general, clinicians were more interested in outcomes related to the physical domain of pain (e.g., severity levels; medication use; impact of pharmacological interventions) than psychosocial factors related to quality of life, environmental variables impacting pain, or non-pharmacological interventions. The limited interest/preference in viewing psychosocial data underscores the reality that despite efforts to challenge the biomedical model of pain care in the U.S., practice commonly lags behind theoretical advancements (84, 85). Few care delivery models, even in highly resourced contexts, are adequately equipped to holistically support the emotional, psychological, and social challenges we have long known are associated with difficult physical pain (86–88). Given these constraints, it is understandable, albeit unfortunate, that clinicians may focus on physical aspects of pain management that can be perceived as more straightforward to measure and treat. For example, we were struck by the importance of pain severity as the preferred metric for assessing pain that was evident in both the quantitative and qualitative findings. Across all groups, clinicians were most interested in pain events that were “severe” (although the perceived cut-off value for “severe” varied, for some it was ≥5/10, for others ≥7/10) and some participants suggested omitting any information about pain events that did not rise to this level. While pain severity is clearly a primary assessment parameter, research has shown that frequency of pain events (even of lower-intensity pain events) can be equally, if not more, important in assessing and understanding the pain experience (89). A limited focus on high severity pain events could be particularly problematic for stoic patients, or for those who may struggle to use the traditional 0–10 Numeric Rating Scale. One solution if using interactive visualizations could be to include all reported pain events, regardless of severity score, but offer a filter to allow clinicians to select the pain severity threshold they feel is most relevant for a particular patient.

Not surprisingly, we found that preferences for, and interpretations of, data visualizations were influenced by the model of care delivery (e.g., hospice vs. academic medical center vs. community hospital) and the expected involvement of family caregivers (e.g., hospice relies heavily on family caregivers to participate in and oversee a patient's care, whereas in the academic medical center and community hospital care is primarily overseen by clinical staff). There were qualitative differences in how data visualizations were viewed between sites and disciplines, and we recommend tailoring visualizations to specific care sites and disciplines, recognizing that certain data and visualizations may be highly appropriate and helpful in one context, but not so much in another. A good example of this relates to the astute feedback we received from hospice clinicians about concerns related to data sharing that may not be aligned with the organizational ethos or that may create unintended distress. For example, data related to declining appetite, an expected outcome at the end-of-life that would likely not warrant intervention in hospice, could be an important metric for intervention for a patient on a clinical trial at an academic medical center. Overall, we found that community hospital and hospice clinicians had a higher tolerance for detailed visualizations and responded more favorably to complex representations of pain data. The hospice team was especially interested in correlational data (e.g., “how is activity affecting pain?”) and seeing more granular data details related to pain. Whether these differences are related to in-person vs. virtual feedback sessions (both the hospice and community hospital session were held in person, whereas the academic medical center sessions were all over Zoom) or is more a function of different care philosophies or care delivery models, is difficult to fully assess.

Some visualizations were just simply not well-received. For example, the Sankey diagram, shared first in session 1 and then iterated and shared again in session 2, received few positive comments and did not score well in the survey. After session 2, we abandoned it in favor of developing new visualizations. This decision was difficult for some members of our research team who felt the Sankey diagram was an important way to view reasons patients may not have taken pain medication. From this, we learned some data visualizations may have significant merit in a research context or as an educational tool for clinicians but may not translate well to the bedside. While it may seem obvious in retrospect, an important lesson for our team in presenting complex data is that no one data visualization can be all things to all people. For example, the circular “pain wheel” plot visualization continued to receive very mixed, and mostly negative, reviews with our academic medical center participants regardless of how we iterated and revised it—but it was extremely well received by the community hospital and hospice participants.

We found that first impressions were paramount. For some participants it was clear that once they had made up their mind that a particular visualization was not helpful, no amount of iteration or improvement was likely to alter their perception. Bearing in mind that first impressions matter when sharing data visualizations, there are some easy steps that can be taken to achieve a more polished look from the start; for example, such as using a consistent color palette and font across visualizations; ensuring axes are clearly labeled; and providing enough context about the key message and source of data with clear and succinct titles (e.g., “Patient-Reported Pain Events—Past 14 days'” or with a question, “How many severe pain events did patient experience over the past 14-days?”). These are some strategies we found useful to prevent formatting details from becoming distractors and precluding more constructive feedback.

We were surprised by the tepid reception to caregiver reported data, as we anticipated there would be greater clinician interest in this unique aspect of BESI-C data. Participants acknowledged that caregiver data are important, but expressed an uncertainty about what to do with it or how to use it. While most participants agreed there was value in seeing data about the patient as reported by the caregiver, there was markedly less interest in seeing caregiver self-reported data about their own (caregiver) quality of life. This speaks to broader concerns, well documented in the literature, and beyond the scope of this paper, about the many unmet needs of family caregivers and care partners (90–93). Lack of interest in caregiver self-reported quality of life data may also reflect the reality that clinicians are not used to receiving such information and feel unprepared as to how they would begin to address these needs. This reaction may be akin to the reluctance of clinicians to ask about sexual health or intimacy concerns when they feel they cannot offer feasible solutions (94, 95) (i.e., “isn’t it worse to open up a can of worms if I can’t do anything to help?”). This finding reinforces significant gaps in our healthcare system and the critical need to improve support to caregivers through innovative models of care delivery, such as offering point-of-care support to caregivers in parallel with patient appointments. For example, a palliative care clinician could serve as the provider of record for both the patient and caregiver and address focused quality of life caregiver needs during patient visits, negating the need for an already overburdened caregiver to schedule and attend additional medical appointments. Clearly, such a model would require workflow and reimbursement restructuring, but could be a feasible way to empower clinical teams to do more to support caregivers, which, in turn, benefits patients.

### Lessons learned about conducting the feedback sessions

We made some practical discoveries regarding the data visualization feedback sessions that may be helpful to others engaged in similar work. Gathering data from busy clinicians is not easy due to scheduling and competing priorities, and it was important to make it as easy and convenient as possible. Tagging onto existing staff meetings when feasible was immensely helpful with scheduling, and a generous offer by clinician partners (underscoring the importance of having clinical champions at recruitment sites). With limited time for each session, we had to make tough decisions about which visualizations (e.g., new ones each time or iterations of previous visualizations? We ended up using a combination approach) and how many to present. With the in-person sessions we were able to get through more (6 visualizations); with the virtual sessions, sometimes we only had time for 5.

Overall, we found in-person feedback sessions richer, more interactive, and more helpful in iterating data visualizations and we highly recommend this format when possible. In-person sessions had a more organic and natural flow of conversation, with the added benefit of being able to better read non-verbal cues and confirm participants were all viewing the same visualization in the same way at the same time. However, one advantage of virtual sessions, in addition to scheduling convenience, was the ability to demonstrate interactive features of data visualizations more easily over a shared screen. We also strongly suggest starting a visualization feedback session with “easier” or “more familiar” visualizations; in hindsight, beginning sessions 1 and 2 with the less-familiar circular plot (pain wheel) was probably not the best strategy and may have impacted its reception; in sessions 3–5 we changed tacks and began by presenting more infographic-style visualizations as a “warm-up”; these were generally very well received, and participants commented on their simplicity and ease of understanding.

### Future directions

Future work should focus on feasible approaches to integrate RHMS data visualizations into electronic medical records and creating a library/dashboard of data visualizations that can prioritize feature customization, such as allowing users to select “low, medium, or high” degrees of data granularity or presentations of data in different formats (e.g., bar graph vs. circular plot). An important related aspect of this work involves sharing data visualizations with patients and caregivers; those findings will be the basis of a subsequent paper. Additionally, exploring ethical issues related to sharing of data visualizations, and solutions to ensure privacy, is also critically important (e.g., which data visualizations should be shared with whom, when, and in what ways) and another priority area for future work.

### Limitations

We iterated data visualizations between feedback sessions, and presented them in different sequences, which limits direct comparisons between sessions and likely influenced participant perceptions. We also added a few survey items in later feedback sessions to capture valuable information related to data sharing. Additionally, because surveys were anonymous, and not linked to individual participants, we were unable to aggregate demographic data for the total sample (e.g., the same participant could have attended sessions 1 and 2; thus, aggregating their demographic data would inflate our participant numbers and misrepresent our demographic results). Our sample size for each feedback session—while consistent with focus group size recommendations (96)—precludes statistical comparisons within, or between, groups. We also did not have the same number of feedback sessions at each study site due to time and logistic constraints. We would have liked to share more iterations of visualizations during each feedback session, but time constraints limited how many were feasible to discuss during the 1-hour time block. Additionally, we were unable to find a brief, validated assessment tool to quantitatively assess clinician preferences related to data visualizations and ultimately created our own that focused on clarity, usefulness, and comprehension. Lastly, our clinician sample was primarily White and non-Hispanic, and we did not capture perspectives on data visualizations that may differ based upon diverse sociodemographic factors, an important limitation.

## Conclusion

Finding meaningful ways to share complex, holistic, remote health sensing data with clinicians is critical to improve cancer pain management support for both patients and family caregivers. We discuss an approach to creating and sharing data visualizations from a novel remote health monitoring system (BESI-C) with palliative care clinicians related to pain experienced at home by patients with advanced cancer. Orienting clinicians to unfamiliar data sources (such as environmental data and caregiver self-reported quality of life data) and integrating these data into clinical workflows and electronic health records is critical to ensure remote sensing data can optimally improve health outcomes and strengthen communication between clinicians, patients, and caregivers. Future work aims to create a library of data visualizations with a customizable range of viewing options to meet the needs of diverse, interdisciplinary clinical audiences; gather feedback from patients and caregivers; and explore ethical issues related to sharing data visualizations.

## Data Availability

The raw data supporting the conclusions of this article will be made available by the authors, without undue reservation, in compliance with institutional guidelines that ensure protection of participant privacy.
