# Supplementary material for: “Less words, more pictures”: creating and sharing data visualizations from a remote health monitoring system with clinicians to improve cancer pain management
Source: Front Digit Health. 2025 Apr 23;7:1520990. doi: 10.3389/fdgth.2025.1520990 (PMC12055813; doi:10.3389/fdgth.2025.1520990)

Data Supplement 1: Data visualizations shared during clinician feedback sessions, 1-5.

Session 1

Data Visualization #1

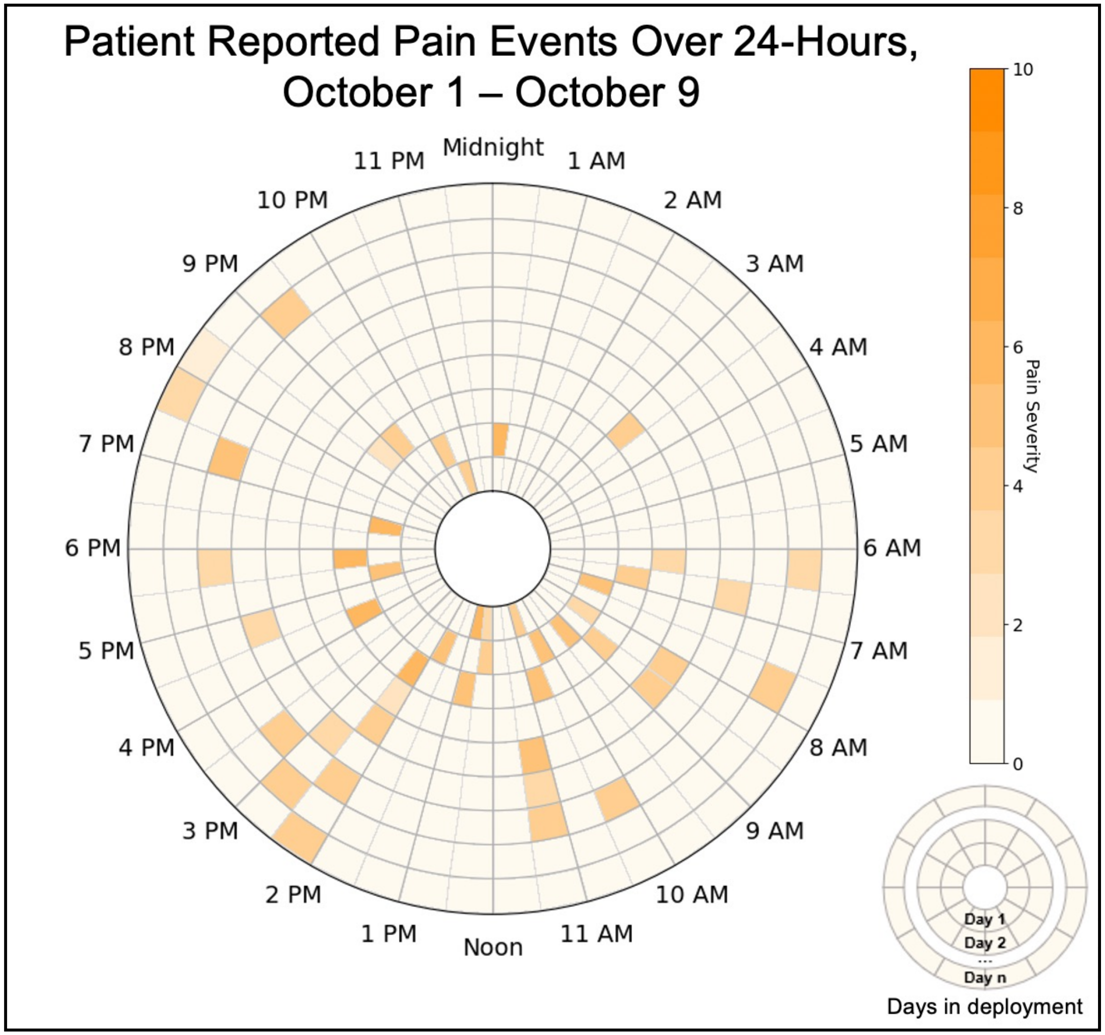

Session 1
Data Visualization #2

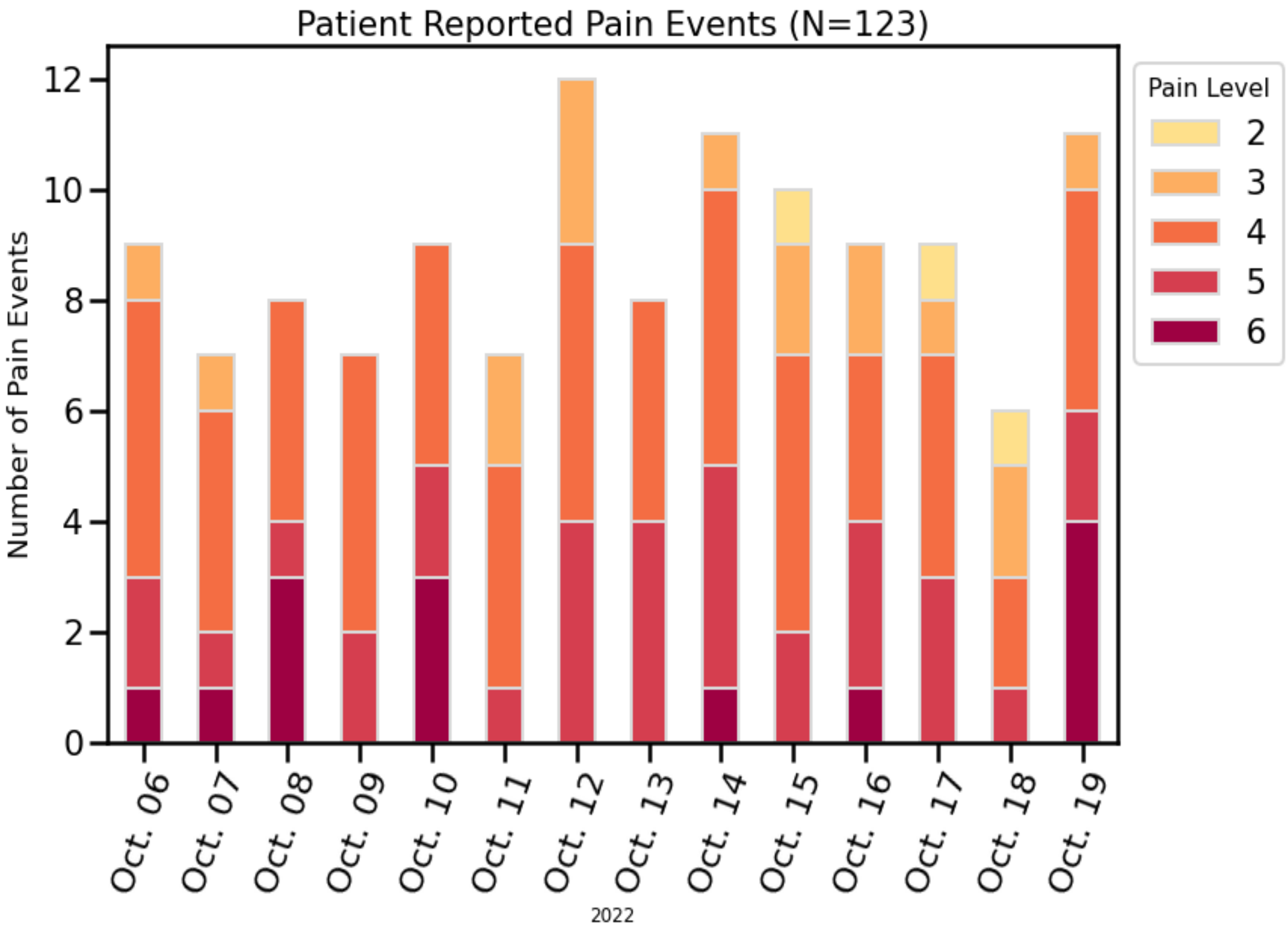

Session 1

Data Visualization #3

Patient Responses to Pain Events

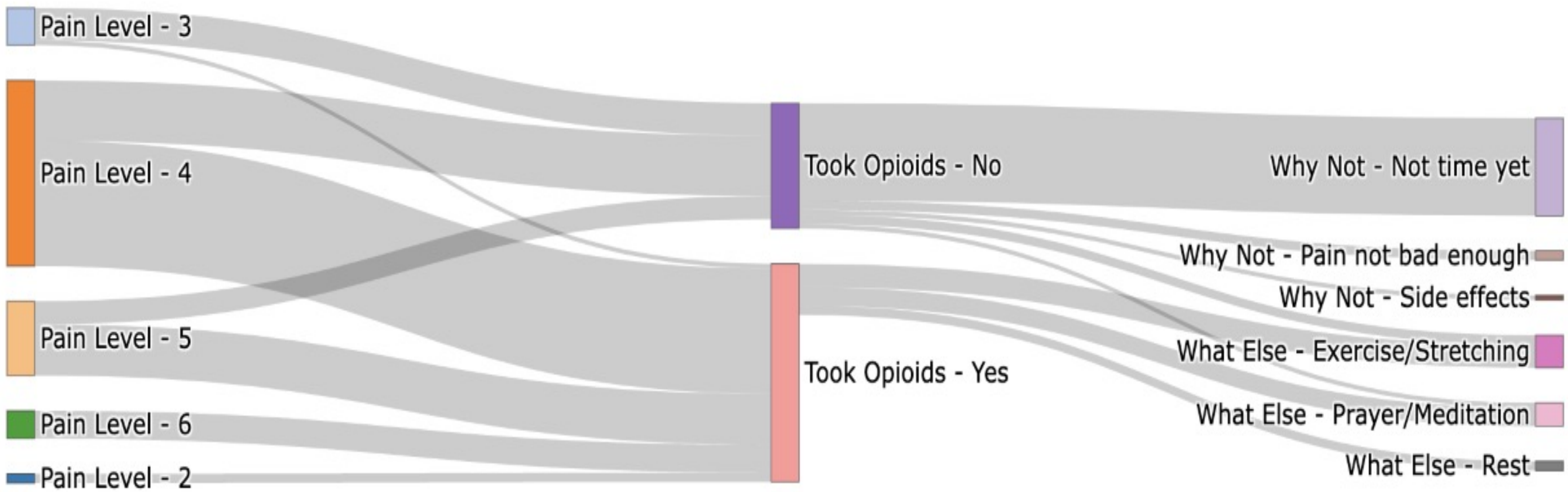

Pain Level on Scale of 0-10

Session 1
Data Visualization #4

Relationship between environmental factors and pain severity,

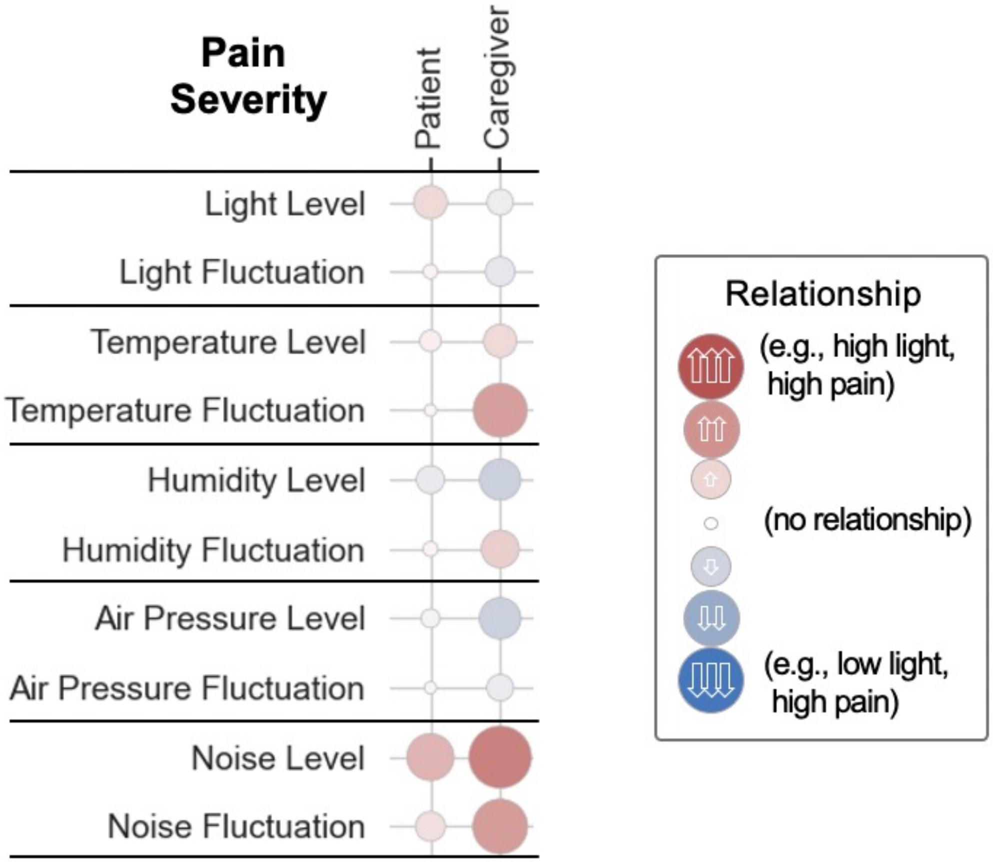

Session 1
Data Visualization #5

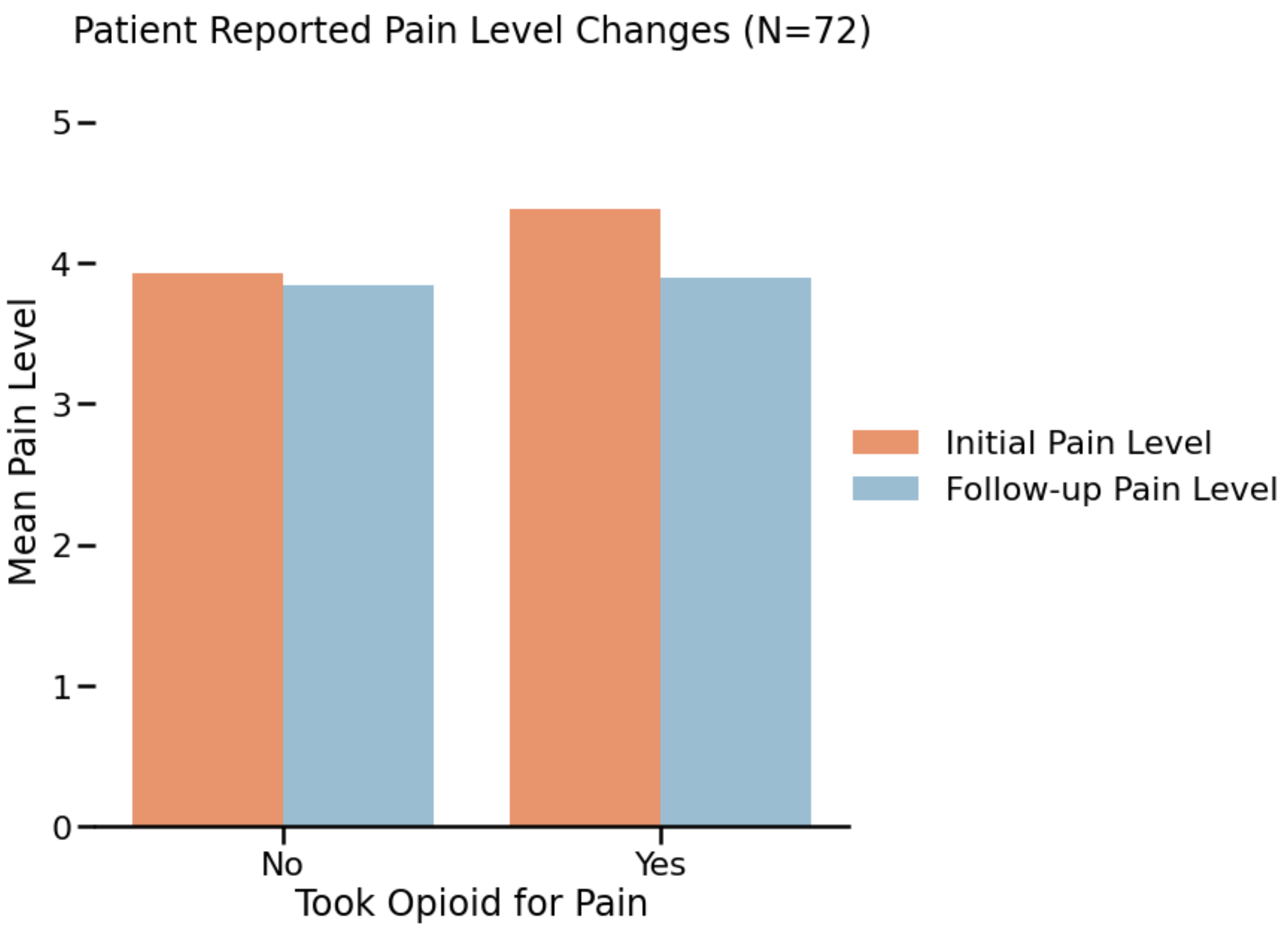

Session 2
Data Visualization #1

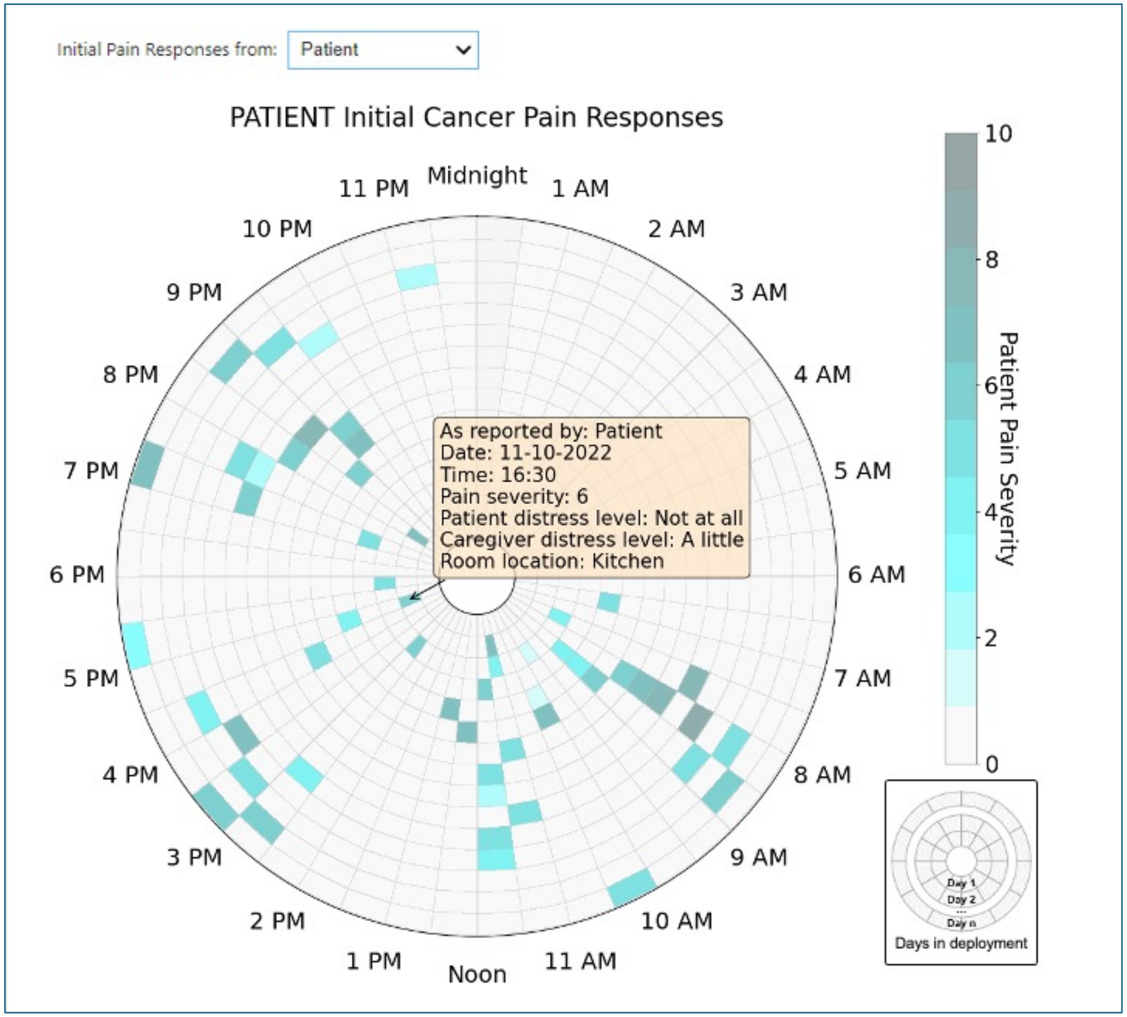

Session 2

Data Visualization #2

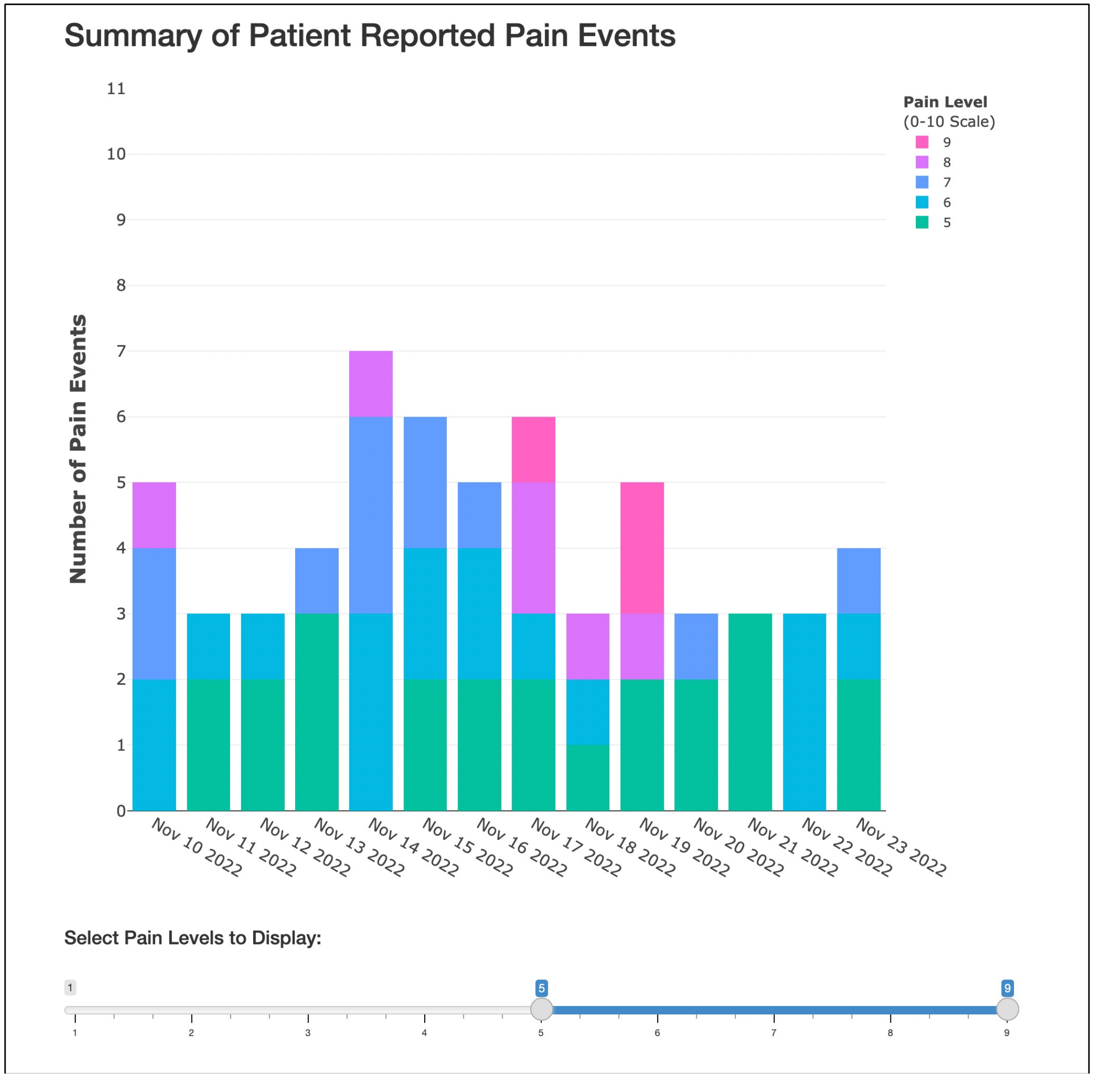

Session 2
Data Visualization #3

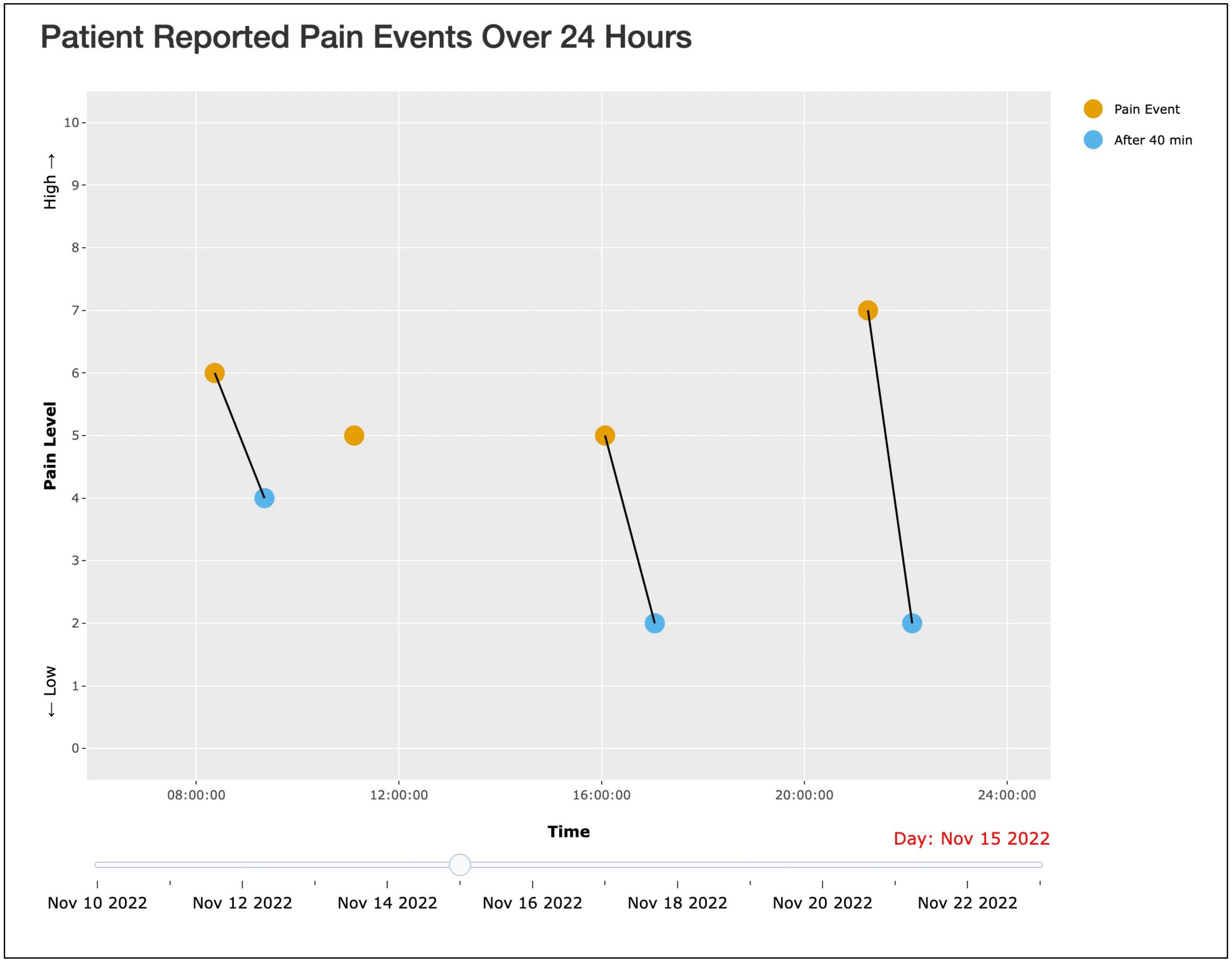

Session 2
Data Visualization #4

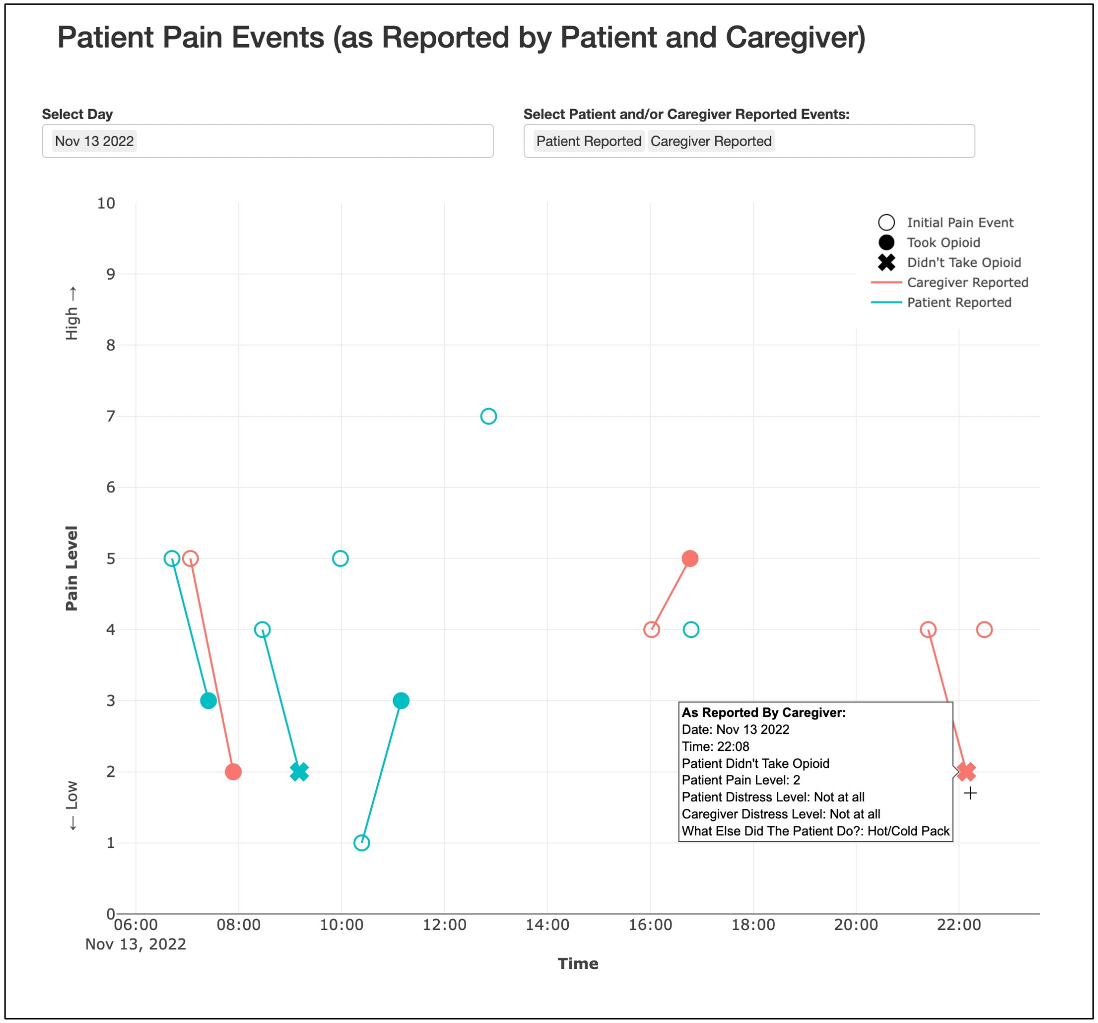

Session 2
Data Visualization #5

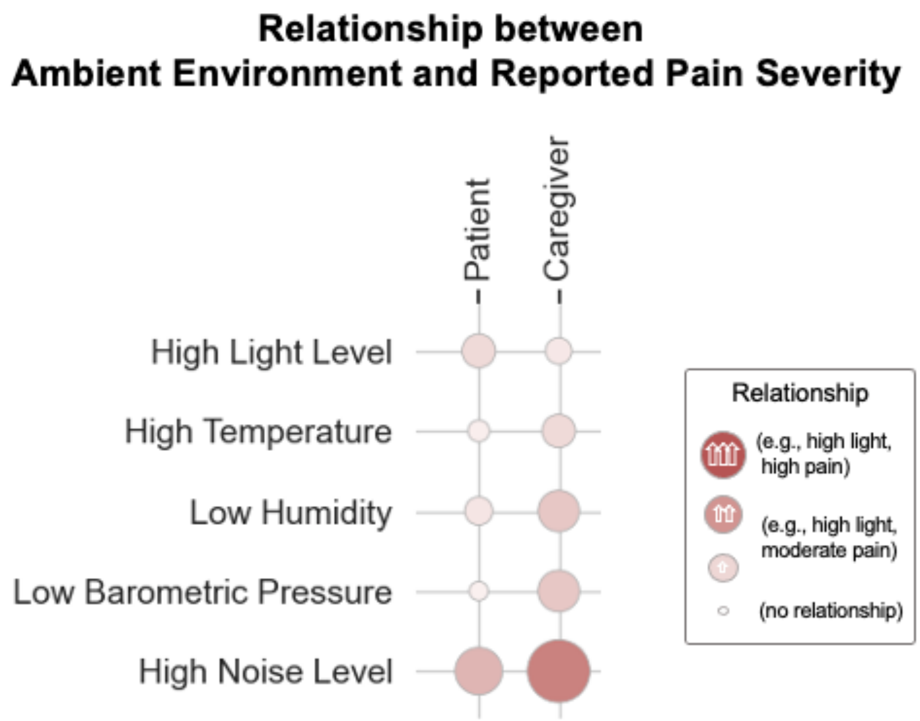

Session 2

Data Visualization #6

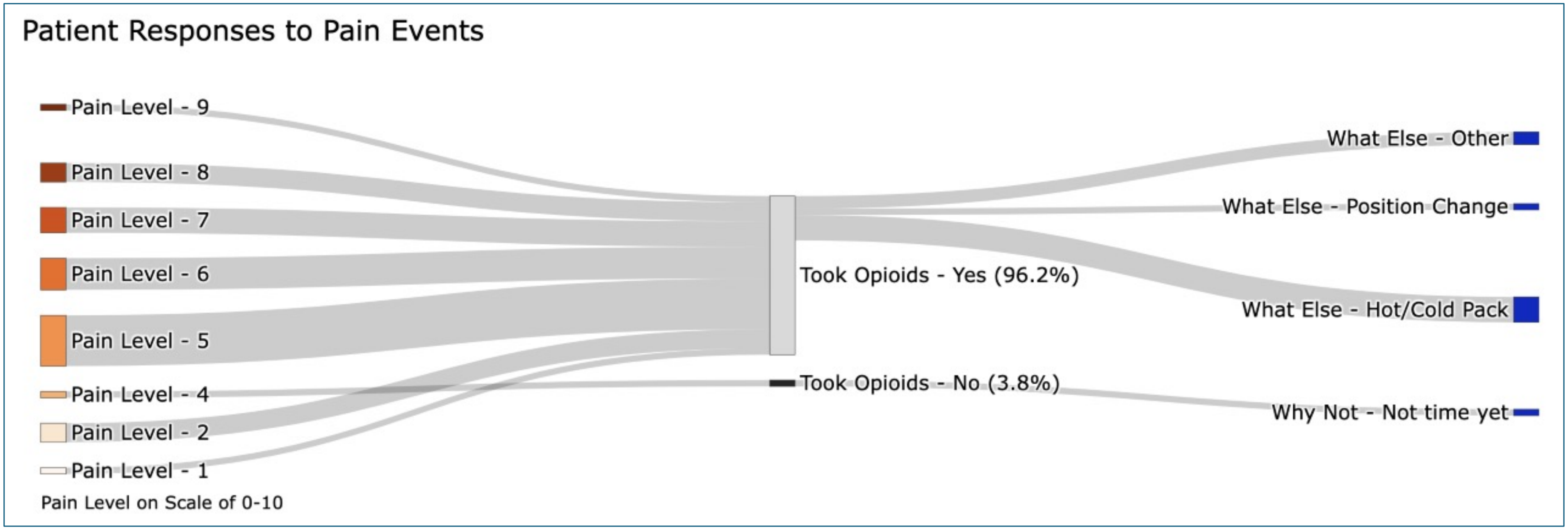

Session 3

Data Visualization #1

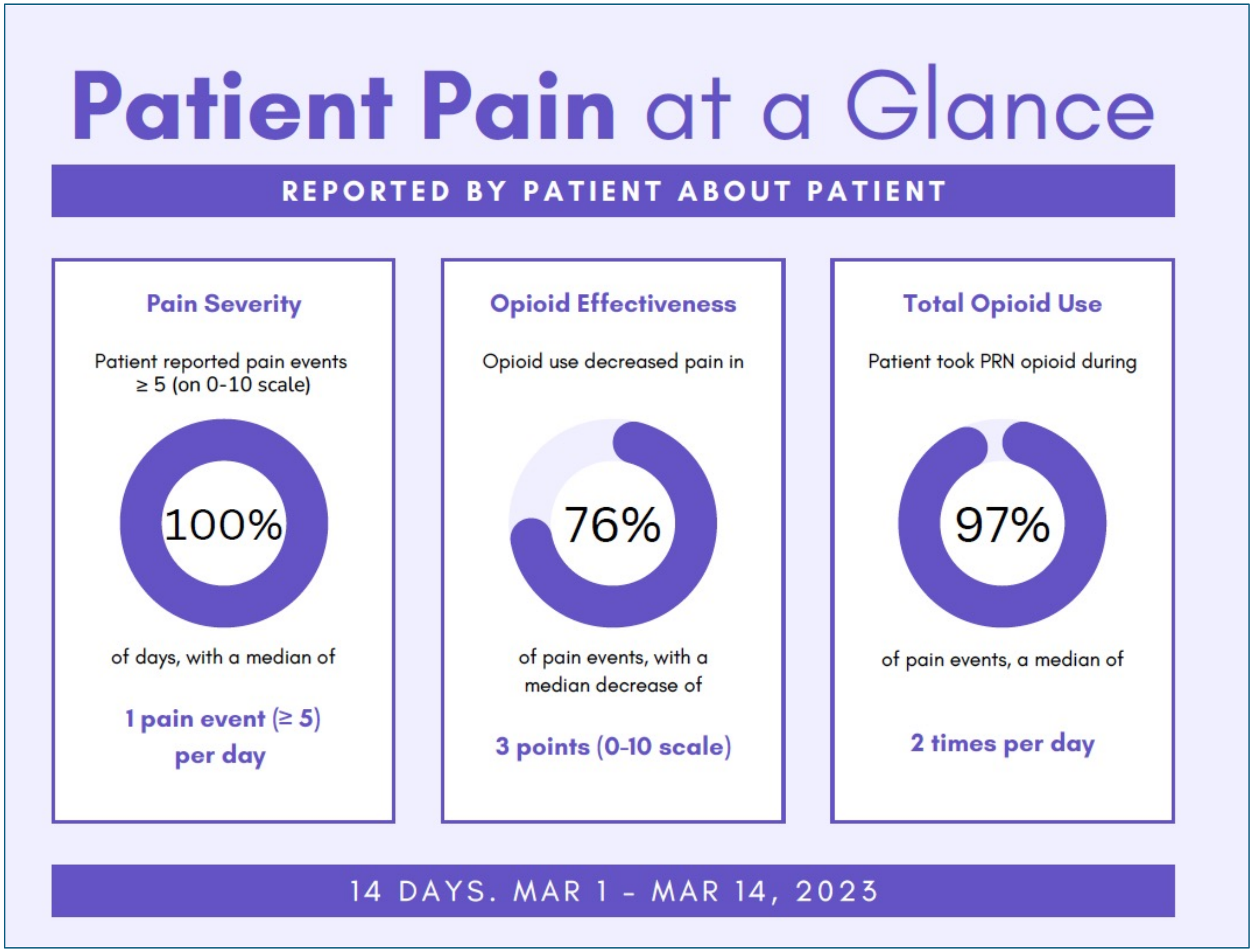

Session 3
Data Visualization #2

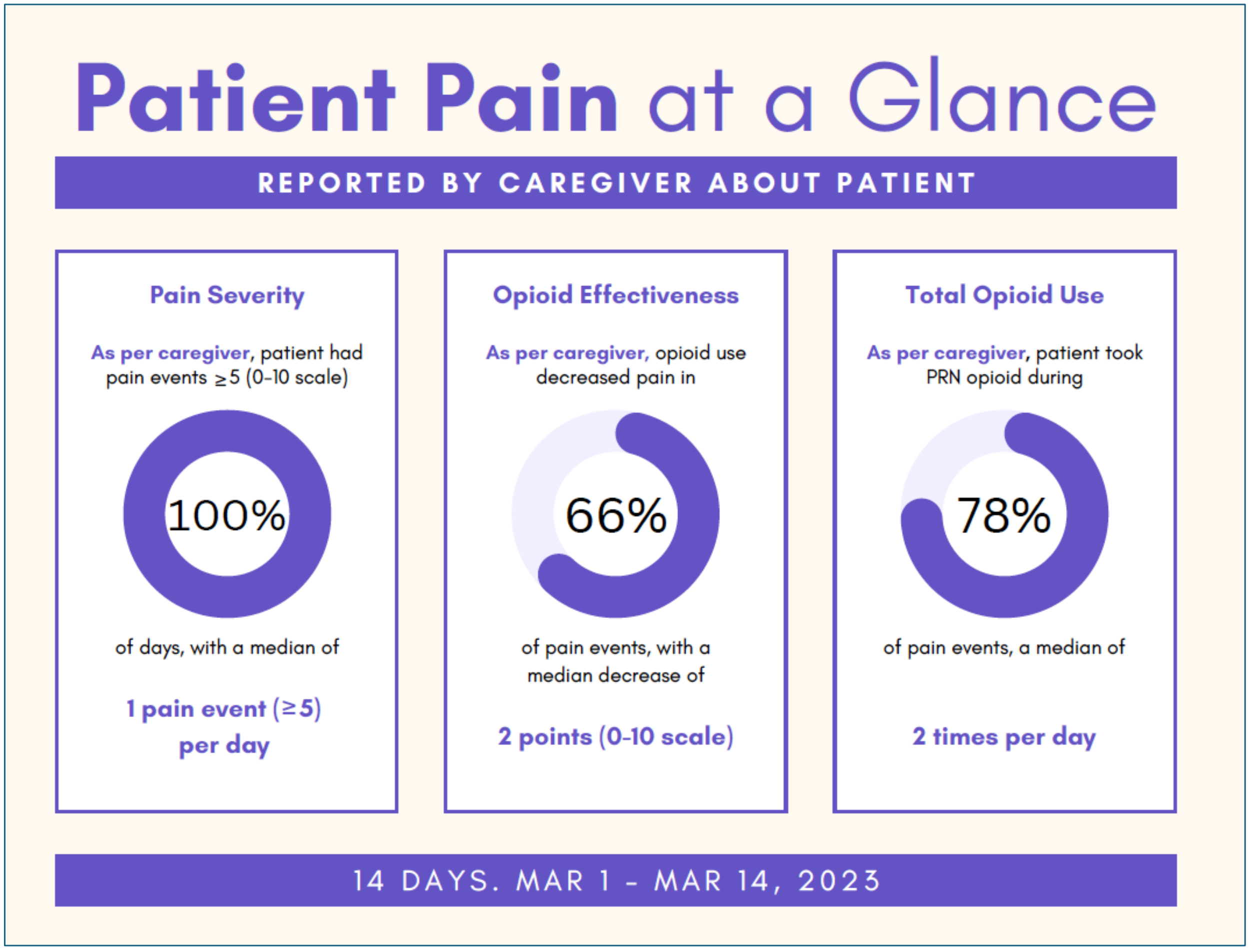

Session 3

Data Visualization #3

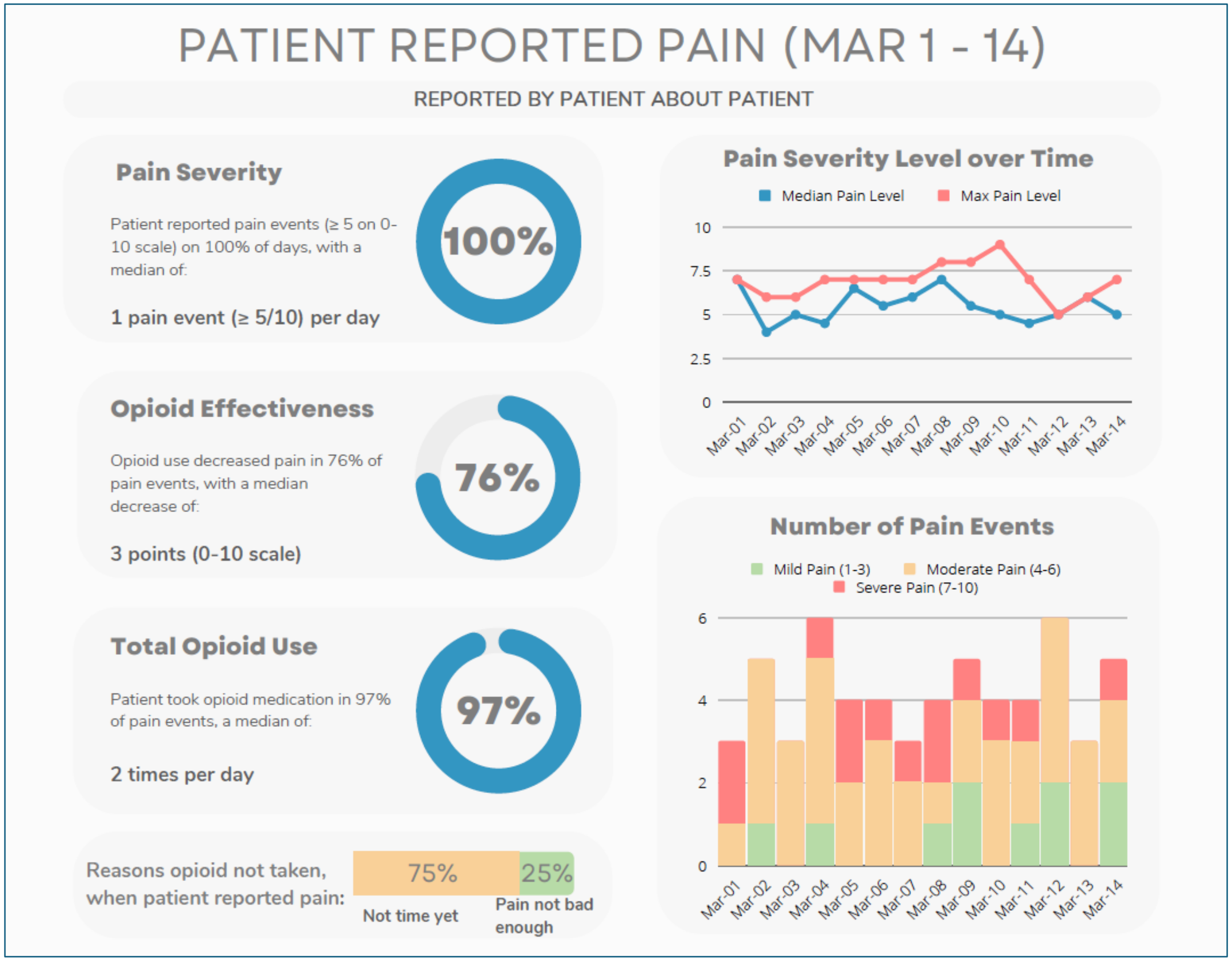

Session 3
Data Visualization #4

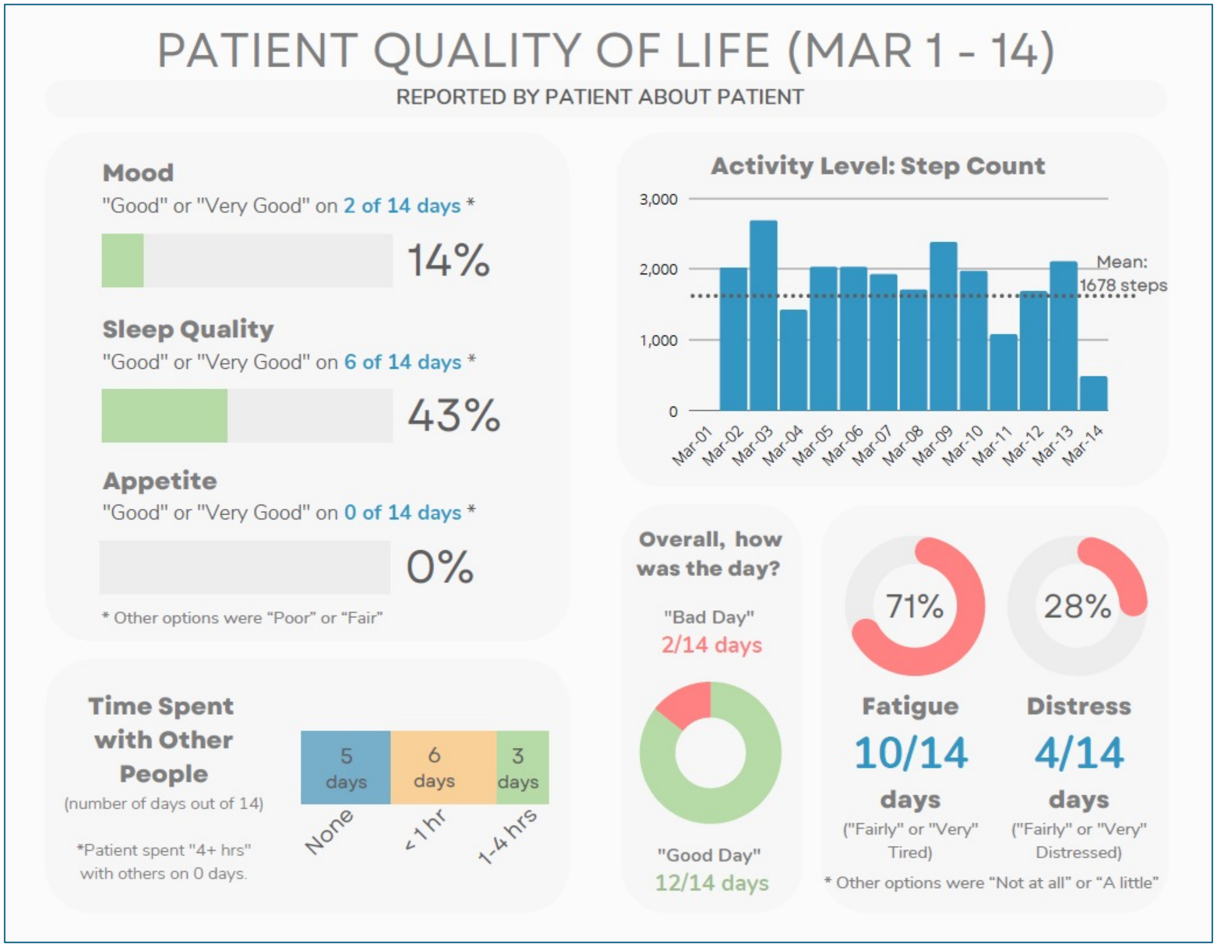

Session 3

Data Visualization #5

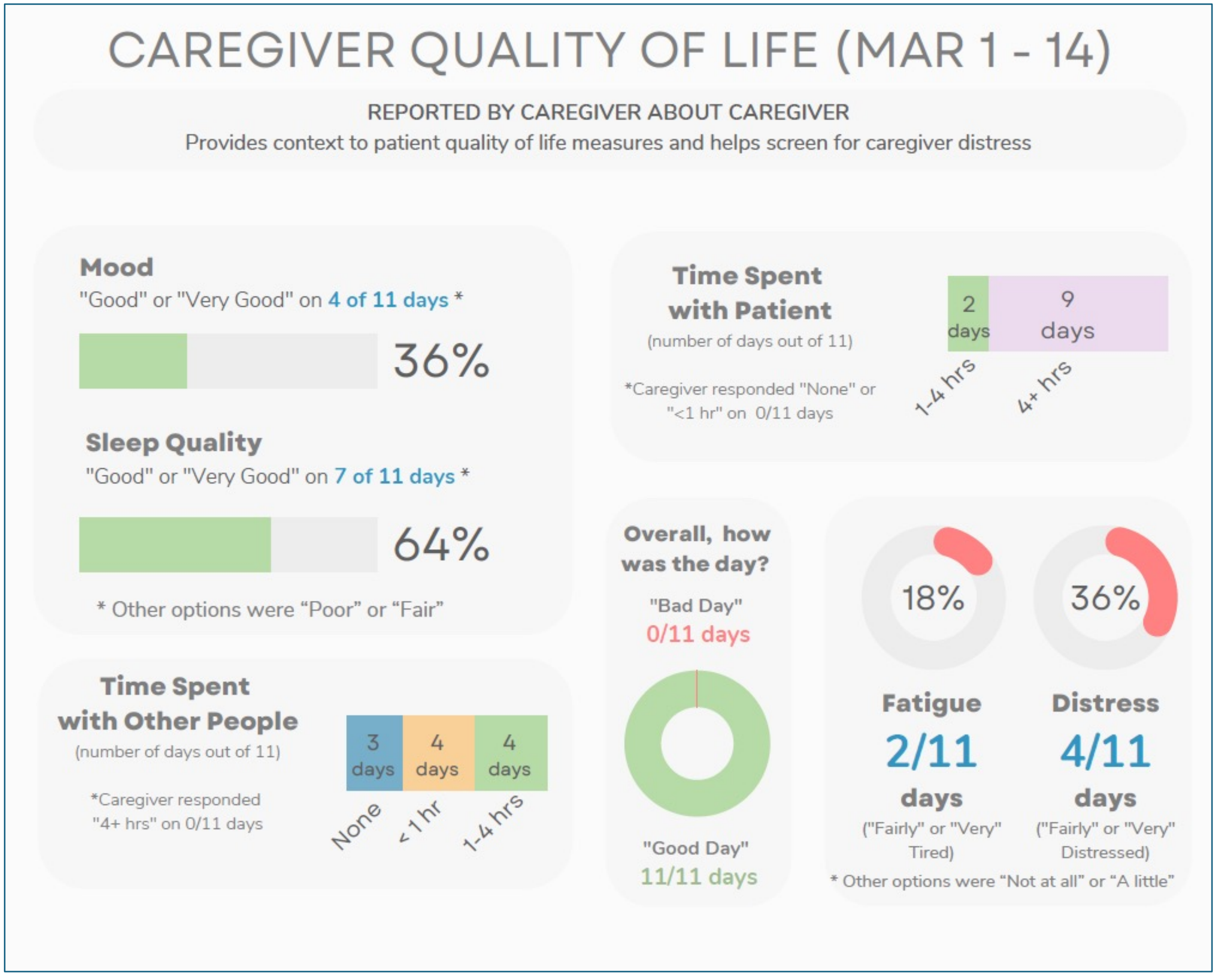

Session 4 & 5

Data Visualization #1

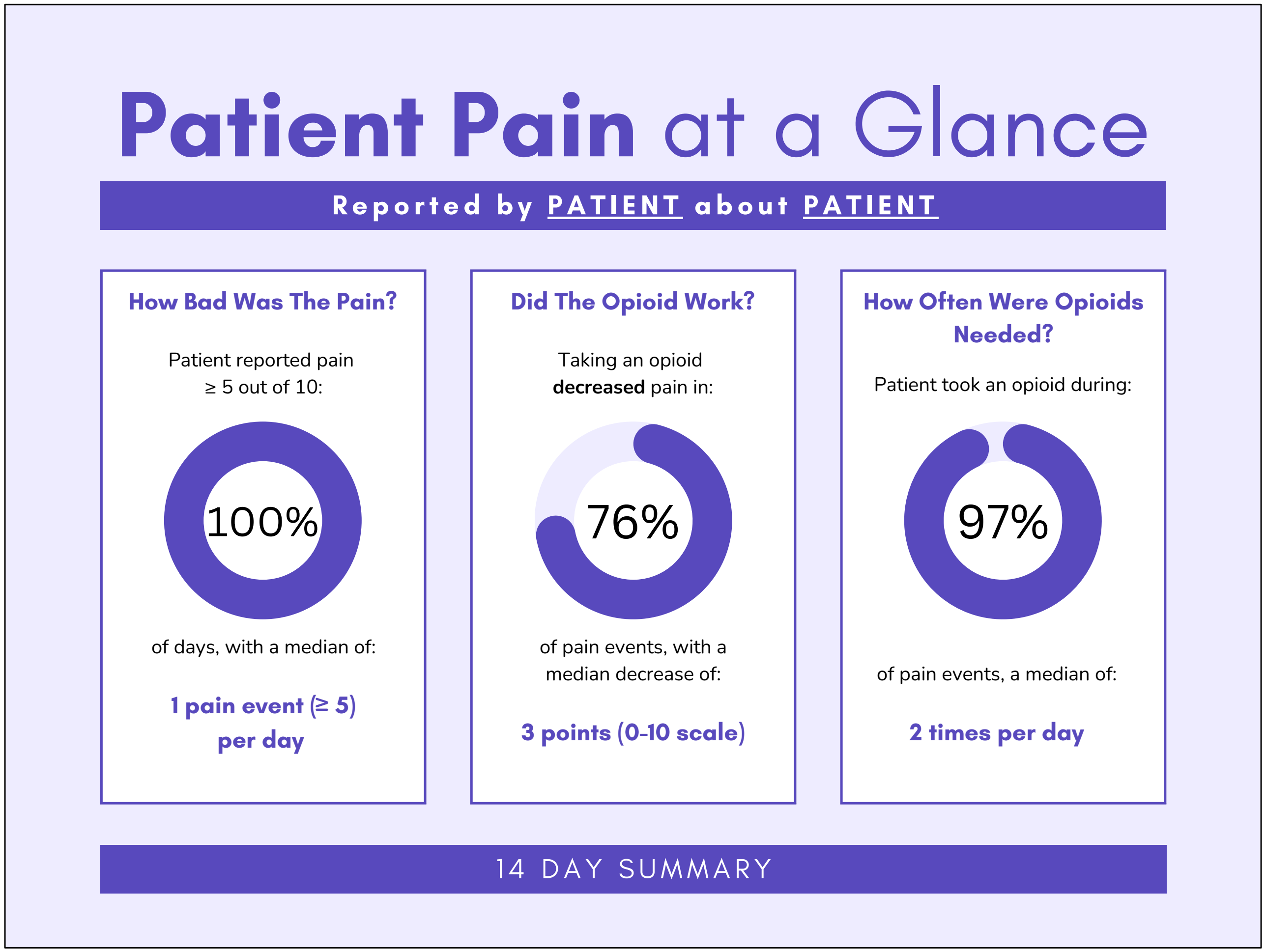

Session 4 & 5
Data Visualization #2

Patient Pain at a Glance

Reported by CAREGIVER about PATIENT

How Bad Was The Pain?

As per caregiver, patient pain was ≥ 5 out of 10:

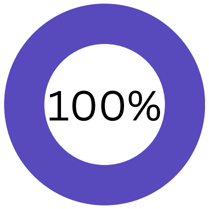

of days, with a median of:

1 pain event (≥ 5) per day

Did The Opioid Work?

As per caregiver, taking an opioid decreased pain in:

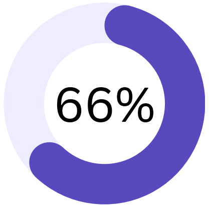

of patient pain events, with a median decrease of:

2 points (0-10 scale)

How Often Were Opioids Needed?

As per caregiver, patient took an opioid during:

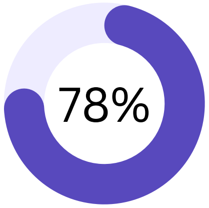

of pain events, a median of:

2 times per day

14 DAY SUMMARY

Session 4 & 5

Data Visualization #3

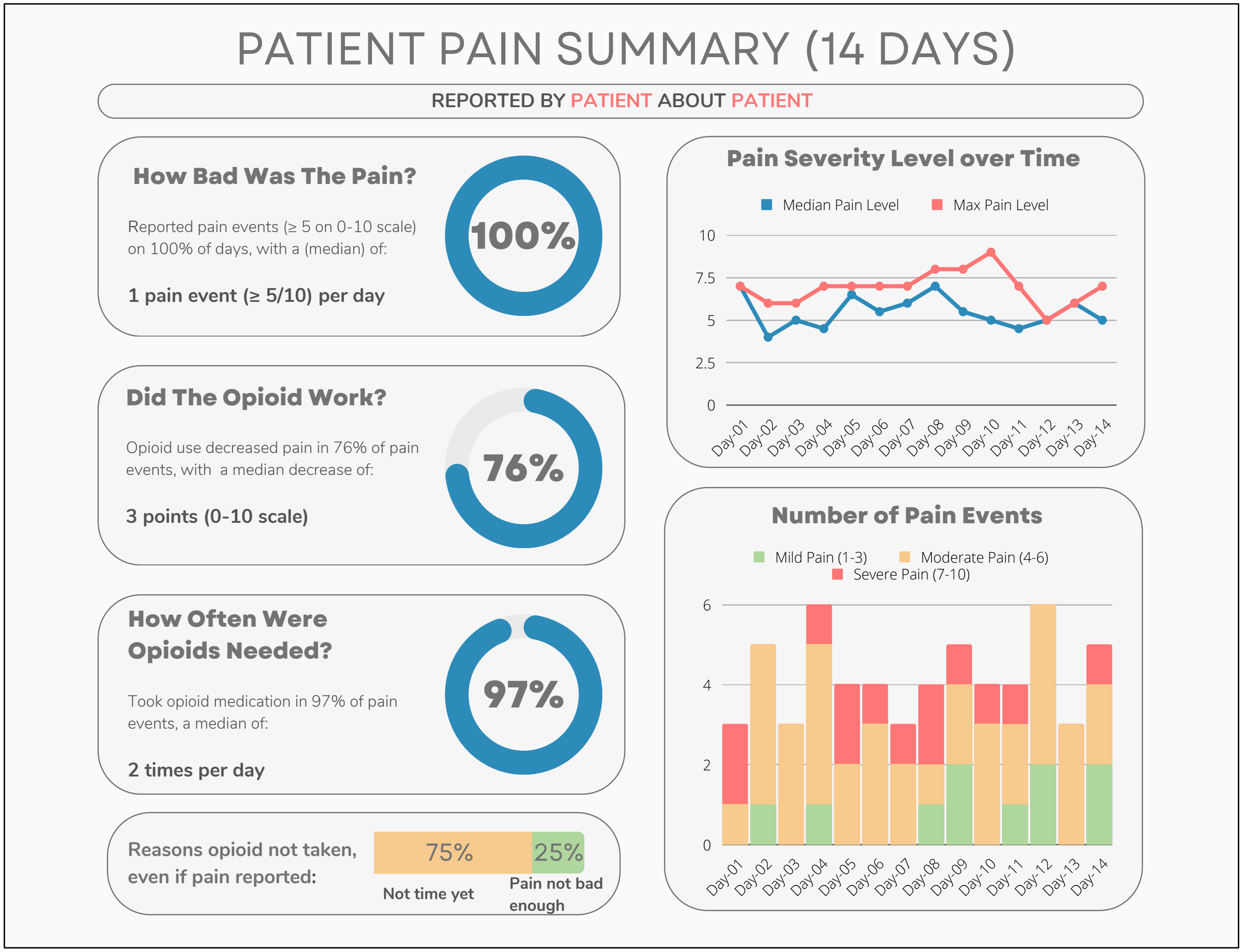

Session 4 & 5
Data Visualization #4

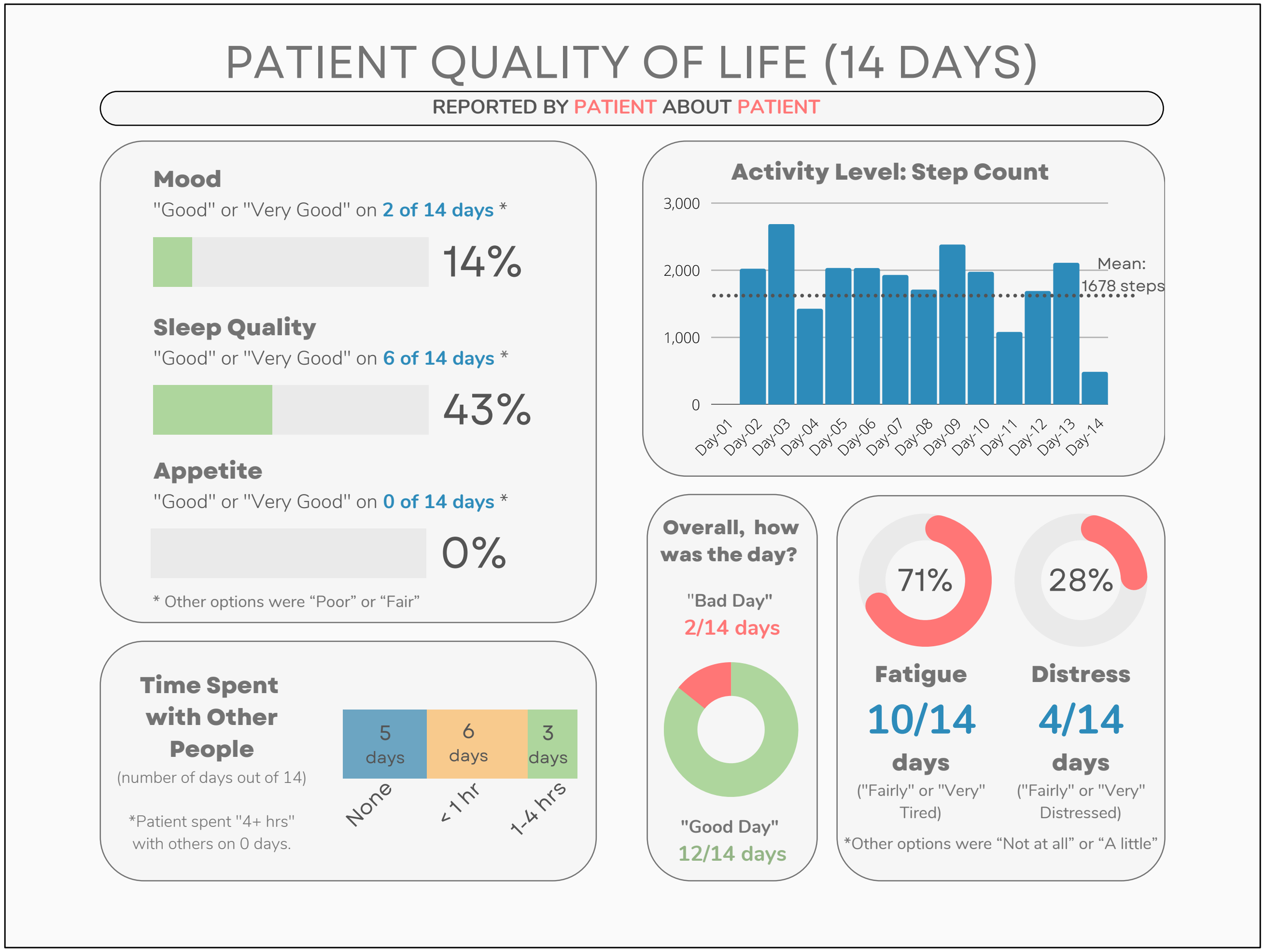

Session 4 & 5
Data Visualization #5

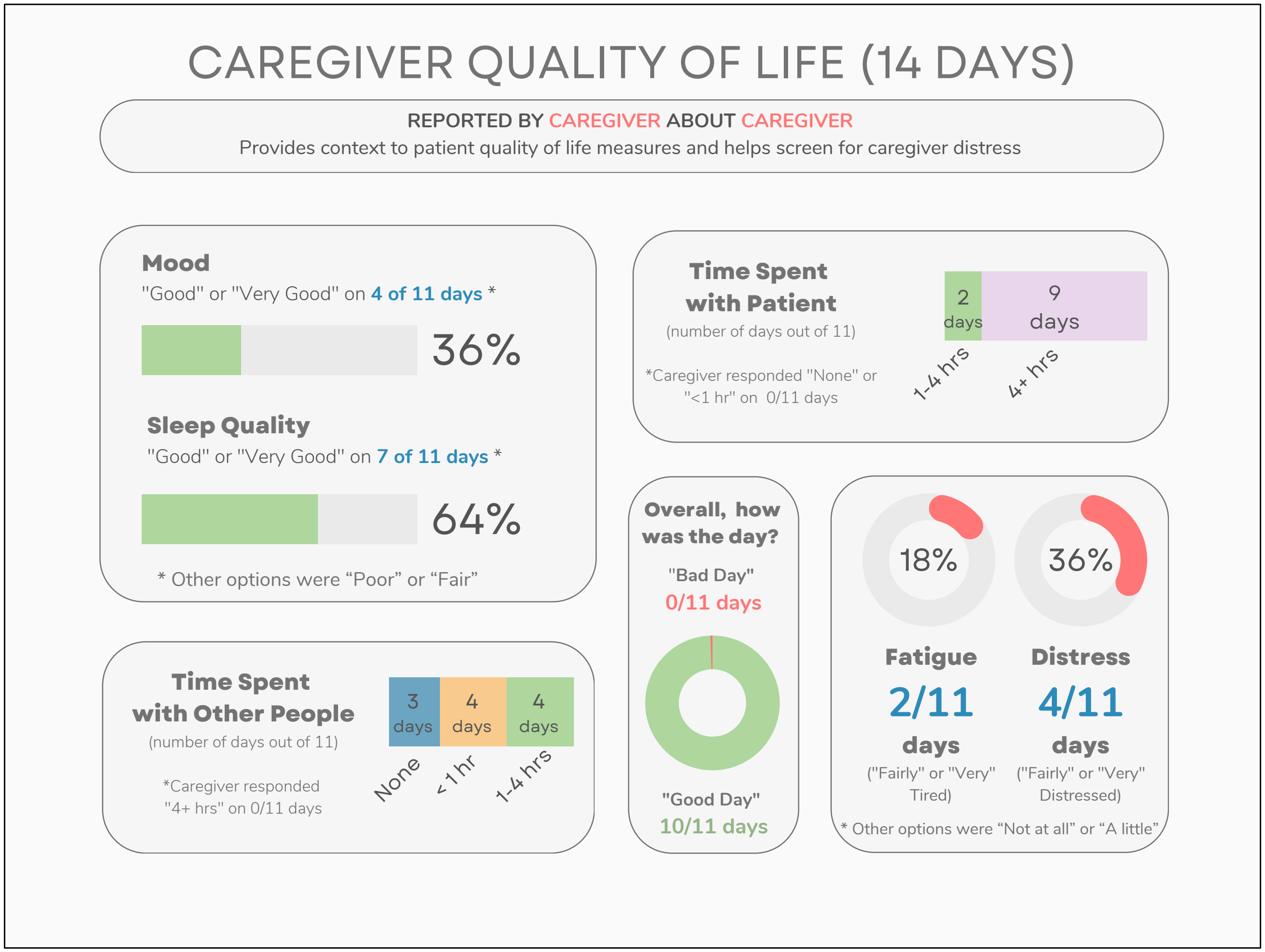

Session 4 & 5
Data Visualization #6

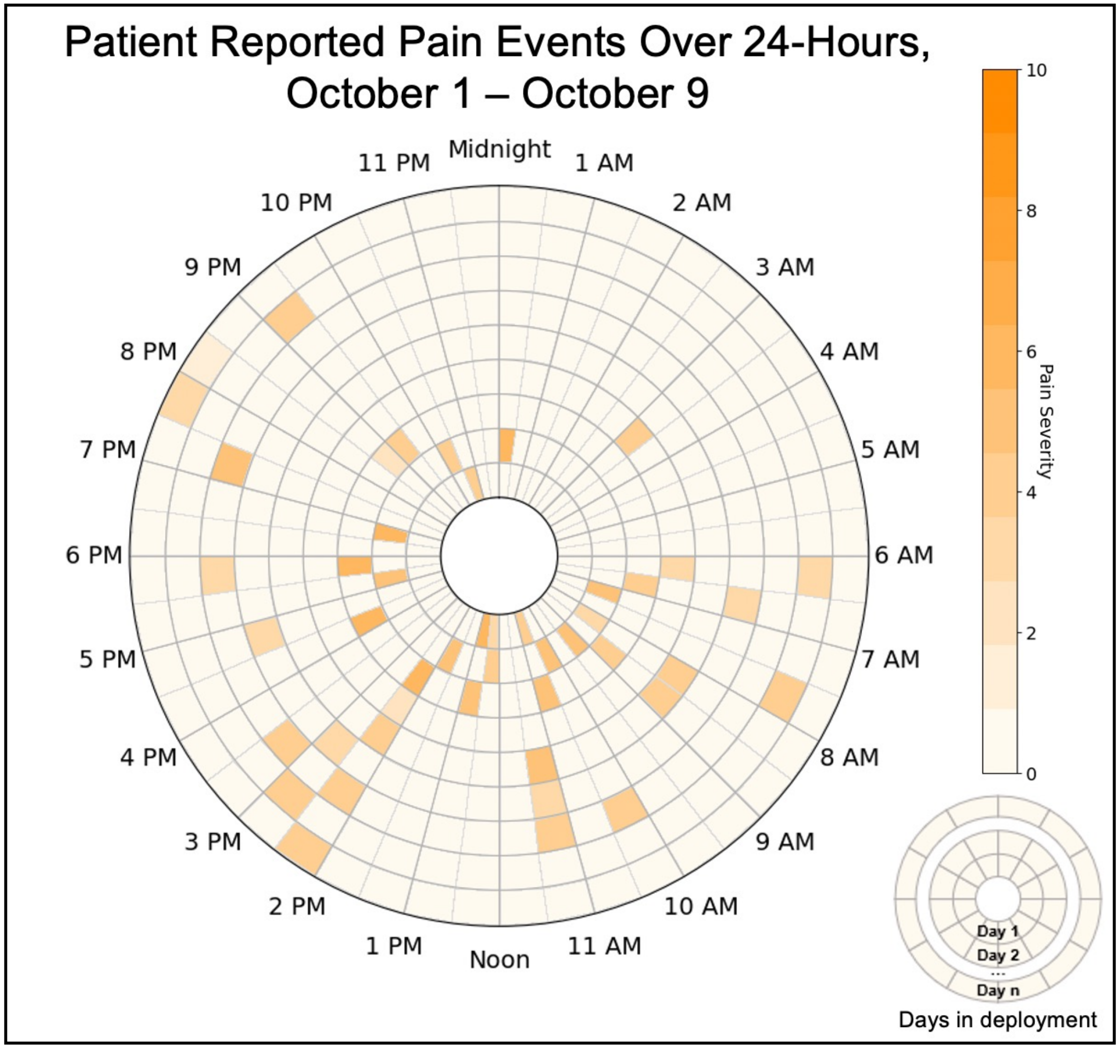

Supplement: Supplementary file 1 [file Datasheet1.pdf]
