# Supplementary material for: “Less words, more pictures”: creating and sharing data visualizations from a remote health monitoring system with clinicians to improve cancer pain management
Source: Front Digit Health. 2025 Apr 23;7:1520990. doi: 10.3389/fdgth.2025.1520990 (PMC12055813; doi:10.3389/fdgth.2025.1520990)
Supplement: Supplementary file 2 [file Datasheet2.docx]

**Data Supplement 2:** Qualtrics survey used to evaluate data visualizations during a clinician feedback session.

Data Visualization Survey

Start of Block: Default Question Block

Consent **Characterizing the Complexity of Cancer Pain in the Home - IRB #21017**

The University of Virginia School of Nursing is doing a study about using technology to monitor cancer pain at home. You are being asked to participate because you are a healthcare provider who cares for patients who may be experiencing cancer pain.

 **The purpose of this research study** is to test the use of an in-home sensor system called BESI-C, or Behavioral and Environmental Sensing and Intervention for Cancer. This system uses a combination of wearable (smart watch) and environmental sensors to collect information about pain from patients with cancer and their family caregivers in the home. Once the system collects the data, we are trying to figure out the best way to share the results with key stakeholders – patients, family caregivers, and healthcare providers.

 **If you agree to participate**, we will ask you to review data visualizations created by the BESI-C system and provide suggestions and feedback about what you find helpful. Depending on what is feasible, we may do this in-person or over Zoom or other similar virtual platform. Also, depending on what is feasible and convenient, we may conduct the feedback session with you as an individual or as a small group of healthcare providers.

 A trained study team member will ask you a series of questions about the data visualizations. You may also be asked to complete a brief questionnaire so we understand a bit about your background and your opinions regarding the data visualizations. With your permission, we may record the feedback session so we can be sure we accurately record your comments and feedback. It is anticipated that the feedback session will take approximately 45 minutes – 1 hour.

 You may be invited to participate in additional feedback sessions as we plan to revise the data visualizations throughout the study based on participant feedback.

 **Risks and Benefits**:  The primary risk of being in this study is the potential inconvenience of participating in a feedback session. However, our study team will do everything possible to make the experience as convenient as possible. You may not benefit directly from being in this study, but you will be providing helpful information to improve cancer care and support healthcare providers.

 **Compensation:** You will be given a $25 gift card for participating in a feedback session. You do not have to be in this study if you do not want to participate. 

 Your decision to be in any study is totally voluntary. Thank you for your consideration!
By selecting "Yes, I consent" you are agreeing to the terms above.

If you choose not to consent, you will be exited from the this survey and we would ask you to please exit the Zoom meeting.

- Yes, I consent
- No, I do not consent

Skip To: End of Survey If Characterizing the Complexity of Cancer Pain in the Home - IRB #21017 The University of Virginia... = No, I do not consent

End of Block: Default Question Block

Start of Block: Part 1.  Please answer the questions below about yourself.

Q6 Are you involved in the clinical care of patients with cancer pain?

- Yes
- No

Skip To: End of Survey If Are you involved in the clinical care of patients with cancer pain? = No

Q3 With which gender do you identify?

- Man
- Woman
- Non-binary
- Prefer to provide own description __________________________________________________
- Prefer not to answer

Q4 What is your age?

- 18 - 24
- 25 - 34
- 35 - 44
- 45 - 54
- 55 - 64
- 65 - 74
- 75 - 84
- 85 or older

Q5 Which category best describes your current clinical role?

- Registered Nurse (RN)
- Physician (MD)
- Advanced Practice Registered Nurse (NP, CNS)
- Social Worker
- Chaplain
- Pre-licensure student (nursing student or medical student)
- Other __________________________________________________

Q9 How long have you been providing clinical care to patients with cancer?

- Less than 1 year
- 1 - 5 years
- 6 - 11 years
- More than 11 years

Q7 Which racial and ethnicity group(s) do you feel best describes you? (*can select more than one option)

- White
- Black or African American
- American Indian or Alaska Native
- Asian
- Native Hawaiian or Pacific Islander
- Hispanic
- Prefer to provide own description __________________________________________________
- Prefer not to answer

Q13 How are you participating in this meeting?

- With others, in a conference room viewing a shared screen
- By myself, on my laptop or desktop viewing an individual screen

End of Block: Part 1.  Please answer the questions below about yourself.

Start of Block: Block 2

Q10
****PAUSE AND LOOK UP*****


**Please look at Data Visualization #1,** which is being shared by the study team.

End of Block: Block 2

Start of Block: Block 3

Q11 Please answer the following questions about **Data Visualization #1.**

DV1.1 **Data Visualization #1...**

|  | Strongly Agree | Agree | Disagree | Strongly Disagree | Unsure |
| --- | --- | --- | --- | --- | --- |
| is easy to understand |  |  |  |  |  |
| would help me make clinical decisions |  |  |  |  |  |
| would save me time providing clinical care |  |  |  |  |  |

Q29 **Data Visualization #1:**  During what time period did the patient report the FEWEST pain events?

- Midnight - 4 am
- 7am - 11am
- 5pm - 9pm
- Don't know/unclear

| Page Break |  |
| --- | --- |

DV1.2
**Data Visualization #1 - Group Discussion**

| Page Break |  |
| --- | --- |

Q31
****PAUSE AND LOOK UP*****
 
**Please look at Data Visualization #2,** which is being shared by the study team.

| Page Break |  |
| --- | --- |

Q32 Please answer the following questions about **Data Visualization #2.**

Q21 **Data Visualization #2...**

|  | Strongly Agree | Agree | Disagree | Strongly Disagree | Unsure |
| --- | --- | --- | --- | --- | --- |
| is easy to understand |  |  |  |  |  |
| would help me make clinical decisions |  |  |  |  |  |
| would save me time providing clinical care |  |  |  |  |  |

Q28 **Data Visualization #2:** What day did the patient report the MOST pain events ≥ 5?

- October 9
- October 14
- October 19
- Don't know/unclear

| Page Break |  |
| --- | --- |

Q30
**Data Visualization #2 - Group Discussion**

| Page Break |  |
| --- | --- |

Q33
*****PAUSE AND LOOK UP*****
 
Please look at **Data Visualization #3**, being shared by the study team.

| Page Break |  |
| --- | --- |

Q34 Please answer the following questions about **Data Visualization #3.**

Q22 **Data Visualization #3...**

|  | Strongly Agree | Agree | Disagree | Strongly Disagree | Unsure |
| --- | --- | --- | --- | --- | --- |
| is easy to understand |  |  |  |  |  |
| would help me make clinical decisions |  |  |  |  |  |
| would save me time providing clinical care |  |  |  |  |  |

Q27 **Data Visualization #3:** What is the most common reason that the patient did NOT take an opioid?

- Pain not bad enough
- Not time yet
- Side effects
- Don't know/unclear

| Page Break |  |
| --- | --- |

Q35 **Data Visualization #3** - Small Group Discussion

| Page Break |  |
| --- | --- |

Q36 *****PAUSE AND LOOK UP*****

 Please look at **Data Visualization #4,** being shared by the study team.

| Page Break |  |
| --- | --- |

Q37 Please answer the following questions about **Data Visualization #4.**

Q23 **Data Visualization #4...**

|  | Strongly Agree | Agree | Disagree | Strongly Disagree | Unsure |
| --- | --- | --- | --- | --- | --- |
| is easy to understand |  |  |  |  |  |
| would help me make clinical decisions |  |  |  |  |  |
| would save me time providing clinical care |  |  |  |  |  |

Q26 **Data Visualization #4:** From the **caregiver's** perspective, which environmental factor had the strongest relationship with more severe patient pain?

- Humidity level
- Air pressure fluctuations
- Noise level
- Don't know/unclear

| Page Break |  |
| --- | --- |

Q38 **Data Visualization #4** - Small Group Discussion

| Page Break |  |
| --- | --- |

Q39
*****PAUSE AND LOOK UP*****
 
Please look at **Data Visualization #5**, being shared by the study team.

| Page Break |  |
| --- | --- |

Q40 Please answer the following questions about **Data Visualization #5.**

Q24 **Data Visualization #5...**

|  | Strongly Agree | Agree | Disagree | Strongly Disagree | Unsure |
| --- | --- | --- | --- | --- | --- |
| is easy to understand |  |  |  |  |  |
| would help me make clinical decisions |  |  |  |  |  |
| would save me time providing clinical care |  |  |  |  |  |

Q25 **Data Visualization #5:** How would you rate the impact of opioids on decreasing the patient's pain?

- High
- Moderate
- Low
- Don't know/unclear

| Page Break |  |
| --- | --- |

Q41 **Data Visualization #5 -** Small Group Discussion

| Page Break |  |
| --- | --- |

End of Block: Block 3

Data Visualization Survey – Session 2

Start of Block: Default Question Block

Consent **Characterizing the Complexity of Cancer Pain in the Home - IRB #21017**

 The XXX School of Nursing is doing a study about using technology to monitor cancer pain at home. You are being asked to participate because you are a healthcare provider who cares for patients who may be experiencing cancer pain.

 **The purpose of this research study** is to test the use of an in-home sensor system called BESI-C, or Behavioral and Environmental Sensing and Intervention for Cancer. This system uses a combination of wearable (smart watch) and environmental sensors to collect information about pain from patients with cancer and their family caregivers in the home. Once the system collects the data, we are trying to figure out the best way to share the results with key stakeholders – patients, family caregivers, and healthcare providers.

 **If you agree to participate**, we will ask you to review data visualizations created by the BESI-C system and provide suggestions and feedback about what you find helpful. Depending on what is feasible, we may do this in-person or over Zoom or other similar virtual platform. Also, depending on what is feasible and convenient, we may conduct the feedback session with you as an individual or as a small group of healthcare providers.

 A trained study team member will ask you a series of questions about the data visualizations. You may also be asked to complete a brief questionnaire so we understand a bit about your background and your opinions regarding the data visualizations. With your permission, we may record the feedback session so we can be sure we accurately record your comments and feedback. It is anticipated that the feedback session will take approximately 45 minutes – 1 hour.

 You may be invited to participate in additional feedback sessions as we plan to revise the data visualizations throughout the study based on participant feedback.

 **Risks and Benefits**:  The primary risk of being in this study is the potential inconvenience of participating in a feedback session. However, our study team will do everything possible to make the experience as convenient as possible. You may not benefit directly from being in this study, but you will be providing helpful information to improve cancer care and support healthcare providers.

 **Compensation:** You will be given a $20 gift card for participating in a feedback session. You do not have to be in this study if you do not want to participate. 

 Your decision to be in any study is totally voluntary. Thank you for your consideration!
By selecting "Yes, I consent" you are agreeing to the terms above.

If you choose not to consent, you will be exited from the this survey and we would ask you to please exit the Zoom meeting.

- Yes, I consent (1)
- No, I do not consent (2)

Skip To: End of Survey If Characterizing the Complexity of Cancer Pain in the Home - IRB #21017 The University of Virginia... = No, I do not consent

End of Block: Default Question Block

Start of Block: Part 1.  Please answer the questions below about yourself.

Q6 Are you involved in the clinical care of patients with cancer pain?

- Yes (1)
- No (2)

Skip To: End of Survey If Are you involved in the clinical care of patients with cancer pain? = No

Q3 With which gender do you identify?

- Man (1)
- Woman (2)
- Non-binary (3)
- Prefer to provide own description (4) __________________________________________________
- Prefer not to answer (6)

Q4 What is your age?

- 18 - 24 (1)
- 25 - 34 (2)
- 35 - 44 (3)
- 45 - 54 (4)
- 55 - 64 (5)
- 65 - 74 (6)
- 75 - 84 (7)
- 85 or older (8)

Q5 Which category best describes your current clinical role?

- Registered Nurse (RN) (1)
- Physician (MD) (2)
- Advanced Practice Registered Nurse (NP, CNS) (3)
- Social Worker (4)
- Chaplain (5)
- Pre-licensure student (nursing student or medical student) (6)
- Other (7) __________________________________________________

Q9 How long have you been providing clinical care to patients with cancer?

- Less than 1 year (1)
- 1 - 5 years (2)
- 6 - 11 years (3)
- More than 11 years (4)

Q7 Which racial and ethnicity group(s) do you feel best describes you? (*can select more than one option)

- White (1)
- Black or African American (2)
- American Indian or Alaska Native (3)
- Asian (4)
- Native Hawaiian or Pacific Islander (5)
- Hispanic (9)
- Prefer to provide own description (10) __________________________________________________
- Prefer not to answer (11)

Q13 How are you participating in this meeting?

- With others, in a conference room viewing a shared screen (1)
- By myself, on my laptop or desktop viewing an individual screen (2)

End of Block: Part 1.  Please answer the questions below about yourself.

Start of Block: Block 2

Q10
****PAUSE AND LOOK UP*****


**Please look at Data Visualization #1,** which is being shared by the study team.

End of Block: Block 2

Start of Block: Block 3

Q11 Please answer the following questions about **Data Visualization #1.**

DV1.1 **Data Visualization #1...**

|  | Strongly Agree (1) | Agree (2) | Disagree (3) | Strongly Disagree (4) | Unsure (5) |
| --- | --- | --- | --- | --- | --- |
| is easy to understand (6) |  |  |  |  |  |
| would help me make clinical decisions (7) |  |  |  |  |  |
| would save me time providing clinical care (9) |  |  |  |  |  |

Q29 **Data Visualization #1:**  At 4:30pm, how distressed did the patient perceive their caregiver to be?  

- Not at all (1)
- A little (3)
- A lot (4)
- Don't know/unclear (5)

| Page Break |  |
| --- | --- |

DV1.2
**Data Visualization #1 - Interactive Demo and Group Discussion**

| Page Break |  |
| --- | --- |

Q31
****PAUSE AND LOOK UP*****
 
**Please look at Data Visualization #2,** which is being shared by the study team.

| Page Break |  |
| --- | --- |

Q32 Please answer the following questions about **Data Visualization #2.**

Q21 **Data Visualization #2...**

|  | Strongly Agree (1) | Agree (2) | Disagree (3) | Strongly Disagree (4) | Unsure (5) |
| --- | --- | --- | --- | --- | --- |
| is easy to understand (6) |  |  |  |  |  |
| would help me make clinical decisions (7) |  |  |  |  |  |
| would save me time providing clinical care (9) |  |  |  |  |  |

Q28 **Data Visualization #2:** What day did the patient report the MOST pain events ≥ 5?

- November 10 (1)
- November 14 (2)
- November 17 (3)
- Don't know/unclear (4)

| Page Break |  |
| --- | --- |

Q30
**Data Visualization #2 - Interactive Demo and Group Discussion**

| Page Break |  |
| --- | --- |

Q33
*****PAUSE AND LOOK UP*****
 
Please look at **Data Visualization #3**, being shared by the study team.

| Page Break |  |
| --- | --- |

Q34 Please answer the following questions about **Data Visualization #3.**

Q22 **Data Visualization #3...**

|  | Strongly Agree (1) | Agree (2) | Disagree (3) | Strongly Disagree (4) | Unsure (5) |
| --- | --- | --- | --- | --- | --- |
| is easy to understand (6) |  |  |  |  |  |
| would help me make clinical decisions (7) |  |  |  |  |  |
| would save me time providing clinical care (9) |  |  |  |  |  |

Q27 **Data Visualization #3:** Around what time did the patient have a pain event with the **greatest** decrease after 40 minutes?

- Around 8am (1)
- Around 4pm (2)
- Around 10pm (3)
- Don't know/unclear (4)

| Page Break |  |
| --- | --- |

Q35 **Data Visualization #3** - Interactive Demo and Discussion

| Page Break |  |
| --- | --- |

Q36 *****PAUSE AND LOOK UP*****

 Please look at **Data Visualization #4,** being shared by the study team.

| Page Break |  |
| --- | --- |

Q37 Please answer the following questions about **Data Visualization #4.**

Q23 **Data Visualization #4...**

|  | Strongly Agree (1) | Agree (2) | Disagree (3) | Strongly Disagree (4) | Unsure (5) |
| --- | --- | --- | --- | --- | --- |
| is easy to understand (6) |  |  |  |  |  |
| would help me make clinical decisions (7) |  |  |  |  |  |
| would save me time providing clinical care (9) |  |  |  |  |  |

Q26 **Data Visualization #4:**How many times did the caregiver report that the patient took an opioid on November 13th? 

- One time (1)
- Two times (2)
- Three times (3)
- Don't know/unclear (4)

| Page Break |  |
| --- | --- |

Q38 **Data Visualization #4** - Interactive Demo and Group Discussion

| Page Break |  |
| --- | --- |

Q39
*****PAUSE AND LOOK UP*****
 
Please look at **Data Visualization #5**, being shared by the study team.

| Page Break |  |
| --- | --- |

Q40 Please answer the following questions about **Data Visualization #5.**

Q24 **Data Visualization #5...**

|  | Strongly Agree (1) | Agree (2) | Disagree (3) | Strongly Disagree (4) | Unsure (5) |
| --- | --- | --- | --- | --- | --- |
| is easy to understand (6) |  |  |  |  |  |
| would help me make clinical decisions (7) |  |  |  |  |  |
| would save me time providing clinical care (9) |  |  |  |  |  |

Q25 **Data Visualization #5:** From the caregiver's perspective, which environmental factor is most related to increased patient pain?

- Room too bright (1)
- Room too warm (3)
- Room too loud (5)
- Don't know/unclear (4)

| Page Break |  |
| --- | --- |

Q41 **Data Visualization #5 -** Small Group Discussion

| Page Break |  |
| --- | --- |

Q34
*****PAUSE AND LOOK UP*****
 
Please look at **Data Visualization #6**, being shared by the study team.

| Page Break |  |
| --- | --- |

Q35 Please answer the following questions about **Data Visualization #6.**

Q36 **Data Visualization #6...**

|  | Strongly Agree (1) | Agree (2) | Disagree (3) | Strongly Disagree (4) | Unsure (5) |
| --- | --- | --- | --- | --- | --- |
| is easy to understand (6) |  |  |  |  |  |
| would help me make clinical decisions (7) |  |  |  |  |  |
| would save me time providing clinical care (9) |  |  |  |  |  |

Q37 **Data Visualization #6:** Besides taking an opioid, what else did the patient do MOST commonly to help with pain?

- Changed position (1)
- Used a hot/cold pack (3)
- Meditated (5)
- Don't know/unclear (4)

| Page Break |  |
| --- | --- |

Q38 **Data Visualization #6 -** Small Group Discussion

End of Block: Block 3

Data Visualization Survey – Session 3

Start of Block: Default Question Block

Q1 **Characterizing the Complexity of Cancer Pain in the Home - IRB #21017**

 The XXX School of Nursing is doing a study about using technology to monitor cancer pain at home. You are being asked to participate because you are a healthcare provider who cares for patients who may be experiencing cancer pain.

 **The purpose of this research study** is to test the use of an in-home sensor system called BESI-C, or Behavioral and Environmental Sensing and Intervention for Cancer. This system uses a combination of wearable (smart watch) and environmental sensors to collect information about pain from patients with cancer and their family caregivers in the home. Once the system collects the data, we are trying to figure out the best way to share the results with key stakeholders – patients, family caregivers, and healthcare providers.

 **If you agree to participate**, we will ask you to review data visualizations created by the BESI-C system and provide suggestions and feedback about what you find helpful. Depending on what is feasible, we may do this in-person or over Zoom or other similar virtual platform. Also, depending on what is feasible and convenient, we may conduct the feedback session with you as an individual or as a small group of healthcare providers.

 A trained study team member will ask you a series of questions about the data visualizations. You may also be asked to complete a brief questionnaire so we understand a bit about your background and your opinions regarding the data visualizations. With your permission, we may record the feedback session so we can be sure we accurately record your comments and feedback. It is anticipated that the feedback session will take approximately 45 minutes – 1 hour.

 You may be invited to participate in additional feedback sessions as we plan to revise the data visualizations throughout the study based on participant feedback.

 **Risks and Benefits**:  The primary risk of being in this study is the potential inconvenience of participating in a feedback session. However, our study team will do everything possible to make the experience as convenient as possible. You may not benefit directly from being in this study, but you will be providing helpful information to improve cancer care and support healthcare providers.

 **Compensation:** You will be given a $20 gift card for participating in a feedback session. You do not have to be in this study if you do not want to participate. 

 Your decision to be in any study is totally voluntary. Thank you for your consideration!
By selecting "Yes, I consent" you are agreeing to the terms above.

If you choose not to consent, you will be exited from the this survey and we would ask you to please exit the Zoom meeting.

- Yes, I DO consent (1)
- No, I do NOT consent (2)

Display This Question:

If Characterizing the Complexity of Cancer Pain in the Home - IRB #21017 The University of Virginia... = No, I do NOT consent

Q2
Based on your answer to the previous question you are ineligible to participate in this study.  Please exit the Zoom meeting. 


Thank you very much for your time!

End of Block: Default Question Block

Start of Block: Part 1.  Please answer the questions below about yourself.

Q3 Are you involved in the clinical care of patients with cancer pain?

- Yes (1)
- No (2)

Display This Question:

If Are you involved in the clinical care of patients with cancer pain? = No

Q4
Based on your answer to the previous question, you are ineligible to participate in this study.  Please exit the Zoom meeting.  

Thank you for your time!

| Page Break |  |
| --- | --- |

Q5 With which gender do you identify?

- Man (1)
- Woman (2)
- Non-binary (3)
- Prefer to provide own description (4) __________________________________________________
- Prefer not to answer (6)

Q6 What is your age?

- 18 - 24 (1)
- 25 - 34 (2)
- 35 - 44 (3)
- 45 - 54 (4)
- 55 - 64 (5)
- 65 - 74 (6)
- 75 - 84 (7)
- 85 or older (8)

Q7 Which category best describes your current clinical role?

- Registered Nurse (RN) (1)
- Physician (MD) (2)
- Advanced Practice Registered Nurse (NP, CNS) (3)
- Social Worker (4)
- Chaplain (5)
- Pre-licensure student (nursing student or medical student) (6)
- Other (7) __________________________________________________

Q8 How long have you been providing clinical care to patients with cancer?

- Less than 1 year (1)
- 1 - 5 years (2)
- 6 - 11 years (3)
- More than 11 years (4)

Q9 Which racial and ethnicity group(s) do you feel best describes you? (*can select more than one option)

- White (1)
- Black or African American (2)
- American Indian or Alaska Native (3)
- Asian (4)
- Native Hawaiian or Pacific Islander (5)
- Hispanic (9)
- Prefer to provide own description (10) __________________________________________________
- Prefer not to answer (11)

Q10 How are you participating in this meeting?

- With others, in a conference room viewing a shared screen (1)
- By myself, on my laptop or desktop viewing an individual screen (2)

| Page Break |  |
| --- | --- |

Q11 Which quality of life data are generally most helpful to you when assessing a patient? 

** Please rank - drag and drop - from MOST helpful (1) to LEAST helpful (8).  NOTE: numbers will appear when you hover over the words **.

______ Sleep (1)

______ Mood (2)

______ Appetite (3)

______ Fatigue (4)

______ Activity (5)

______ Social engagement/interaction (6)

______ Overall distress score (7)

______ Write in option (8)

Q12
Which pain-related data are generally most helpful to you when assessing a patient? 

 ** Please rank - drag and drop - from MOST helpful (1) to LEAST helpful (8).  NOTE: numbers will appear when you hover over the words **.

______ Total number of times they took a PRN opioid (1)

______ Why they did not take an opioid, even if in pain (2)

______ The severity (how bad) of pain events (3)

______ The frequency (how often) of pain events (4)

______ How much the PRN opioid decreased pain (5)

______ When (specific times) they took a PRN opioid (6)

______ Distress accompanying a pain event (8)

______ Write in option (7)

Q13 How would you prefer to view patient/caregiver reported symptom data?

- As an interactive dashboard where I can change parameters and customize the data I see. (1)
- As a static document, such as a PDF attached to the patient record. (2)
- I would like to have both options. (3)
- Neither. (4)
- Another way (please write in) (6) __________________________________________________

Q14 How important is it to you that patient/caregiver reported symptom data are integrated within the electronic health record (EPIC)?

- Extremely important (1)
- Important (2)
- Neutral (3)
- A little important (4)
- Not at all important (5)

End of Block: Part 1.  Please answer the questions below about yourself.

Start of Block: Block 4

Q15 THANK YOU!  PLEASE PAUSE AND AWAIT NEXT INSTRUCTIONS.

End of Block: Block 4

Start of Block: Block 2

Q16
****PAUSE AND LOOK UP*****


**Please look at Data Visualization #1,** which is being shared by the study team.

End of Block: Block 2

Start of Block: Block 3

Q17 Please answer the following questions about **Data Visualization #1.**

Q18 **Data Visualization #1...**

|  | Strongly Agree (1) | Agree (2) | Disagree (3) | Strongly Disagree (4) | Unsure (5) |
| --- | --- | --- | --- | --- | --- |
| is easy to understand (6) |  |  |  |  |  |
| would help me make clinical decisions (7) |  |  |  |  |  |
| would save me time providing clinical care (9) |  |  |  |  |  |

Q19 **Data Visualization #1:**  Which of the following statements is TRUE?

- Patient had at least one pain event ≥ 5/10 every day over the 2-week period. (1)
- PRN opioids decreased pain in < 50% of pain events over the 2-week period. (2)
- The patient rarely uses PRN opioids to manage their pain. (3)
- Don't know/unclear (4)

| Page Break |  |
| --- | --- |

Q20
**Data Visualization #1 - Group Discussion**

Q21 **Data Visualization #1:** Is there anything else about this visualization you would like us to know? (optional)

________________________________________________________________

| Page Break |  |
| --- | --- |

Q22
****PAUSE AND LOOK UP*****
 
**Please look at Data Visualization #2,** which is being shared by the study team.

| Page Break |  |
| --- | --- |

Q23 Please answer the following questions about **Data Visualization #2.**

Q24 **Data Visualization #2...**

|  | Strongly Agree (1) | Agree (2) | Disagree (3) | Strongly Disagree (4) | Unsure (5) |
| --- | --- | --- | --- | --- | --- |
| is easy to understand (6) |  |  |  |  |  |
| would help me make clinical decisions (7) |  |  |  |  |  |
| would save me time providing clinical care (9) |  |  |  |  |  |

Q25 **Data Visualization #2:**Whose pain events does the infographic summarize, and who is providing the data?

- Patient's pain, as reported by the patient (1)
- Caregiver's pain, as reported by the caregiver (2)
- Patient's pain, as reported by the caregiver (3)
- Caregiver's pain, as reported by the patient (4)
- Don't know/unclear (5)

| Page Break |  |
| --- | --- |

Q26
**Data Visualization #2 - Group Discussion**

Q27 **Data Visualization #2:**  Is there else about this data visualization you would like us to know?  (optional)

________________________________________________________________

| Page Break |  |
| --- | --- |

Q28
*****PAUSE AND LOOK UP*****
 
Please look at **Data Visualization #3**, being shared by the study team.

| Page Break |  |
| --- | --- |

Q29 Please answer the following questions about **Data Visualization #3.**

Q30 **Data Visualization #3...**

|  | Strongly Agree (1) | Agree (2) | Disagree (3) | Strongly Disagree (4) | Unsure (5) |
| --- | --- | --- | --- | --- | --- |
| is easy to understand (6) |  |  |  |  |  |
| would help me make clinical decisions (7) |  |  |  |  |  |
| would save me time providing clinical care (9) |  |  |  |  |  |

Q31 **Data Visualization #3:** On what day(s) did the patient report the most pain events? 

- March 2 (1)
- March 14 and March 5 (2)
- March 4 and March 12 (3)
- Don't know/unclear (4)

| Page Break |  |
| --- | --- |

Q32 **Data Visualization #3** - Discussion

Q33 **Data Visualization #3:**  Is there anything else about this data visualization you would like us to know?  (optional)

________________________________________________________________

| Page Break |  |
| --- | --- |

Q34 *****PAUSE AND LOOK UP*****

 Please look at **Data Visualization #4,** being shared by the study team.

| Page Break |  |
| --- | --- |

Q35 Please answer the following questions about **Data Visualization #4.**

Q36 **Data Visualization #4...**

|  | Strongly Agree (1) | Agree (2) | Disagree (3) | Strongly Disagree (4) | Unsure (5) |
| --- | --- | --- | --- | --- | --- |
| is easy to understand (6) |  |  |  |  |  |
| would help me make clinical decisions (7) |  |  |  |  |  |
| would save me time providing clinical care (9) |  |  |  |  |  |

Q37 **Data Visualization #4:** Based on this patient quality of life report, what are most likely to be your priority concerns? 

- Appetite, fatigue, mood (1)
- Overall distress (2)
- Too much social interaction / time spent with others (3)
- Don't know/unclear (4)

| Page Break |  |
| --- | --- |

Q38 **Data Visualization #4** - Group Discussion

Q39 **Data Visualization #4:**  Is there anything else about this data visualization you would like us to know?  (optional)

________________________________________________________________

| Page Break |  |
| --- | --- |

Q40
*****PAUSE AND LOOK UP*****
 
Please look at **Data Visualization #5**, being shared by the study team.

| Page Break |  |
| --- | --- |

Q41 Please answer the following questions about **Data Visualization #5.**

Q42 **Data Visualization #5...**

|  | Strongly Agree (1) | Agree (2) | Disagree (3) | Strongly Disagree (4) | Unsure (5) |
| --- | --- | --- | --- | --- | --- |
| is easy to understand (6) |  |  |  |  |  |
| would help me make clinical decisions (7) |  |  |  |  |  |
| would save me time providing clinical care (9) |  |  |  |  |  |

Q43 **Data Visualization #5:** Based on this caregiver quality of life report, what is your key 'take-away?'

- This caregiver is highly distressed and needs immediate support. (1)
- This caregiver may have limited social interaction with others. (3)
- This caregiver has had significantly more 'bad' days than 'good' days. (5)
- Don't know/unclear (4)

| Page Break |  |
| --- | --- |

Q44 **Data Visualization #5 -** Discussion

Q45 **Data Visualization #5:**  Is there anything else about this visualization you would like us to know?  (optional)

________________________________________________________________

| Page Break |  |
| --- | --- |

End of Block: Block 3

Data Visualization Survey – Session 4

Start of Block: Default Question Block

Q1 **Characterizing the Complexity of Cancer Pain in the Home - IRB #21017**

 The XXX School of Nursing is doing a study about using technology to monitor cancer pain at home. You are being asked to participate because you are a healthcare provider who cares for patients who may be experiencing cancer pain.

 **The purpose of this research study** is to test the use of an in-home sensor system called BESI-C, or Behavioral and Environmental Sensing and Intervention for Cancer. This system uses a combination of wearable (smart watch) and environmental sensors to collect information about pain from patients with cancer and their family caregivers in the home. Once the system collects the data, we are trying to figure out the best way to share the results with key stakeholders – patients, family caregivers, and healthcare providers.

 **If you agree to participate**, we will ask you to review data visualizations created by the BESI-C system and provide suggestions and feedback about what you find helpful. Depending on what is feasible, we may do this in-person or over Zoom or other similar virtual platform. Also, depending on what is feasible and convenient, we may conduct the feedback session with you as an individual or as a small group of healthcare providers.

 A trained study team member will ask you a series of questions about the data visualizations. You may also be asked to complete a brief questionnaire so we understand a bit about your background and your opinions regarding the data visualizations. With your permission, we may record the feedback session so we can be sure we accurately record your comments and feedback. It is anticipated that the feedback session will take approximately 45 minutes – 1 hour.

 You may be invited to participate in additional feedback sessions as we plan to revise the data visualizations throughout the study based on participant feedback.

 **Risks and Benefits**:  The primary risk of being in this study is the potential inconvenience of participating in a feedback session. However, our study team will do everything possible to make the experience as convenient as possible. You may not benefit directly from being in this study, but you will be providing helpful information to improve cancer care and support healthcare providers.

 **Compensation:** You will be given a $25 gift card for participating in a feedback session, if allowed by your institution. You do not have to be in this study if you do not want to participate. 

 Your decision to be in any study is totally voluntary. Thank you for your consideration!
By selecting "Yes, I consent" you are agreeing to the terms above.

If you choose not to consent, you will be exited from the this survey and we would ask you to please exit the meeting.

- Yes, I DO consent (1)
- No, I do NOT consent (2)

Display This Question:

If Characterizing the Complexity of Cancer Pain in the Home - IRB #21017 The University of Virginia... = No, I do NOT consent

Q2
Based on your answer to the previous question you are ineligible to participate in this study.  Please exit the meeting. 


Thank you very much for your time!

End of Block: Default Question Block

Start of Block: Part 1.  Please answer the questions below about yourself.

Q3 Are you involved in the clinical care of patients with cancer pain?

- Yes (1)
- No (2)

Display This Question:

If Are you involved in the clinical care of patients with cancer pain? = No

Q4
Based on your answer to the previous question, you are ineligible to participate in this study.  Please exit the meeting.  


Thank you for your time!

| Page Break |  |
| --- | --- |

Q5 With which gender do you identify?

- Man (1)
- Woman (2)
- Non-binary (3)
- Prefer to provide own description (4) __________________________________________________
- Prefer not to answer (6)

Q6 What is your age?

- 18 - 24 (1)
- 25 - 34 (2)
- 35 - 44 (3)
- 45 - 54 (4)
- 55 - 64 (5)
- 65 - 74 (6)
- 75 - 84 (7)
- 85 or older (8)

Q7 Which category best describes your current clinical role?

- Registered Nurse (RN) (1)
- Physician (MD) (2)
- Advanced Practice Registered Nurse (NP, CNS) (3)
- Social Worker (4)
- Chaplain (5)
- Pre-licensure student (nursing student or medical student) (6)
- Other (7) __________________________________________________

Q8 How long have you been providing clinical care to patients with cancer?

- Less than 1 year (1)
- 1 - 5 years (2)
- 6 - 11 years (3)
- More than 11 years (4)

Q9 Which racial and ethnicity group(s) do you feel best describes you? (*can select more than one option)

- White (1)
- Black or African American (2)
- American Indian or Alaska Native (3)
- Asian (4)
- Native Hawaiian or Pacific Islander (5)
- Hispanic (9)
- Prefer to provide own description (10) __________________________________________________
- Prefer not to answer (11)

| Page Break |  |
| --- | --- |

Q11 Which quality of life data are generally most helpful to you when assessing a patient? 

** Please rank - drag and drop - from MOST helpful (1) to LEAST helpful (8).  NOTE: numbers will appear when you hover over the words **.

______ Sleep (1)

______ Mood (2)

______ Appetite (3)

______ Fatigue (4)

______ Activity (5)

______ Social engagement/interaction (6)

______ Overall distress score (7)

______ Write in option (8)

Q12
Which pain-related data are generally most helpful to you when assessing a patient? 

 ** Please rank - drag and drop - from MOST helpful (1) to LEAST helpful (8).  NOTE: numbers will appear when you hover over the words **.

______ Total number of times they took a PRN opioid (1)

______ Why they did not take an opioid, even if in pain (2)

______ The severity (how bad) of pain events (3)

______ The frequency (how often) of pain events (4)

______ How much the PRN opioid decreased pain (5)

______ When (specific times) they took a PRN opioid (6)

______ Distress accompanying a pain event (8)

______ Write in option (7)

Q13 How would you prefer to view patient/caregiver reported symptom data?

- As an interactive dashboard where I can change parameters and customize the data I see. (1)
- As a static document, such as a PDF attached to the patient record. (2)
- I would like to have both options. (3)
- Neither. (4)
- Another way (please write in) (6) __________________________________________________

Q14 How important is it to you that patient/caregiver reported symptom data are integrated within the electronic health record (EPIC)?

- Extremely important (1)
- Important (2)
- Neutral (3)
- A little important (4)
- Not at all important (5)

End of Block: Part 1.  Please answer the questions below about yourself.

Start of Block: Block 4

Q15 THANK YOU!  PLEASE PAUSE AND AWAIT NEXT INSTRUCTIONS.

End of Block: Block 4

Start of Block: Block 2

Q16
****PAUSE AND LOOK UP*****


**Please look at Data Visualization #1 in your packet.**

End of Block: Block 2

Start of Block: Block 3

Q17 Please answer the following questions about **Data Visualization #1.**

| Page Break |  |
| --- | --- |

Q18 **Data Visualization #1...**

|  | Strongly Agree (1) | Agree (2) | Disagree (3) | Strongly Disagree (4) | Unsure (5) |
| --- | --- | --- | --- | --- | --- |
| is easy to understand (6) |  |  |  |  |  |
| would help me make clinical decisions (7) |  |  |  |  |  |
| would save me time providing clinical care (9) |  |  |  |  |  |

| Page Break |  |
| --- | --- |

Q54 **Data Visualization #1:** Based on my clinical experience, this visualization would be helpful for patients and family caregivers.

- Strongly agree (9)
- Agree (10)
- Disagree (11)
- Strongly Disagree (12)
- Unsure (13)

| Page Break |  |
| --- | --- |

Q19 **Data Visualization #1:**  Which of the following statements is TRUE?

- Patient had at least one pain event ≥ 5/10 every day over the 2-week period. (1)
- Opioids decreased pain in < 50% of pain events over the 2-week period. (2)
- The patient rarely uses opioids to manage their pain. (3)
- Don't know/unclear (4)

| Page Break |  |
| --- | --- |

Q20
**Data Visualization #1 - Group Discussion**

| Page Break |  |
| --- | --- |

Q21 **Data Visualization #1:** Is there anything else about this visualization you would like us to know? (optional)

________________________________________________________________

| Page Break |  |
| --- | --- |

Q22
****PAUSE AND LOOK UP*****
 
**Please look at Data Visualization #2 in your packet.**

| Page Break |  |
| --- | --- |

Q23 Please answer the following questions about **Data Visualization #2.**

| Page Break |  |
| --- | --- |

Q24 **Data Visualization #2...**

|  | Strongly Agree (1) | Agree (2) | Disagree (3) | Strongly Disagree (4) | Unsure (5) |
| --- | --- | --- | --- | --- | --- |
| is easy to understand (6) |  |  |  |  |  |
| would help me make clinical decisions (7) |  |  |  |  |  |
| would save me time providing clinical care (9) |  |  |  |  |  |

| Page Break |  |
| --- | --- |

Q53 **Data Visualization #2:** Based on my clinical experience, this visualization would be helpful for patients and family caregivers.

- Strongly agree (9)
- Agree (10)
- Disagree (11)
- Strongly Disagree (12)
- Unsure (13)

| Page Break |  |
| --- | --- |

Q25 **Data Visualization #2:**Whose pain events does the infographic summarize, and who is providing the data?

- Patient's pain, as reported by the patient (1)
- Caregiver's pain, as reported by the caregiver (2)
- Patient's pain, as reported by the caregiver (3)
- Caregiver's pain, as reported by the patient (4)
- Don't know/unclear (5)

| Page Break |  |
| --- | --- |

Q26
**Data Visualization #2 - Group Discussion**

| Page Break |  |
| --- | --- |

Q27 **Data Visualization #2:**  Is there else about this data visualization you would like us to know?  (optional)

________________________________________________________________

| Page Break |  |
| --- | --- |

Q28
*****PAUSE AND LOOK UP*****
 
Please look at **Data Visualization #3** in your packet.

| Page Break |  |
| --- | --- |

Q29 Please answer the following questions about **Data Visualization #3.**

| Page Break |  |
| --- | --- |

Q30 **Data Visualization #3...**

|  | Strongly Agree (1) | Agree (2) | Disagree (3) | Strongly Disagree (4) | Unsure (5) |
| --- | --- | --- | --- | --- | --- |
| is easy to understand (6) |  |  |  |  |  |
| would help me make clinical decisions (7) |  |  |  |  |  |
| would save me time providing clinical care (9) |  |  |  |  |  |

| Page Break |  |
| --- | --- |

Q55 **Data Visualization #3:**  In my clinical experience, this visualization would be helpful to share with patients and family caregivers.

- Strongly agree (9)
- Agree (10)
- Disagree (11)
- Strongly Disagree (12)
- Unsure (13)

| Page Break |  |
| --- | --- |

Q31 **Data Visualization #3:** Which statement below is TRUE?

- On Day 12, the patient reported 1 severe pain event. (2)
- On Day 1, the patient reported 3 total pain events, 2 of them were severe. (1)
- On Day 2, the patient reported the most total pain events. (3)
- Don't know/unclear (4)

| Page Break |  |
| --- | --- |

Q32 **Data Visualization #3** - Discussion

| Page Break |  |
| --- | --- |

Q33 **Data Visualization #3:**  Is there anything else about this data visualization you would like us to know?  (optional)

________________________________________________________________

| Page Break |  |
| --- | --- |

Q34 *****PAUSE AND LOOK UP*****

 Please look at **Data Visualization #4** in your packet.

| Page Break |  |
| --- | --- |

Q35 Please answer the following questions about **Data Visualization #4.**

| Page Break |  |
| --- | --- |

Q36 **Data Visualization #4...**

|  | Strongly Agree (1) | Agree (2) | Disagree (3) | Strongly Disagree (4) | Unsure (5) |
| --- | --- | --- | --- | --- | --- |
| is easy to understand (6) |  |  |  |  |  |
| would help me make clinical decisions (7) |  |  |  |  |  |
| would save me time providing clinical care (9) |  |  |  |  |  |

| Page Break |  |
| --- | --- |

Q56 **Data Visualization #4:**  In my clinical experience, this visualization would be helpful to share with patients and family caregivers.

- Strongly agree (9)
- Agree (10)
- Disagree (11)
- Strongly Disagree (12)
- Unsure (13)

| Page Break |  |
| --- | --- |

Q37 **Data Visualization #4:** Based on this patient quality of life report, what are most likely to be your priority concerns? 

- Appetite, fatigue, mood (1)
- Overall distress (2)
- Too much social interaction / time spent with others (3)
- Don't know/unclear (4)

| Page Break |  |
| --- | --- |

Q38 **Data Visualization #4** - Group Discussion

| Page Break |  |
| --- | --- |

Q39 **Data Visualization #4:**  Is there anything else about this data visualization you would like us to know?  (optional)

________________________________________________________________

| Page Break |  |
| --- | --- |

Q40
*****PAUSE AND LOOK UP*****
 
Please look at **Data Visualization #5** in your packet.

| Page Break |  |
| --- | --- |

Q41 Please answer the following questions about **Data Visualization #5.**

| Page Break |  |
| --- | --- |

Q42 **Data Visualization #5...**

|  | Strongly Agree (1) | Agree (2) | Disagree (3) | Strongly Disagree (4) | Unsure (5) |
| --- | --- | --- | --- | --- | --- |
| is easy to understand (6) |  |  |  |  |  |
| would help me make clinical decisions (7) |  |  |  |  |  |
| would save me time providing clinical care (9) |  |  |  |  |  |

| Page Break |  |
| --- | --- |

Q57 **Data Visualization #5:**  In my clinical experience, this visualization would be helpful to share with patients and family caregivers.

- Strongly agree (9)
- Agree (10)
- Disagree (11)
- Strongly Disagree (12)
- Unsure (13)

| Page Break |  |
| --- | --- |

Q43 **Data Visualization #5:** Based on this caregiver quality of life report, what is your key 'take-away?'

- This caregiver is highly distressed and needs immediate support. (1)
- This caregiver may have limited social interaction with others. (3)
- This caregiver has had significantly more 'bad' days than 'good' days. (5)
- Don't know/unclear (4)

| Page Break |  |
| --- | --- |

Q44 **Data Visualization #5 -** Discussion

| Page Break |  |
| --- | --- |

Q45 **Data Visualization #5:**  Is there anything else about this visualization you would like us to know?  (optional)

________________________________________________________________

| Page Break |  |
| --- | --- |

Q64
*****PAUSE AND LOOK UP*****
 
Please look at **Data Visualization #6** in your packet.

| Page Break |  |
| --- | --- |

Q58 Please answer the following questions about **Data Visualization #6.**

| Page Break |  |
| --- | --- |

Q59 **Data Visualization #6...**

|  | Strongly Agree (1) | Agree (2) | Disagree (3) | Strongly Disagree (4) | Unsure (5) |
| --- | --- | --- | --- | --- | --- |
| is easy to understand (6) |  |  |  |  |  |
| would help me make clinical decisions (7) |  |  |  |  |  |
| would save me time providing clinical care (9) |  |  |  |  |  |

| Page Break |  |
| --- | --- |

Q60 **Data Visualization #6:**  In my clinical experience, this visualization would be helpful to share with patients and family caregivers.

- Strongly agree (9)
- Agree (10)
- Disagree (11)
- Strongly Disagree (12)
- Unsure (13)

| Page Break |  |
| --- | --- |

Q63 **Data Visualization #6:** During what time period did the patient report the FEWEST pain events?

- Midnight to 4am (1)
- 7am - 11am (3)
- 5pm - 9pm (5)
- Don't know/unclear (4)

| Page Break |  |
| --- | --- |

Q61 **Data Visualization #6 -** Discussion

| Page Break |  |
| --- | --- |

Q62 **Data Visualization #6:**  Is there anything else about this visualization you would like us to know?  (optional)

________________________________________________________________

| Page Break |  |
| --- | --- |

End of Block: Block 3

Data Visualization Survey – Session 5

Start of Block: Default Question Block

Q1 **Characterizing the Complexity of Cancer Pain in the Home - IRB #21017**

 The XXX School of Nursing is doing a study about using technology to monitor cancer pain at home. You are being asked to participate because you are a healthcare provider who cares for patients who may be experiencing cancer pain.

 **The purpose of this research study** is to test the use of an in-home sensor system called BESI-C, or Behavioral and Environmental Sensing and Intervention for Cancer. This system uses a combination of wearable (smart watch) and environmental sensors to collect information about pain from patients with cancer and their family caregivers in the home. Once the system collects the data, we are trying to figure out the best way to share the results with key stakeholders – patients, family caregivers, and healthcare providers.

 **If you agree to participate**, we will ask you to review data visualizations created by the BESI-C system and provide suggestions and feedback about what you find helpful. Depending on what is feasible, we may do this in-person or over Zoom or other similar virtual platform. Also, depending on what is feasible and convenient, we may conduct the feedback session with you as an individual or as a small group of healthcare providers.

 A trained study team member will ask you a series of questions about the data visualizations. You may also be asked to complete a brief questionnaire so we understand a bit about your background and your opinions regarding the data visualizations. With your permission, we may record the feedback session so we can be sure we accurately record your comments and feedback. It is anticipated that the feedback session will take approximately 45 minutes – 1 hour.

 You may be invited to participate in additional feedback sessions as we plan to revise the data visualizations throughout the study based on participant feedback.

 **Risks and Benefits**:  The primary risk of being in this study is the potential inconvenience of participating in a feedback session. However, our study team will do everything possible to make the experience as convenient as possible. You may not benefit directly from being in this study, but you will be providing helpful information to improve cancer care and support healthcare providers.

 **Compensation:** You will be given a $25 gift card for participating in a feedback session, if allowed by your institution. You do not have to be in this study if you do not want to participate. 

 Your decision to be in any study is totally voluntary. Thank you for your consideration!
By selecting "Yes, I consent" you are agreeing to the terms above.

If you choose not to consent, you will be exited from the this survey and we would ask you to please exit the meeting.

- Yes, I DO consent (1)
- No, I do NOT consent (2)

Display This Question:

If Characterizing the Complexity of Cancer Pain in the Home - IRB #21017 The University of Virginia... = No, I do NOT consent

Q2
Based on your answer to the previous question you are ineligible to participate in this study.  Please exit the meeting. 


Thank you very much for your time!

End of Block: Default Question Block

Start of Block: Part 1.  Please answer the questions below about yourself.

Q3 Are you involved in the clinical care of patients with cancer pain?

- Yes (1)
- No (2)

Display This Question:

If Are you involved in the clinical care of patients with cancer pain? = No

Q4
Based on your answer to the previous question, you are ineligible to participate in this study.  Please exit the meeting.  


Thank you for your time!

| Page Break |  |
| --- | --- |

Q5 With which gender do you identify?

- Man (1)
- Woman (2)
- Non-binary (3)
- Prefer to provide own description (4) __________________________________________________
- Prefer not to answer (6)

Q6 What is your age?

- 18 - 24 (1)
- 25 - 34 (2)
- 35 - 44 (3)
- 45 - 54 (4)
- 55 - 64 (5)
- 65 - 74 (6)
- 75 - 84 (7)
- 85 or older (8)

Q7 Which category best describes your current clinical role?

- Registered Nurse (RN) (1)
- Physician (MD) (2)
- Advanced Practice Registered Nurse (NP, CNS) (3)
- Social Worker (4)
- Chaplain (5)
- Pre-licensure student (nursing student or medical student) (6)
- Other (7) __________________________________________________

Q8 How long have you been providing clinical care to patients with cancer?

- Less than 1 year (1)
- 1 - 5 years (2)
- 6 - 11 years (3)
- More than 11 years (4)

Q9 Which racial and ethnicity group(s) do you feel best describes you? (*can select more than one option)

- White (1)
- Black or African American (2)
- American Indian or Alaska Native (3)
- Asian (4)
- Native Hawaiian or Pacific Islander (5)
- Hispanic (9)
- Prefer to provide own description (10) __________________________________________________
- Prefer not to answer (11)

| Page Break |  |
| --- | --- |

Q11 Which quality of life data are generally most helpful to you when assessing a patient? 

** Please rank - drag and drop - from MOST helpful (1) to LEAST helpful (8).  NOTE: numbers will appear when you hover over the words **.

______ Sleep (1)

______ Mood (2)

______ Appetite (3)

______ Fatigue (4)

______ Activity (5)

______ Social engagement/interaction (6)

______ Overall distress score (7)

______ Write in option (8)

Q12
Which pain-related data are generally most helpful to you when assessing a patient? 

 ** Please rank - drag and drop - from MOST helpful (1) to LEAST helpful (8).  NOTE: numbers will appear when you hover over the words **.

______ Total number of times they took a PRN opioid (1)

______ Why they did not take an opioid, even if in pain (2)

______ The severity (how bad) of pain events (3)

______ The frequency (how often) of pain events (4)

______ How much the PRN opioid decreased pain (5)

______ When (specific times) they took a PRN opioid (6)

______ Distress accompanying a pain event (8)

______ Write in option (7)

Q13 How would you prefer to view patient/caregiver reported symptom data?

- As an interactive dashboard where I can change parameters and customize the data I see. (1)
- As a static document, such as a PDF attached to the patient record. (2)
- I would like to have both options. (3)
- Neither. (4)
- Another way (please write in) (6) __________________________________________________

Q14 How important is it to you that patient/caregiver reported symptom data are integrated within the electronic health record (EPIC)?

- Extremely important (1)
- Important (2)
- Neutral (3)
- A little important (4)
- Not at all important (5)

End of Block: Part 1.  Please answer the questions below about yourself.

Start of Block: Block 4

Q15 THANK YOU!  PLEASE PAUSE / STOP AND AWAIT NEXT INSTRUCTIONS.

End of Block: Block 4

Start of Block: Block 2

Q16
****PAUSE AND LOOK UP*****


**Please look at Data Visualization #1 in your packet.**

End of Block: Block 2

Start of Block: Block 3

Q17 Please answer the following questions about **Data Visualization #1.**

| Page Break |  |
| --- | --- |

Q18 **Data Visualization #1...**

|  | Strongly Agree (1) | Agree (2) | Disagree (3) | Strongly Disagree (4) | Unsure (5) |
| --- | --- | --- | --- | --- | --- |
| is easy to understand (6) |  |  |  |  |  |
| would help me make clinical decisions (7) |  |  |  |  |  |
| would save me time providing clinical care (9) |  |  |  |  |  |

| Page Break |  |
| --- | --- |

Q54 **Data Visualization #1:** Based on my clinical experience, this visualization would be helpful for patients and family caregivers.

- Strongly agree (9)
- Agree (10)
- Disagree (11)
- Strongly Disagree (12)
- Unsure (13)

| Page Break |  |
| --- | --- |

Q19 **Data Visualization #1:**  Which of the following statements is TRUE?

- Patient had at least one pain event ≥ 5/10 every day over the 2-week period. (1)
- Opioids decreased pain in < 50% of pain events over the 2-week period. (2)
- The patient rarely uses opioids to manage their pain. (3)
- Don't know/unclear (4)

| Page Break |  |
| --- | --- |

Q20
**Data Visualization #1 - Group Discussion**

| Page Break |  |
| --- | --- |

Q21 **Data Visualization #1:** Is there anything else about this visualization you would like us to know? (optional)

________________________________________________________________

| Page Break |  |
| --- | --- |

Q22
****PAUSE AND LOOK UP*****
 
**Please look at Data Visualization #2 in your packet.**

| Page Break |  |
| --- | --- |

Q23 Please answer the following questions about **Data Visualization #2.**

| Page Break |  |
| --- | --- |

Q24 **Data Visualization #2...**

|  | Strongly Agree (1) | Agree (2) | Disagree (3) | Strongly Disagree (4) | Unsure (5) |
| --- | --- | --- | --- | --- | --- |
| is easy to understand (6) |  |  |  |  |  |
| would help me make clinical decisions (7) |  |  |  |  |  |
| would save me time providing clinical care (9) |  |  |  |  |  |

| Page Break |  |
| --- | --- |

Q53 **Data Visualization #2:** Based on my clinical experience, this visualization would be helpful for patients and family caregivers.

- Strongly agree (9)
- Agree (10)
- Disagree (11)
- Strongly Disagree (12)
- Unsure (13)

| Page Break |  |
| --- | --- |

Q25 **Data Visualization #2:**Whose pain events does the infographic summarize, and who is providing the data?

- Patient's pain, as reported by the patient (1)
- Caregiver's pain, as reported by the caregiver (2)
- Patient's pain, as reported by the caregiver (3)
- Caregiver's pain, as reported by the patient (4)
- Don't know/unclear (5)

| Page Break |  |
| --- | --- |

Q26
**Data Visualization #2 - Group Discussion**

| Page Break |  |
| --- | --- |

Q27 **Data Visualization #2:**  Is there else about this data visualization you would like us to know?  (optional)

________________________________________________________________

| Page Break |  |
| --- | --- |

Q28
*****PAUSE AND LOOK UP*****
 
Please look at **Data Visualization #3** in your packet.

| Page Break |  |
| --- | --- |

Q29 Please answer the following questions about **Data Visualization #3.**

| Page Break |  |
| --- | --- |

Q30 **Data Visualization #3...**

|  | Strongly Agree (1) | Agree (2) | Disagree (3) | Strongly Disagree (4) | Unsure (5) |
| --- | --- | --- | --- | --- | --- |
| is easy to understand (6) |  |  |  |  |  |
| would help me make clinical decisions (7) |  |  |  |  |  |
| would save me time providing clinical care (9) |  |  |  |  |  |

| Page Break |  |
| --- | --- |

Q55 **Data Visualization #3:**  In my clinical experience, this visualization would be helpful to share with patients and family caregivers.

- Strongly agree (9)
- Agree (10)
- Disagree (11)
- Strongly Disagree (12)
- Unsure (13)

| Page Break |  |
| --- | --- |

Q31 **Data Visualization #3:** Which statement below is TRUE?

- On Day 12, the patient reported 1 severe pain event. (2)
- On Day 9, the patient reported 2 severe pain events. (1)
- On Day 2, the patient reported the most total pain events. (3)
- Don't know/unclear (4)

| Page Break |  |
| --- | --- |

Q32 **Data Visualization #3** - Discussion

| Page Break |  |
| --- | --- |

Q33 **Data Visualization #3:**  Is there anything else about this data visualization you would like us to know?  (optional)

________________________________________________________________

| Page Break |  |
| --- | --- |

Q34 *****PAUSE AND LOOK UP*****

 Please look at **Data Visualization #4** in your packet.

| Page Break |  |
| --- | --- |

Q35 Please answer the following questions about **Data Visualization #4.**

| Page Break |  |
| --- | --- |

Q36 **Data Visualization #4...**

|  | Strongly Agree (1) | Agree (2) | Disagree (3) | Strongly Disagree (4) | Unsure (5) |
| --- | --- | --- | --- | --- | --- |
| is easy to understand (6) |  |  |  |  |  |
| would help me make clinical decisions (7) |  |  |  |  |  |
| would save me time providing clinical care (9) |  |  |  |  |  |

| Page Break |  |
| --- | --- |

Q56 **Data Visualization #4:**  In my clinical experience, this visualization would be helpful to share with patients and family caregivers.

- Strongly agree (9)
- Agree (10)
- Disagree (11)
- Strongly Disagree (12)
- Unsure (13)

| Page Break |  |
| --- | --- |

Q37 **Data Visualization #4:** Based on this patient quality of life report, what are most likely to be your priority concerns? 

- Appetite, fatigue, mood (1)
- Overall distress (2)
- Too much social interaction / time spent with others (3)
- Don't know/unclear (4)

| Page Break |  |
| --- | --- |

Q38 **Data Visualization #4** - Group Discussion

| Page Break |  |
| --- | --- |

Q39 **Data Visualization #4:**  Is there anything else about this data visualization you would like us to know?  (optional)

________________________________________________________________

| Page Break |  |
| --- | --- |

Q40
*****PAUSE AND LOOK UP*****
 
Please look at **Data Visualization #5** in your packet.

| Page Break |  |
| --- | --- |

Q41 Please answer the following questions about **Data Visualization #5.**

| Page Break |  |
| --- | --- |

Q42 **Data Visualization #5...**

|  | Strongly Agree (1) | Agree (2) | Disagree (3) | Strongly Disagree (4) | Unsure (5) |
| --- | --- | --- | --- | --- | --- |
| is easy to understand (6) |  |  |  |  |  |
| would help me make clinical decisions (7) |  |  |  |  |  |
| would save me time providing clinical care (9) |  |  |  |  |  |

| Page Break |  |
| --- | --- |

Q57 **Data Visualization #5:**  In my clinical experience, this visualization would be helpful to share with patients and family caregivers.

- Strongly agree (9)
- Agree (10)
- Disagree (11)
- Strongly Disagree (12)
- Unsure (13)

| Page Break |  |
| --- | --- |

Q43 **Data Visualization #5:** Based on this caregiver quality of life report, what is your key 'take-away?'

- This caregiver is highly distressed and needs immediate support. (1)
- This caregiver may have limited social interaction with others. (3)
- This caregiver has had significantly more 'bad' days than 'good' days. (5)
- Don't know/unclear (4)

| Page Break |  |
| --- | --- |

Q44 **Data Visualization #5 -** Discussion

| Page Break |  |
| --- | --- |

Q45 **Data Visualization #5:**  Is there anything else about this visualization you would like us to know?  (optional)

________________________________________________________________

| Page Break |  |
| --- | --- |

Q64
*****PAUSE AND LOOK UP*****
 
Please look at **Data Visualization #6** in your packet.

| Page Break |  |
| --- | --- |

Q58 Please answer the following questions about **Data Visualization #6.**

| Page Break |  |
| --- | --- |

Q59 **Data Visualization #6...**

|  | Strongly Agree (1) | Agree (2) | Disagree (3) | Strongly Disagree (4) | Unsure (5) |
| --- | --- | --- | --- | --- | --- |
| is easy to understand (6) |  |  |  |  |  |
| would help me make clinical decisions (7) |  |  |  |  |  |
| would save me time providing clinical care (9) |  |  |  |  |  |

| Page Break |  |
| --- | --- |

Q60 **Data Visualization #6:**  In my clinical experience, this visualization would be helpful to share with patients and family caregivers.

- Strongly agree (9)
- Agree (10)
- Disagree (11)
- Strongly Disagree (12)
- Unsure (13)

| Page Break |  |
| --- | --- |

Q63 **Data Visualization #6:** During what time period did the patient report the FEWEST pain events?

- Midnight to 4am (1)
- 7am - 11am (3)
- 5pm - 9pm (5)
- Don't know/unclear (4)

| Page Break |  |
| --- | --- |

Q61 **Data Visualization #6 -** Discussion

| Page Break |  |
| --- | --- |

Q62 **Data Visualization #6:**  Is there anything else about this visualization you would like us to know?  (optional)

________________________________________________________________

| Page Break |  |
| --- | --- |

End of Block: Block 3
